# The Genomes of *Oryza sativa*: A History of Duplications

**DOI:** 10.1371/journal.pbio.0030038

**Published:** 2005-02-01

**Authors:** Jun Yu, Jun Wang, Wei Lin, Songgang Li, Heng Li, Jun Zhou, Peixiang Ni, Wei Dong, Songnian Hu, Changqing Zeng, Jianguo Zhang, Yong Zhang, Ruiqiang Li, Zuyuan Xu, Shengting Li, Xianran Li, Hongkun Zheng, Lijuan Cong, Liang Lin, Jianning Yin, Jianing Geng, Guangyuan Li, Jianping Shi, Juan Liu, Hong Lv, Jun Li, Jing Wang, Yajun Deng, Longhua Ran, Xiaoli Shi, Xiyin Wang, Qingfa Wu, Changfeng Li, Xiaoyu Ren, Jingqiang Wang, Xiaoling Wang, Dawei Li, Dongyuan Liu, Xiaowei Zhang, Zhendong Ji, Wenming Zhao, Yongqiao Sun, Zhenpeng Zhang, Jingyue Bao, Yujun Han, Lingli Dong, Jia Ji, Peng Chen, Shuming Wu, Jinsong Liu, Ying Xiao, Dongbo Bu, Jianlong Tan, Li Yang, Chen Ye, Jingfen Zhang, Jingyi Xu, Yan Zhou, Yingpu Yu, Bing Zhang, Shulin Zhuang, Haibin Wei, Bin Liu, Meng Lei, Hong Yu, Yuanzhe Li, Hao Xu, Shulin Wei, Ximiao He, Lijun Fang, Zengjin Zhang, Yunze Zhang, Xiangang Huang, Zhixi Su, Wei Tong, Jinhong Li, Zongzhong Tong, Shuangli Li, Jia Ye, Lishun Wang, Lin Fang, Tingting Lei, Chen Chen, Huan Chen, Zhao Xu, Haihong Li, Haiyan Huang, Feng Zhang, Huayong Xu, Na Li, Caifeng Zhao, Shuting Li, Lijun Dong, Yanqing Huang, Long Li, Yan Xi, Qiuhui Qi, Wenjie Li, Bo Zhang, Wei Hu, Yanling Zhang, Xiangjun Tian, Yongzhi Jiao, Xiaohu Liang, Jiao Jin, Lei Gao, Weimou Zheng, Bailin Hao, Siqi Liu, Wen Wang, Longping Yuan, Mengliang Cao, Jason McDermott, Ram Samudrala, Jian Wang, Gane Ka-Shu Wong, Huanming Yang

**Affiliations:** **1**Beijing Institute of Genomics of the Chinese Academy of Sciences, Beijing Genomics Institute, Beijing Proteomics InstituteBeijingChina; **2**James D. Watson Institute of Genome Sciences of Zhejiang University, Hangzhou Genomics Institute, Key Laboratory of Genomic Bioinformatics of Zhejiang ProvinceHangzhouChina; **3**College of Life Sciences, Peking UniversityBeijingChina; **4**Institute of Theoretical Physics, Chinese Academy of SciencesBeijingChina; **5**Beijing North Computation CenterBeijingChina; **6**BioInformatics Laboratory, Institute of Computing Technology, Chinese Academy of SciencesBeijingChina; **7**Department of Statistics and Financial Mathematics, College of Mathematical Sciences, Beijing Normal UniversityBeijingChina; **8**Kunming Institute of Zoology, Chinese Academy of SciencesKunmingChina; **9**National Hybrid Rice R & D CenterChangshaChina; **10**Computational Genomics Group, Department of MicrobiologyUniversity of Washington, Seattle, WashingtonUnited States of America; **11**UW Genome Center, Department of Medicine, University of WashingtonSeattle, WashingtonUnited States of America; University of GeorgiaUnited States of America

## Abstract

We report improved whole-genome shotgun sequences for the genomes of *indica* and *japonica* rice, both with multimegabase contiguity, or almost 1,000-fold improvement over the drafts of 2002. Tested against a nonredundant collection of 19,079 full-length cDNAs, 97.7% of the genes are aligned, without fragmentation, to the mapped super-scaffolds of one or the other genome. We introduce a gene identification procedure for plants that does not rely on similarity to known genes to remove erroneous predictions resulting from transposable elements. Using the available EST data to adjust for residual errors in the predictions, the estimated gene count is at least 38,000–40,000. Only 2%–3% of the genes are unique to any one subspecies, comparable to the amount of sequence that might still be missing. Despite this lack of variation in gene content, there is enormous variation in the intergenic regions. At least a quarter of the two sequences could not be aligned, and where they could be aligned, single nucleotide polymorphism (SNP) rates varied from as little as 3.0 SNP/kb in the coding regions to 27.6 SNP/kb in the transposable elements. A more inclusive new approach for analyzing duplication history is introduced here. It reveals an ancient whole-genome duplication, a recent segmental duplication on Chromosomes 11 and 12, and massive ongoing individual gene duplications. We find 18 distinct pairs of duplicated segments that cover 65.7% of the genome; 17 of these pairs date back to a common time before the divergence of the grasses. More important, ongoing individual gene duplications provide a never-ending source of raw material for gene genesis and are major contributors to the differences between members of the grass family.

## Abstract 2

Comparative genome sequencing of *indica* and *japonica* rice reveals that duplication of genes and genomic regions has played a major part in the evolution of grass genomes

## Introduction

The importance of the rice genome is reflected in the fact that rice was the first crop plant to have its genome sequenced; astonishingly, it was sequenced by four independent research teams at Beijing Institute of Genomics [1], Syngenta [2], International Rice Genome Sequencing Project (IRGSP) [3,4,5], and Monsanto. Beijing analyzed the two parental strains, *93–11* and *PA64s,* for a popular land race of super-hybrid rice, *LYP9,* and released a 4.2x draft for *93–11,* a cultivar of the *indica* subspecies. This draft was acquired by a whole-genome shotgun (WGS) method [6]. Syngenta and IRGSP worked on *Nipponbare,* a cultivar of the *japonica* subspecies. Syngenta also used a WGS method and published a 6x draft. IRGSP used the clone-by-clone method [7] and released a 10x draft that incorporates the Syngenta data. Their publications include the finished version of Chromosomes 1, 4, and 10. These efforts have been widely hailed not only because rice feeds much of the world's population but also because rice is expected, through comparative analyses, to play a major role in understanding the grass family of crop plants [8,9,10,11,12,13]. We will report on an improved version of Beijing *indica,* which brings the coverage of the *93–11* dataset up to 6.28x. In addition, we improved Syngenta *japonica* by reassembling their sequence from the raw traces (National Center for Biotechnology Information Trace Archive; http://www.ncbi.nlm.nih.gov/Traces/trace.cgi?) and combining that information with our *93–11* assembly.

We achieved almost three orders of magnitude of improvement in long-range contiguity, and put essentially all the genes on the map, by combining the two WGS assemblies in a manner that preserves the single nucleotide polymorphism (SNP) information for *indica*–*japonica* differences. Both of these WGS assemblies were constructed independent of the information in IRGSP *japonica*. Hence, the two *japonica* assemblies allow us to compare the WGS and clone-by-clone methods objectively. By taking the clone-by-clone assembly as a “gold standard,” we can estimate the intrinsic misassembly rates for our two WGS assemblies—not just the *japonica* WGS but also the *indica* WGS, as identical assembly procedures are used and both contain 6x coverage. If we compare IRGSP *japonica* to Beijing *indica,* any increases in the discrepancy rate beyond this intrinsic misassembly rate can be attributed to *indica*–*japonica* differences. In the same spirit, genes are identified for all three assemblies using the same annotation procedures, to assess gene content differences without the methodological inconsistencies that have plagued previous comparisons. Finally, we introduce a simple method for analyzing gene duplications that resolves the contradictory claims that rice is an ancient aneuploid [14] and an ancient polyploid [15]. In the process, we demonstrate that duplication of individual genes plays a major role in the continuing evolution of the grass genomes.

Both WGS sequences, and details of our analyses, are available from our own Web site (Beijing Genomics Institute Rice Information System; http://rise.genomics.org.cn) [16]. The version of IRGSP *japonica* that we use was downloaded October 5, 2003, from GenBank and DNA Data Bank of Japan according to the guidelines at http://www.genome.arizona.edu/shotgun/rice/status and the physical map at http://rgp.dna.affrc.go.jp/IRGSP/download.

## Results

### WGS Assembly of *indica* and *japonica*

Many legitimate concerns have been raised about the differing qualities of the rice sequences that have been published [17,18] and on the idea that they must be “finished” [19,20]. Higher quality is of course a good thing, but it does come at a cost, and lost in the discussion is the reality that cost–benefit factors have always been important in sequencing. Most notably, all genome projects to date have focused primarily on the euchromatic regions that can be cloned and sequenced, even though important genes are missed as a result. For example, an essential 5.1-Mb fertility gene [21] resides in the heterochromatic Y chromosome of the *Drosophila* genome. In plant genomes, costs are primarily driven by the intergenic retrotransposon clusters [22] that account for about half of the rice genome, and even more of the larger maize (6x) and wheat (38x) genomes. Hence, our objective is merely to have all the genes assembled in one piece, without fragmentation, and anchored to the maps. A similar objective has been proposed [23,24] for crop genomes in general. Our benchmark is the set of full-length *japonica* cDNAs from the Knowledge-Based Oryza Molecular-Biological Encyclopedia [25] that contains 19,079 nonredundant cDNAs (nr-KOME).

**Figure 1:**
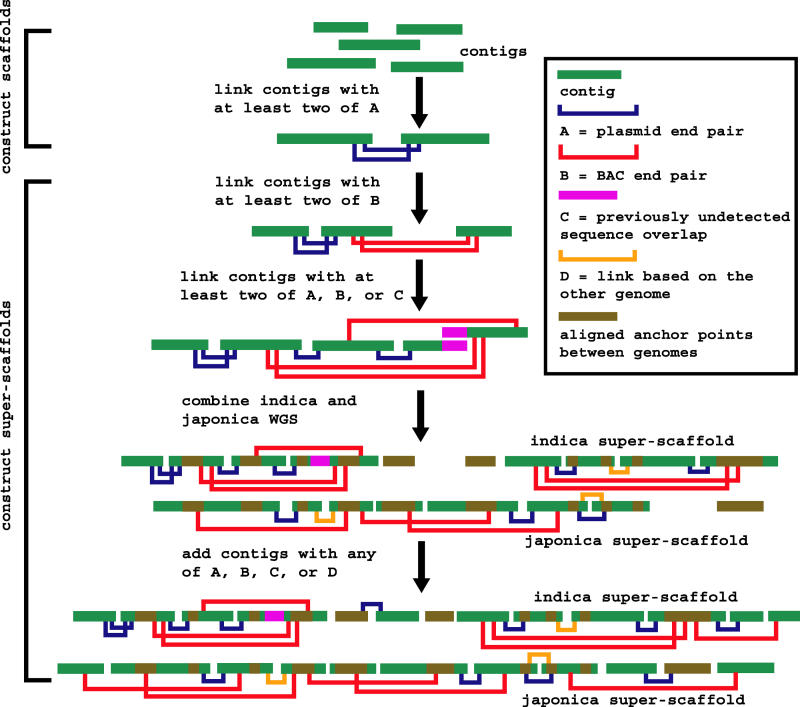
Basic Algorithm for Construction of Scaffolds and Super-Scaffolds We start with the smallest plasmids and progressively work our way up to the largest BACs. Only links with two or more pieces of supporting evidence are made. These include 34,190 “anchor points” constructed from a comparison of *indica* and *japonica*. Each anchor is a series of high-quality BlastN hits (typically 98.5% identity) put together by a dynamic programming algorithm that allows for small gaps to accommodate the polymorphic intergenic repeats. Typical anchor points contain four BlastN hits at a total size of 9 kb (including gaps). Notice how in the beginning *indica* and *japonica* are processed separately, to construct what we called scaffolds. Only at the end do we use data from one subspecies to link scaffolds in the other subspecies, and these are what we called super-scaffolds.

We begin with a few definitions. At the end of any WGS, a substantial fraction of the reads (specifically, those whose sequences are highly repeated across the genome) are invariably left unassembled. The usable reads are assembled into contigs, scaffolds, and super-scaffolds. In a contig, the identity of every base is defined. In contrast, scaffolds and super-scaffolds have gaps (regions of known length but otherwise unknown base content). The difference is that one refers to the sequence before any linking information from *indica* and *japonica* sources are combined (scaffold) and the other refers to the sequence after they are combined (super-scaffold). All of the raw data that went into these WGS assemblies are listed in Table S1, and the assembly procedure itself is outlined in Figure 1.

Compared with our previous 4.2x assembly of *indica,* more shotgun reads and a few directed finishing reads were added to increase the coverage to 6.28x. We did not use the older assembly at all. Instead, we went back to the raw reads and reassembled them, with an updated version of RePS [26,27] that incorporates some recent concepts from Phusion [28]. Increasing coverage is essential for reducing single-base error rates. Based on the estimates from RePS, 97.2% and 94.6% of our new assembly has an error rate of better than 10^−3^ and 10^−4^, respectively. For the older assembly, the percentages were only 90.8% and 83.5%, respectively. Equally important, and as expected from Poisson sampling statistics [29], increasing coverage improves the scaffold size to a point where, even without additional finishing effort, most of the nr-KOME cDNAs can be aligned in one piece, without fragmentation. All we had to do was find a way to link these scaffolds together to create larger super-scaffolds, which could then be anchored to the physical [30] and genetic [31] maps.

Mapped super-scaffolds for Beijing *indica* have a N50 size (the size above which half of the total length of a sequence dataset is found) of 8.3 Mb, which is a thousand times better than our previous draft, as shown in Table 1. We used an unorthodox method to construct super-scaffolds of megabase size from initial scaffolds of 30-kb size. Most of the increase in long-range contiguity came from combining the two WGS assemblies, not from the bacterial artificial chromosome (BAC) end pairs, which were of limited utility because their insert sizes were too large. Notice that in combining *indica* and *japonica* data, we use the alternate subspecies only for order and orientation information, not to fill missing bases. In other words, every base in the *indica* assembly is from *indica*. Not one single base is from *japonica*. Another key point is that Syngenta *japonica* is our reassembly of their raw data, not the published assembly. By using RePS for both WGS assemblies, we obtain error estimates for every base, which will later be essential for use in polymorphism detection. We would concede that if genes are ordered differently in *indica* and *japonica,* there is a small probability that by forcing the two subspecies together, we lose this information. However, there is no evidence of a major reordering of the genes because, if there were, it would have been seen in all these years of genetic mapping. The benefits thus outweigh the risks.

**Table 1:**
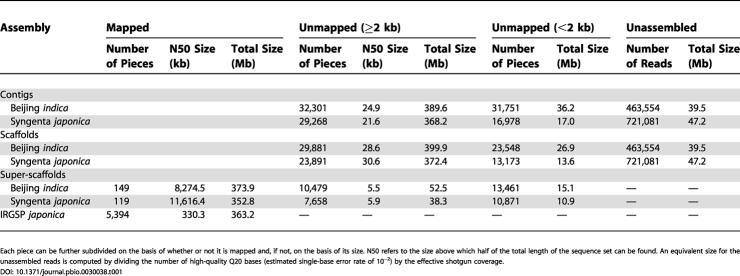
Summary of Assembled Contigs, Scaffolds, and Super-Scaffolds

Each piece can be further subdivided on the basis of whether or not it is mapped and, if not, on the basis of its size. N50 refers to the size above which half of the total length of the sequence set can be found. An equivalent size for the unassembled reads is computed by dividing the number of high-quality Q20 bases (estimated single-base error rate of 10^−2^) by the effective shotgun coverage

The total genome size, including the unassembled reads and the unmapped pieces of all sizes, is 466.3 Mb for Beijing *indica* and 433.2 Mb for Syngenta *japonica*. For this estimate, we added up all the pieces at the scaffold level (as opposed to the super-scaffold level, where the gap size estimates are taken from the alternate subspecies and may not be representative of the underlying genome). We believe this difference is real, because the two genome sizes are based on the same procedures and similar WGS datasets. Although many smaller pieces fall between the cracks in the maps, these unmapped pieces turn out to be extremely gene poor. Hence, in our submission to DNA Data Bank of Japan/European Molecular Biology Library/GenBank, we omit unassembled reads and unmapped pieces smaller than 2 kb, which has the advantage of also filtering out nonrice contaminants from inevitable mix-ups in the lab.

Physical distance is defined along a pseudo-chromosome where gaps of estimated size larger than 200 kb (a typical BAC) are collapsed to 200 kb. Between adjacent super-scaffolds, where by definition we do not have an estimated gap size, we insert a 5-kb gap. To validate the long-range accuracy of our assemblies, we compared physical and genetic distances, as shown in Figures S1 and S2. We use only those 1,519 markers that can be found in all three rice assemblies by Blastn at E-values of 10^−100^. There are two classes of discrepancies. First, the marker is on different chromosomes. All three rice assemblies agree with each other but not with the genetic map in 135 of 152 such markers. In the second class, the disagreement is on positions within a chromosome, and all three rice assemblies agree with each other but not with the genetic map in 41 of 60 such markers. Only a small handful of discrepancies are unique to any one assembly. It is highly unlikely that all three rice assemblies will make the same mistake, so we conclude that on the scale of hundreds of kilobases, our WGS data are better than the genetic map. Computed over every five markers, the mean (median) recombination rate is 4.5 (4.2) cM/Mb.

We do expect smaller-scale misassemblies in the WGS data, as, for example, in Beijing *indica,* 98.1%, 71.0%, and 39.3% of the unassembled, assembled-but-unmapped, and mapped pieces, respectively, contain 20-mer repeats that are estimated to occur at least twice in the genome. About half of these 20-mer repeats are recognizable transposable elements (TEs) in RepeatMasker (http://www.repeatmasker.org, and TE compositions in different categories of assembled data are summarized in Table S2. The most problematic misassemblies are those that occur within genes, as these affect our ability to annotate the genome. Hence, we compared the WGS data to gene sequences defined by nr-KOME and excised from IRGSP *japonica*. We searched for alignment discrepancies of at least 500 bp, consistent with misassembled reads, and interpreted any increase in the discrepancy rate from Syngenta *japonica* to Beijing *indica* as being due to polymorphic differences.

There are remarkably few genes with discrepancies in coding exons, only 0.23% in Syngenta *japonica* and 1.44% in Beijing *indica*. If we include UTR exons and introns, the rates are 0.84% in Syngenta *japonica* and 5.65% in Beijing *indica*. Hence, the implication is that WGS misassemblies occur less frequently than polymorphic differences.

Table 2 shows the number of nr-KOME cDNAs that are found in each of the three rice assemblies, using the criterion that 95% of the coding region must be alignable in BLAT [32]. Some cDNAs align to multiple pieces of the assembly, but most align to one single piece. Even if we consider only the latter case, all three rice assemblies are at least 91.2% complete. Regardless of the assembly, the gaps seem to be random, as genes that are fragmented in one assembly are often intact in another. Of the cDNAs, 98.1% can be found in one piece in either Beijing *indica* or Syngenta *japonica* (if we also insist that they be anchored to the map, this number becomes 97.7%). Combining all three rice assemblies results in 98.6% completeness. Strikingly, only 0.7% of the genes align to the unmapped Beijing *indica* sequence, despite the fact these unmapped data were 12.3% of the searched sequence. This is the first of many examples that we will provide to support the idea that the unmapped pieces are extremely gene poor.

**Table 2:**
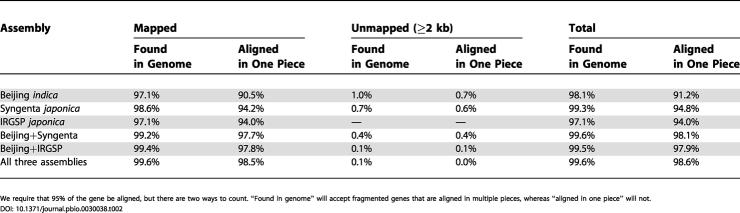
Summary of nr-KOME cDNAs with Complete Alignments (Not Including UTRs) in Each of the Three Rice Assemblies

We require that 95% of the gene be aligned, but there are two ways to count. “Found in genome” will accept fragmented genes that are aligned in multiple pieces, whereas “aligned in one piece” will not

### Gene Identification and Classification

We used an unorthodox method for gene identification. The conventional method, epitomized by Ensembl [33], uses sequence similarity to known genes and proteins to remove erroneous predictions, which are a serious problem for vertebrates because of the preponderance of large, multiexon genes, some of which can be megabases in size. However, plant genes are only a few kilobases in size, and given that *Arabidopsis* is still the only other sequenced plant, the Ensembl approach would remove many valid genes in a misguided effort to control a less serious problem. We removed erroneous predictions by relying instead on the fact most of them are actually TEs that are mistakenly called genes. Ultimately, our method is vindicated by whole-genome microarray experiments using 70-mer oligos that are hybridized to mRNA from five different tissue types. One finds that 82% of predicted rice genes with no homologs in *Arabidopsis* can be detected in this manner, as opposed to 88% of predicted rice genes with homologs (L. Ma, J. Wang, C. Chen, X. Liu, N. Su, et al., unpublished data).

For the purpose of discussion, we will classify rice genes as WH (with homolog) or NH (no homolog), based on sequence similarity to *Arabidopsis,* with the stringency set to a level that is typically found in the literature. Nucleotide sequences are translated into protein sequences, and the *Arabidopsis* genome is searched in all six reading frames using TBlastN at E-values of 10^−7^. Putative exons are chained together, and success is declared if we can account for either 50% of the protein or 100 residues. We are not concerned that more sensitive search algorithms might identify homologies that we missed. Even the best algorithms are limited in their ability to identify structural homology by sequence similarity [34]. The main objective is to show how genes that are highly homologous or nonhomologous are sufficiently different as to merit special attention in data analysis, and the simplest way to emphasize this is to draw a dividing line.

**Figure 2:**
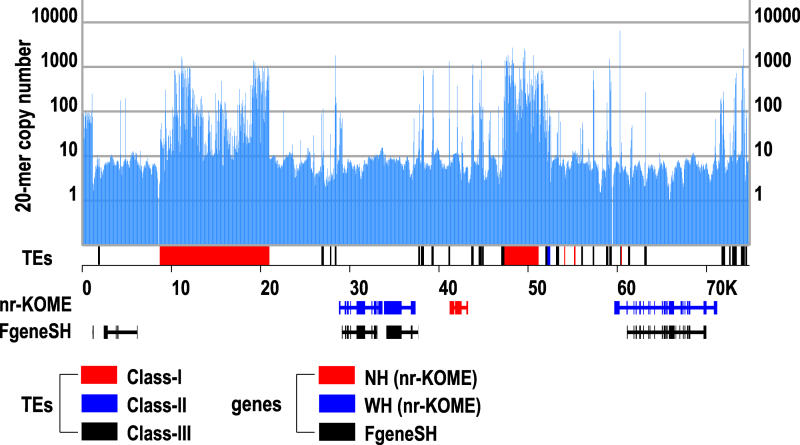
A Region on Beijing *indica* Chromosome 2, Showing Three Gene Islands Separated by Two Intergenic Repeat Clusters of High 20-mer Copy Number Transposable elements identified by RepeatMasker are classified based on the nomenclature of Table S2. Depicted genes include both nr-KOME cDNAs and FGENESH predictions.

For methodological consistency, we annotated all three rice assemblies using the same procedures. We use FGENESH [35] for gene prediction because it has been shown to be the best of the available ab initio algorithms for rice [1]. An updated performance assessment is shown in Figure S3. The challenge in removing erroneous predictions resulting from TEs lies in how we compensate for the fact that the database used by RepeatMasker is incomplete. Figure 2 demonstrates how grass genomes are organized as gene islands of low copy number separated by intergenic repeat clusters of high copy number. We set a dividing line at copy number 10, not because there are no TEs below it but because there are few genes above it. Specifically, for genes defined by nr-KOME, 99.4% of the exons and 98.1% of the introns are attributed to 20-mers of copy number under 10. Using the finished sequence of Chromosomes 1 and 10, we show in Figure S4 that the mean (median) sizes are 23.7 kb (9.6 kb) for gene islands and 5.6 kb (3.5 kb) for intergenic repeat clusters. Applying RepeatMasker to these intergenic repeat clusters only identifies 47.6% as TEs, overwhelmingly *gypsy* and *copia*. We therefore propose to filter the predictions by removing genes for which 50% of their coding region is attributable to any combination of RepeatMasker TEs or 20-mers of copy number over 10.

Although this filter might remove some real genes, it removes only a small fraction of them, as demonstrated by the nr-KOME cDNAs, where it eliminates 0.9% of these genes. In contrast, applying this same filter to the FGENESH predictions eliminates 19%–22% of the gene set, as indicated in Table 3. We believe that most of the removed predictions are TEs and that the benefits of removing these artifacts outweigh the risks of removing real genes. After this procedure, the gene counts range from 49,088 (Beijing *indica*) to 45,824 (Syngenta *japonica*) to 43,635 (IRGSP *japonica*). Previous estimates for Chromosomes 1, 4, and 10 made no such correction and found slightly larger numbers. About 45%–47% of predicted genes are NH, in contrast to 34.3% of nr-KOME cDNAs. This discrepancy is due to a combination of prediction errors and the fact that NH genes are difficult to clone because they are poorly expressed (data not shown). Radically different numbers have been given for mean gene size, from 2.6 kb in Chromosome 10 to 4.5 kb in our previous article. As we show in Table 4, much of this discrepancy can be explained by differences in definition. Predicted genes have a mean (median) size of 2.5 kb (1.8 kb). We get the same result for nr-KOME if we exclude UTRs, but we get a size of 3.6 kb (2.9 kb) if we include UTRs. If we restrict the genes to WH genes, this raises the gene size to 4.0 kb (3.4 kb).

**Table 3:**
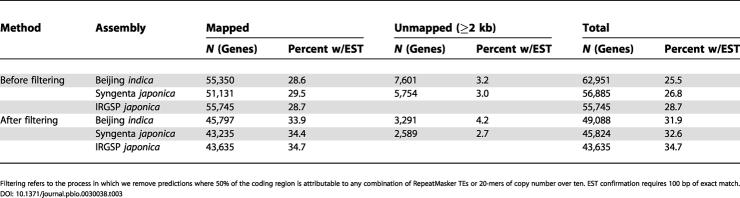
Number of FGENESH Predictions in All Three Rice Assemblies

Filtering refers to the process in which we remove predictions where 50% of the coding region is attributable to any combination of RepeatMasker TEs or 20-mers of copy number over ten. EST confirmation requires 100 bp of exact match

**Table 4:**
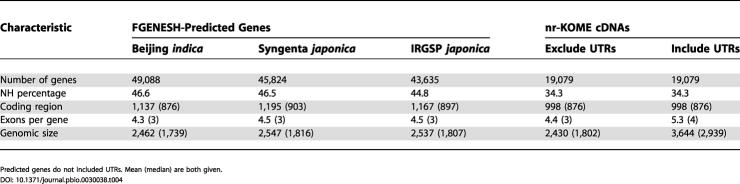
Characteristics of FGENESH Predictions and nr-KOME cDNAs

Predicted genes do not included UTRs. Mean (median) are both given

**Figure 3:**
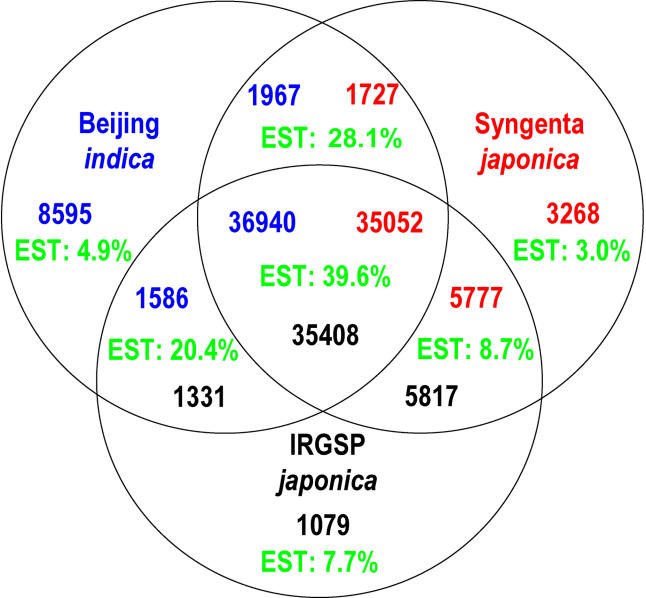
Overlapping FGENESH Predictions in All Three Rice Assemblies Two predictions are shared when 50% of their coding regions can be aligned. Because of imprecision in the predictions and overlap criteria, we get slightly different numbers for each assembly, and these are encoded through multiple color-coded numbers in the Venn diagram. EST confirmation requires 100 bp of exact match. Unlike the genes, we do not bother to show a different number for each assembly, because they are very similar.

Even after removing likely TEs, two particular subclasses warrant caution, as they contain a higher than normal rate of erroneous predictions, which is reflected in a reduced rate of confirmation by ESTs. Overall, we used 200,648 ESTs from *indica, japonica,* and other rice subspecies. The confirmation rule is exact match over 100 bp. Genes predicted in unmapped sequences are confirmed at much lower rates than genes predicted in mapped sequences—about 11 times lower, even after removing 3.4 times as many unmapped genes as likely TEs. Genes unique to only one assembly also show lower confirmation rates, by a factor of roughly nine, when compared with the 35,052–36,940 genes that are shared by all three assemblies, as summarized in Figure 3. A more detailed analysis is given in Table S3. What is important is that few of these genes are likely to be real. We can use the ratio of the EST confirmation rates to correct our gene count estimates. Beijing *indica* is computed as [(36,940 × 39.6) + (1967 × 28.1) + (1586 × 20.4) + (8595 × 4.9)]/39.6 = 40,216. Similarly, we get 37,794 for Syngenta *japonica* and 37,581 for IRGSP *japonica*. If unique genes are truly expressed at lower levels than shared genes, this procedure might underestimate the gene count. One should thus interpret these numbers as lower bounds.

Using the same EST adjustments, the number of predicted genes in Beijing *indica* that are not found in either *japonica* assembly is 1,064. Conversely, Syngenta *japonica* has 1,517 predicted genes that are not in *indica* (the number for IRGSP *japonica* is 1,479). As a fraction of the totals, 2.2% and 3.3% of *indica* and *japonica* genes, respectively, are unique to the subspecies, which is plausibly comparable to the amount of sequence that might still be missing. There is little difference in gene content between *indica* and *japonica,* but major differences are seen in the intergenic regions. Only 260 Mb (72%) of the mapped sequences can be aligned. This remains true no matter how much we relax the alignment parameters, and despite the fact that we had 34,190 “anchor points” (see Figure 1), which ensure that the *indica–japonica* comparisons are always made between the same regions of the chromosomes from the two subspecies. This unalignable fraction would be even larger if unmapped and unassembled sequences were included. Notice also that 20-mer repeat content is 59.2% in mapped-but-unaligned regions, as compared to 31.8% in mapped-and-aligned regions. Everything that we see is consistent with the fact that plant intergenic regions are rapidly evolving [36]. As further proof of this fact, Table 5 shows the SNP rates in these alignable regions. The rates vary from as little as 3.0 SNP/kb in coding regions to as much as 27.6 SNP/kb in identifiable TEs.

**Table 5:**
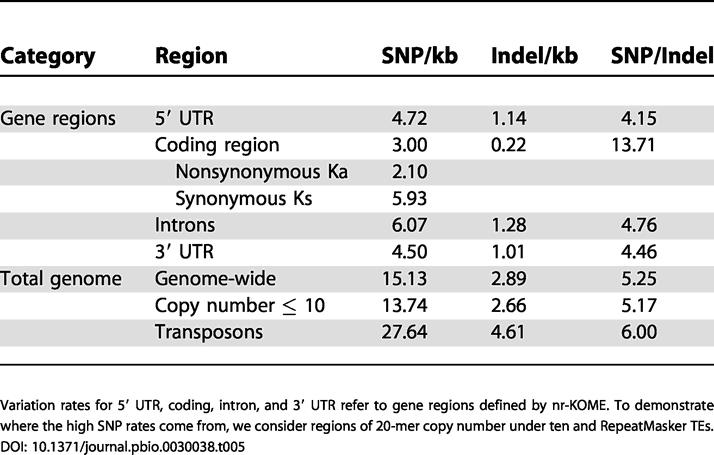
Variation between *indica* and *japonica* Defined by SNP and Insertion–Deletion (Indel) Rates

Variation rates for 5′ UTR, coding, intron, and 3′ UTR refer to gene regions defined by nr-KOME. To demonstrate where the high SNP rates come from, we consider regions of 20-mer copy number under ten and RepeatMasker TEs

**Figure 4:**
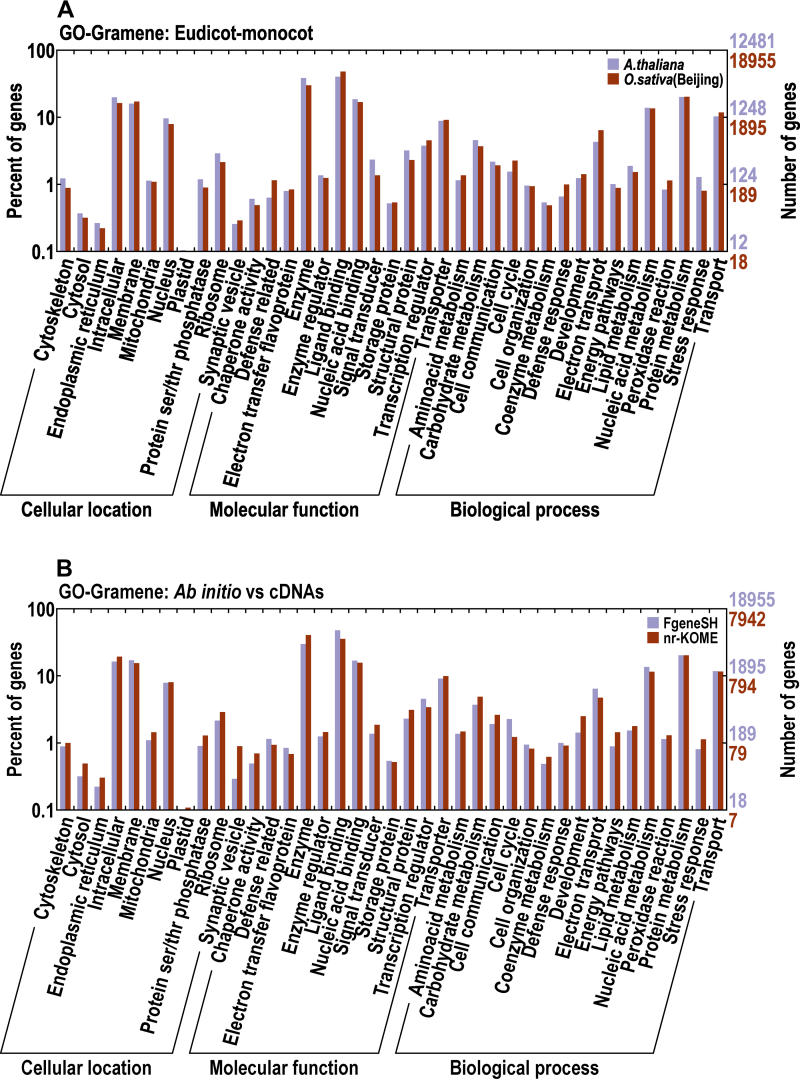
Functional Classifications from GO, Focused on Plant-Specific Categories Outlined by Gramene (A) compares predicted genes from *Arabidopsis* and Beijing *indica*. (B) compares predicted genes from Beijing *indica* with nr-KOME cDNAs. We ignore categories with less than 0.1% of the genes.

Biological functions are inferred by and displayed within the Bioverse framework [37,38] by combining more than seven of the latest computational techniques, including profile–profile comparison to well-curated protein families, motif discovery, and structural assignment/prediction. Note that we do not use transitive annotations, as their error propagation rates are too high. We present these results in Gene Ontology (GO) [39] and InterPro [40] formats. Functions are assigned to 60.2% of WH genes and even to 17.5% of NH genes, reflecting the fact that Bioverse uses highly sensitive techniques. Figure 4 shows a couple of our GO comparisons, focused on plant-specific categories in Gramene [41]. From the fraction of the gene set in each category, rice and *Arabidopsis* are remarkably similar. FGENESH-predicted genes and nr-KOME cDNAs exhibit very similar patterns too, confirming the unbiased nature of these cDNAs. InterPro domain categories tell much the same story, and these data are summarized in Table S4.

**Figure 5:**
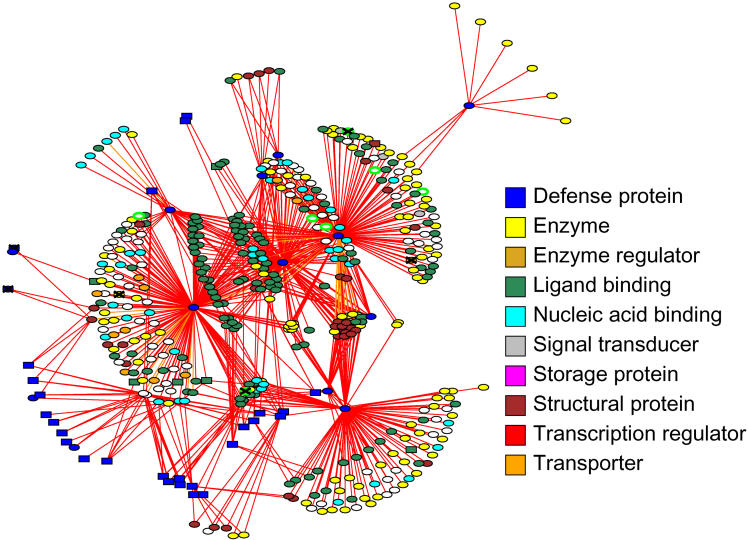
A Sample Bioverse-Predicted Interaction Network for Defense Proteins and Their Direct Neighbors The symbols are colored to indicate some of the major GO categories under “molecular function.” We draw a cross over the symbol for an NH gene. Rectangles indicate proteins that are manually classified as being R-genes. They appear on genes that are not colored as defense, because some genes have multiple functions, not because of an annotation error. The white circles with green outline are unannotated genes that might also belong to this network, at a lower confidence.

Bioverse is distinguished from other annotation pipelines in that it also determines protein–protein interactions. Two proteins are predicted to interact if they are both similar in sequence to proteins involved in known interactions. The known interactions are taken from numerous sources, including Protein Data Bank [42] and the Database of Interacting Proteins (which stores yeast two-hybrid studies, affinity column studies, and literature searches) [43]. The resultant network has 1,879 proteins/nodes with 8,902 unique interactions. Figure 5 highlights a small portion of this network, for defense proteins (i.e., classified as “defense related” under GO molecular function or “defense response” under GO biological process) and their direct neighbors in the network. Many occupy central positions, meaning the network would fall apart if they were removed. Such genes are essential for cell survival [44]. More details can be found at http://bioverse.compbio.washington.edu.

Figure S5 shows that, near the centromeres, there is an increase in TE density (especially for large class I TEs like *gypsy* and *copia*) and a decrease in gene density. A more detailed view is given by the pullout figures of Figure S6, right down to the level of individual genes and TEs, to emphasize the excellent level of concordance between the two different WGS assemblies: Beijing *indica* and Syngenta *japonica*.

### Evidence of Whole-Genome Duplication

Duplication of individual genes, chromosomal segments, or even entire genomes is an important source of raw materials for gene genesis [45]. In the extreme case of a whole-genome duplication (WGD), convincing examples are difficult to find because of the expected rapid loss of duplicated genes and because the rate of individual gene duplication is high enough to mask any remnants of an ancient WGD [46]. Yeast was the first genome in which a WGD was detected [47]. In plants, the existence issue is not disputed, as polyploidy is common [48,49,50,51,52,53], but even with complete genome sequence, many details remain obscure. For *Arabidopsis,* the number and timing of these duplication events is still unknown [54,55,56,57,58,59]. For rice, segmental duplications were known [60,61,62] before the rice genome sequence was published. However, detailed analysis of this sequence has resulted in the contradictory assertions that rice is an ancient aneuploid [14] and an ancient polyploid [15]. Here, we resolve this conflict by showing that every conceivable class of duplication that could have happened did in fact happen, including a WGD.

We accept that every class of duplication is present in the same genome, and we thus explicitly assign, to every homolog pair, a status as to the class of duplication from which it came. For the sake of discussion, we define three classes: segmental duplication of multiple genes along a chromosome, tandem duplication of individual genes, and a category called background duplications to encompass everything else that cannot be so easily classified. In this conception, a WGD is a collection of segmental duplications that cover a majority of the genome, all of which date back to a common time in evolutionary history. All three rice assemblies give the same result, so we show only Beijing *indica*.

Unlike previous analyses, we avoid predicted genes. Instead, we define a homolog pair to be a single nr-KOME cDNA and one of its potentially many homologs within rice. These homologs are defined by translating the cDNA's coding sequence into protein and searching the rice genome in all six reading frames for putative exons, with TBlastN at E-values of 10^−7^. Exons in the same order and orientation are linked together, and success is declared if these linked exons can account for 50% of the original protein sequence. This technique has the advantage that the homolog need not be a cDNA or a predicted gene (as neither dataset is likely to be complete). In fact, the homolog might even be a remnant of an ancient duplication that is no longer a functional gene. Complications are found at two extremes. Many cDNAs have no homologs, but many others have too many homologs. In particular, 24.5% of WH genes have no homologs in rice, whereas 64.4% of NH genes have no homologs in rice. Because NH genes are dispersed throughout the genome, sandwiched between WH genes, we cannot adopt a strict colinearity rule in our search for duplicated segments. There would be too many exceptions. Conversely, when there is at least one homolog in rice, the mean (median) number of homologs per cDNA is 40 (5). Rather than deal with the complexities of this situation, we focus first on the cDNAs with one and only one homolog. This reduces the background duplication noise and allows us to identify trend lines indicative of segmental and tandem gene duplications. We can then add back those cDNAs with more than one homolog that we had rejected earlier by using our newly defined trend lines to constrain the choices.

**Figure 6:**
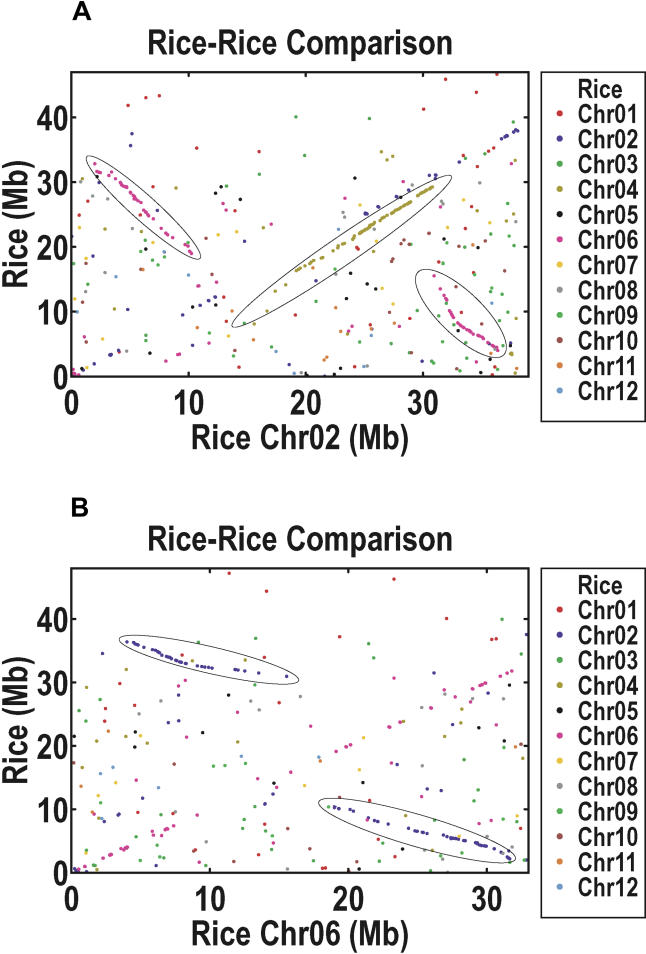
Duplicated Segments in the Beijing *indica* Assembly Depicted here are the plots for Chromosomes 2 (A) and 6 (B). Each data point represents the coordinated genomic positions in a homolog pair, consisting of one nr-KOME cDNA and its one and only TBlastN homolog in rice. Shown on the *x*-axis is the position of a gene on the indicated chromosome, and shown on the *y*-axis is the position of its homolog on any of the rice chromosomes, with chromosome number encoded by the colors indicated on the legend at the right.

**Figure 7:**
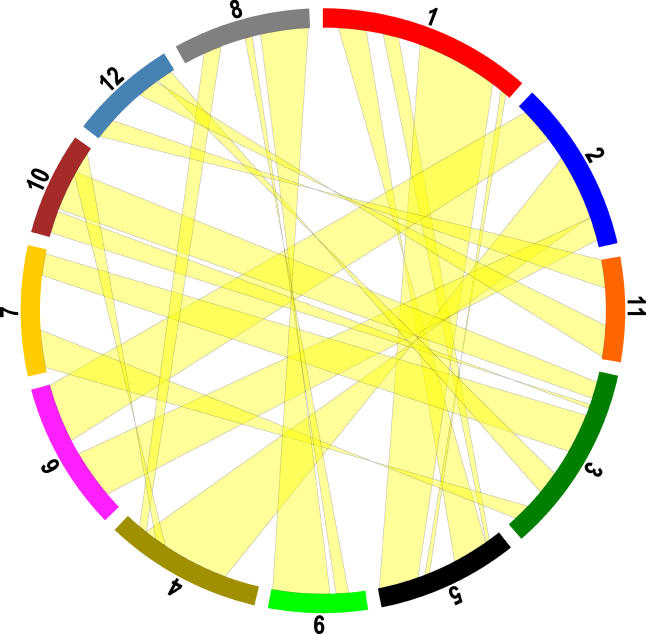
Graphical View of All Duplicated Segments The 12 chromosomes are depicted along the perimeter of a circle, not in order but slightly rearranged so as to untangle the connections between segments. Overall, we cover 65.7% of the genome.

The above procedure leaves us with 2,271 homolog pairs (or cDNAs). We adopt a graphical approach, because in the presence of massive background noise, trend lines are often easier to identify by eye than by software. Figure 6 depicts Chromosomes 2 and 6, and Figure S7 depicts all 12 chromosomes. There are 18 pairs of duplicated segments that together cover 65.7% of the length of all the mapped super-scaffolds. The mean (median) number of homolog pairs per segment is 34 (23). The segment sizes are 6.9 Mb (5.4 Mb), and they differ by 43% (42%) within a segment pair, which is not at all unexpected given the rapidly evolving nature of the rice intergenic regions. Instances of multiple duplicated segments on the same chromosomal region are extremely rare, covering only 0.9% of the total length. No additional multilevel duplications are detected if we use cDNAs with up to two homologs, as opposed to those with only one. Notice also that there are duplicated segments on all 12 rice chromosomes, as summarized in Figure 7.

**Figure 8:**
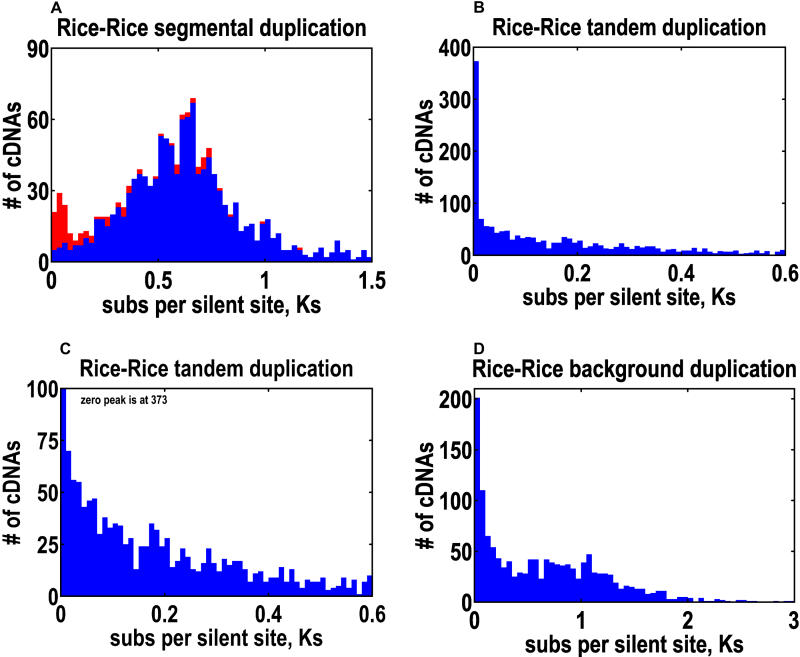
Distribution of Substitutions per Silent Site (Ks) for Homolog Pairs in Segmental, Tandem, and Background Duplications In (A), contributions from the recent segmental duplication on Chromosomes 11 and 12 are colored in red. The tandem duplication data are shown on two different scales, one to emphasize the magnitude of the zero peak (B) and another to highlight the exponential decay (C). Background duplications are shown in (D).

One can date the duplications by computing the number of substitutions per silent site (Ks). Multiple substitution corrections are done within K-Estimator [63]. To improve our statistics, we now include the higher-order homologs (those cDNAs with more than one homolog that we had removed before). Table 6 shows that this doubles or triples the number of homolog pairs in every segment and brings the mean (median) to 74 (53). The resultant Ks distribution is shown in Figure 8. One pair of segments on Chromosomes 11 and 12 is more recent in origin and has more homolog pairs per unit length than all the others. It was previously identified in many publications. If we ignore this segment pair, the mean Ks is 0.69, dating the duplication event to 53 million years ago (Mya), assuming a neutral evolutionary rate of 6.5 × 10^−9^ substitutions per silent site per year [64]. Most of the uncertainties are due to the multiple-substitution corrections for Ks. Another popular algorithm for Ks [65] dates the duplication event to 94 Mya.

**Table 6:**
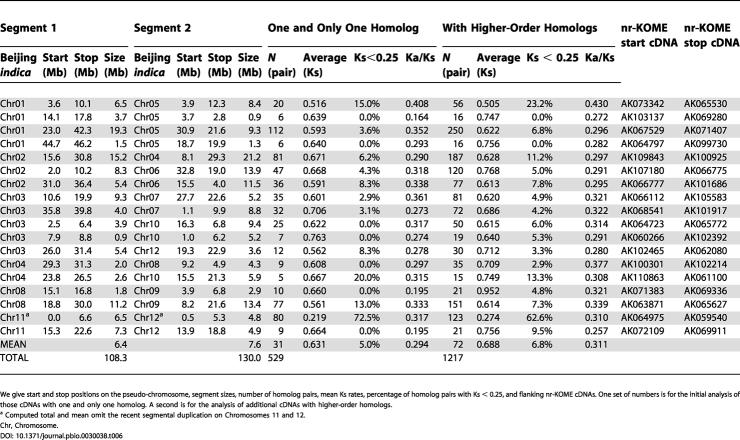
Summary of Duplicated Segments in the Beijing *indica* Assembly

We give start and stop positions on the pseudo-chromosome, segment sizes, number of homolog pairs, mean Ks rates, percentage of homolog pairs with Ks < 0.25, and flanking nr-KOME cDNAs. One set of numbers is for the initial analysis of those cDNAs with one and only one homolog. A second is for the analysis of additional cDNAs with higher-order homologs

^a^ Computed total and mean omit the recent segmental duplication on Chromosomes 11 and 12

Chr, Chromosome

The molecular clock can also vary between genes and between taxa [66,67]. Evidence for the former is seen in the width of the distribution for Ks in Figure 8, which has a standard deviation of 49.8% based on individual homolog pairs (as opposed to 14.5% when based on duplicated segment pairs). We believe that the variation between genes will cancel out, but we cannot remove the systematic error resulting from the multiple substitution corrections or the potential error in the 6.5 × 10^−9^ evolutionary rate (which was derived from a small number of genes). However, all we really want to know is whether the duplication event occurred before or after the origin of the grasses, 55–70 Mya [68]. To this end, phylogenetic approaches can be used, albeit for a limited number of genes, because so few plants have been fully sequenced. A majority of these phylogenies indicate that the duplication event occurred before this pivotal point in evolution [14]. Almost certainly, the duplication event occurred after the divergence of monocots and eudicots, 170–235 Mya [69]. However, the best evidence for the statement that the duplication event must have predated the origin of the grasses is the fact that there is no other way to reconcile it with the widely observed synteny between different grass genomes [70]. In striking contrast, the Chromosome 11 to 12 duplication dates back to just 21 Mya, which postdates the origins of the grasses by a comfortable margin.

If we accept that a WGD occurred before the divergence of maize–rice, and that a duplication in Chromosomes 11 and 12 occurred afterward, we might then expect to find two levels of duplication in this region of rice. We thus extended our analysis to consider cDNAs that map to as many as four loci. No indications of such a multilevel duplication could be found. Undaunted, we decided to try another approach and analyzed the maize–rice synteny, starting from the maize genetic map [71]. The results are given in Figures S8 and S9. We found 35 pairs of syntenic segments covering 71.4% and 52.9% of the maize and rice genomes, respectively. All previously identified segments are confirmed, except for those on Chromosomes 11 and 12 of rice. No synteny is found in the vicinity of this recent duplication. There are many explanations, and they need not contradict our hypothesis, as only 65.7% of the rice genome is in identifiably duplicated segments, and the region from Chromosome 11 to 12 is a minuscule 3.0% of the genome. It is possible that any traces of the WGD had already been lost by the time this recent duplication occurred. The region is also sufficiently small that any synteny with maize would be difficult to detect. It is too early to draw conclusions, especially as maize–rice synteny appears to be much more complicated than previously thought [72].

Given how so much of the rice genome is covered by segmental duplications, and the fact that all but one of our 18 segment pairs date back to the same time, give or take a standard deviation of 14.5%, the simplest interpretation is that a WGD did occur and that it happened before the origin of the grasses. However, it is equally clear that other classes of duplications are also present, and these are worth investigating too.

### Ongoing Individual Gene Duplications

Tandem duplications are represented by the trend along the diagonal, Y = X, that is observed in all chromosomes (see Figures 6 and Figure S7). Segmental duplications within the same chromosome are possible, but their trend would not be along the diagonal, and none were actually seen in our analysis. As an indicator of the prevalence of the three different duplication classes, we use the number of homolog pairs before and after the inclusion of higher-order homologs. Segmental duplications contain 609 and 1,340 pairs, whereas tandem duplications contain 311 and 957 pairs. We can increase the tandem numbers by relaxing our definitions to allow two TBlastN homologs of an nr-KOME cDNA to count as a homolog pair (instead of insisting that one always be a cDNA). This is what we use in the Ks distribution plot of Figure 8, which contains 1,696 homolog pairs. Rather than a maximum in the distribution at some nonzero Ks, we find a big peak at zero Ks, followed afterward by an exponential decay. The implication is that tandem duplication is an ongoing evolutionary process that provides an endless source of raw materials for gene genesis. If we adopt the methods and parameters of the *Arabidopsis* genome paper, we find that 16.5% of the rice genome is tandemly duplicated, compared to 16.2% of the *Arabidopsis* genome. Note, however, that the Ks distribution for tandemly duplicated genes in *Arabidopsis* is highly unusual, in the sense that it does not exhibit the big peak at zero Ks that is seen in virtually every other plant genome [52].

**Figure 9:**
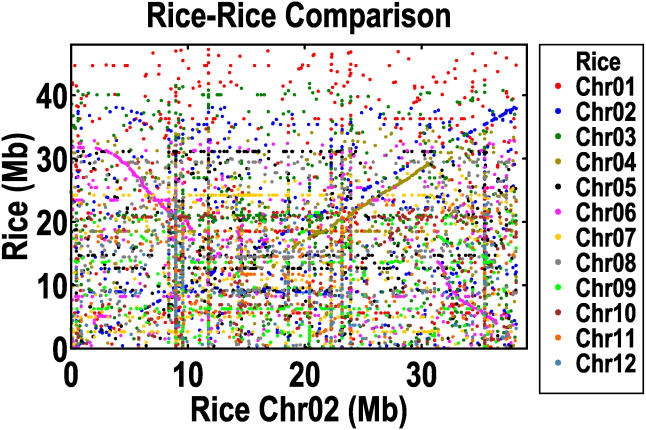
A View of All Duplications Found on Rice Chromosome 2 In contrast to Figure 6, where we featured those cDNAs with one and only one TBlastN homolog, here we show all detectable TBlastN homologs, up to a maximum of 1,000 per cDNA.

In addition to segmental and tandem duplications, there is a third and last class of duplications that looks like background noise in our figures. The number of homolog pairs is 1,351 and 32,384 before and after higher-order homologs, respectively, although with no trend line to constrain the choice of homologs, that second number is almost certainly an overestimate, since only 4,212 cDNAs are involved. Surprisingly few of these higher-order homologs are the result of processed pseudogenes, as the number of cases in which a multiexon cDNA pairs with a single-exon TBlastN homolog is 9.8%. To demonstrate how overwhelmingly these higher-order homologs contribute to the background noise, Figure 9 depicts what Chromosome 2 would have looked like if we had included them. For simplicity of interpretation, Figure 8 is the Ks distribution of the cDNAs with one and only one homolog. This distribution has characteristics of the distribution for tandem duplications—large peak at zero Ks followed by exponential decay—except that the magnitudes of the Ks are much larger for background duplications. We believe that most of these background duplications were originally tandem duplications that, over time, migrated to other parts of the genome, but we cannot rule out the possibility of direct duplications to remote loci. Some older duplications may even be due to migration of genes from segmental duplications, but these are a small part of the overall picture. However we do the counting, it appears that this combination of recent tandem and background duplications, which we call individual gene duplications, would rival any contribution from the segmental duplications.

**Figure 10:**
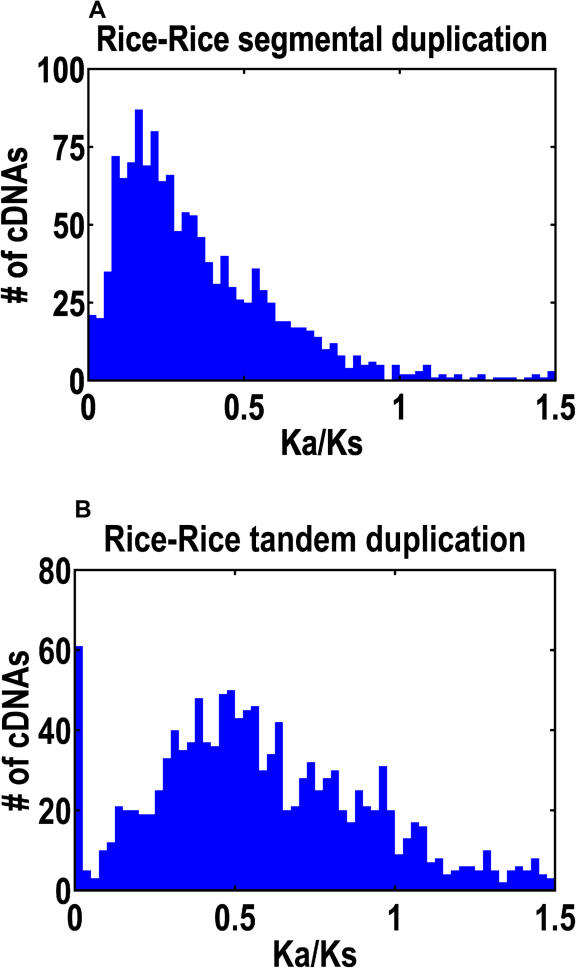
Ka/Ks Distribution for Homolog Pairs Ka and Ks are the fraction of the available nonsynonymous and synonymous sites that are changed in the homolog pairs. Ka/Ks > 1 is an indicator of positive selection. Shown is the Ka/Ks distribution for segmental duplications (A) and for tandem duplications (B).

Tandem and segmental duplications show markedly different Ka/Ks distributions, a popular test for evolutionary selection, where Ka and Ks refer to the fraction of nonsynonymous and synonymous sites, respectively, that are changed within a homolog pair [73]. Ka/Ks is one under neutrality, below one under purifying selection, and above one under adaptive selection. Tandem duplications tend to have larger Ka/Ks values, as we show in Figure 10. The averages are 0.720 (tandem) and 0.365 (segmental), and more homolog pairs exhibit Ka/Ks > 1 in tandem duplications. This is consistent with the observation that more recent duplications tend to have larger Ka/Ks values [74] and with the idea that, immediately after duplication, one of the two genes undergoes a fast evolving phase [75]. Finally, let us consider again those nr-KOME cDNAs with one and only one homolog. Among the ones assigned to a tandem duplication, 65.3% are NH, but among the ones assigned to a segmental duplication, 23.8% are NH. Hence, there is a marked correlation between NH genes and tandem duplications.

Our WGD is in good agreement with the results of Paterson et al. [15], but we can also explain the seemingly contradictory results of Vandepoele et al. [14] First, they did not have a complete genome; about two-thirds of their segmental duplications were interrupted by a break in the assembly. Second, their algorithms were very likely confounded by the many NH genes with no homologs in rice itself and by the many individual gene duplications that in aggregate masked the WGD. In fact, their segmental duplications had a Ks distribution similar to ours, but they only covered 15% of the genome. Then, when they examined the distribution of Ks for all duplicates, what they found was a big peak at zero Ks. This lead them to conclude there was no WGD, when, in fact, almost every class of duplication that had been hypothesized was present, and they needed only to allow for that.

## Discussion

Until recently, *Arabidopsis* was the only sequenced plant genome. When two rice genomes were first published in draft format, the comparative analyses that could be done were hindered by a lack of long-range contiguity. Now, there are three plant genomes *(indica* rice, *japonica* rice, and *Arabidopsis)* with multimegabase contiguity. In our analyses, we strived to maintain methodological consistency. To assess the accuracy of our assemblies, we first compared IRGSP *japonica* to Syngenta *japonica,* so that polymorphic differences would not be a confounding factor. To compare gene content in the three rice assemblies, we annotated them all with the same procedures. Our conclusion is that, even if the WGS method does fall just slightly short of the clone-by-clone method in terms of accuracy and completeness, it comes remarkably close. This is why all the genome-sequencing projects now being funded by the National Human Genome Research Institute (in the United States) are being done with WGS methods (http://www.genome.gov/11007951). Rice is also now one of the few organisms with the luxury of having a complete genome sequence for two important subspecies. Comparisons of *indica* and *japonica* reveal strikingly little difference in the gene content, but there are massive intergenic differences. This vindicates our strategy to focus on genic sequences, because if the intergenic sequences are so unstable even between *indica* and *japonica,* they are highly unlikely to be functional.

Our analysis of the duplication history in rice resolves a simmering dispute and, at the same time, raises some intriguing questions. We find evidence for an ancient WGD, a recent segmental duplication, and massive ongoing individual gene duplications. This last phenomenon can explain certain unexpected findings. Sequencing of orthologous loci between grass genomes has identified many smaller-scale rearrangements that were not seen in the original map-based studies. Many of these exceptions to synteny are due to tandem duplications [76,77,78], which makes sense, given how these duplications are a frequent and ongoing event for grass genome evolution. In addition, the massive ongoing individual gene duplications provide a never-ending source of raw material for gene genesis. We believe that the large number of rice NH genes is a transient effect of this ongoing process. The contrary argument is that any such transients cannot be long-lived, as one of the two genes must decay rapidly to avoid the dosage-doubling problem [79,80]. We believe this is irrelevant when there is a continual injection of new gene duplicates. Additional details must, however, be deferred to a future article, in which we can better address other important issues, such as the critical need to confirm NH genes in proteomics and conservation in the maize genome sequence.

Looking toward the future, we would point out that the Chinese Superhybrid Rice Genome Project was designed to include not only a major subspecies of rice, namely, the *indica* variety represented by*93–11,* but also the maternal strain of the *LYP9* superhybrid, *PA64s,* which has a complex breeding history incorporating genetic material from *indica, japonica,* and *javanica*—all of the major subspecies of cultivated rice. Work on *PA64s* is continuing at our Beijing center. For the research community, we will be providing DNA microarrays to facilitate the systematic studies of gene expression in different tissues and developmental stages, and under different physiological and environmental conditions. We will develop molecular markers for mapping causative genes in mutant lines and marker-assisted breeding. This publication, and the associated data release, is also a fitting way to celebrate the end of 2004, which the General Assembly of the United Nations declared to be the International Year of Rice (http://www.fao.org/rice2004).

## Materials and Methods

### 

#### Construction of reference cDNAs: nr-KOME

The initial Knowledge-Based Oryza Molecular-Biological Encyclopedia dataset [25] had 28,444 *japonica* cDNAs with complete open reading frames. These cDNAs were aligned to Syngenta *japonica,* and when two alignments overlapped by at least 100 bp, the smaller cDNA was removed. A small number of clones could not be aligned—not even partially—to any of our three rice assemblies (Beijing *indica,* Syngenta *japonica,* and IRGSP *japonica*). Removing these as nonrice contaminants gave a set of 19,079 nonredundant cDNAs that we call nr-KOME. Because the sequence quality is so high, we could use the longest open reading frame for the overwhelming majority of these cDNAs, without having to correct for sequencing errors. Minor corrections are applied to 2.5% of these cDNAs, following the methods first developed for GenScan [81].

#### Repeats and their effects on WGS misassembly

The basic procedure for converting sequence reads into contigs and scaffolds was described in our original publication on RePS [26], our WGS assembler. A common source of confusion is the distinction between mathematically defined repeats (MDRs) and biologically defined repeats. What we focus on are MDRs, which refer to 20-mer sequences that are exactly repeated in the genome, without regard to their underlying biological context. In our nomenclature, “depth” refers to the number of times that a 20-mer appears in the unassembled sequence reads and “copy number” refers to the number of times that it appears in the (correctly assembled) genome. “Coverage” is the number of times that the genome is redundantly sampled, and therefore depth = copy number × coverage. Special procedures are used to compute depths efficiently [27].

In a WGS assembly, the problems arise from the MDRs, which are not equivalent to the biologically defined repeats. For example, TEs qualify as biologically defined repeats, and they can be recognized, even after many millions of years of degradation, by specialized programs like RepeatMasker (http://www.repeatmasker.org). However, the degradation makes it trivial to distinguish between two copies of an ancient TE, so these do not cause assembly problems. It is also relatively easy to distinguish between gene duplicates, because their introns and flanking intergenic regions are under fewer evolutionary constraints than their exons. Even for recent TEs and gene duplicates, assembly problems can be avoided, because RePS computes the copy number for every 20-mer in the WGS assembly, and it will refuse to join anything that might be ambiguous. Indeed, the only way a misassembly can occur is if there is a low copy MDR and its copy number is underestimated by RePS. All of our tests show that, although this can happen, it is a rare event.

#### On the usefulness (or not) of BAC end pairs

The fundamental challenge was that we had to create super-scaffolds of megabase size from scaffolds of 30-kb size. It is generally thought that BAC end pairs are useful for this purpose, but this is not true when the BAC inserts, typically 122–187 kb, are much bigger than the scaffold sizes. Instead of linking adjacent scaffolds, they link every fourth to sixth scaffold. The fact that the density of BAC ends is 2.3 kb does not help, because there is no way to determine the order and orientation of the overlapping BACs. Fingerprint maps do provide some ordering information, but nothing like 2.3-kb resolution, and orientation information is still missing. The danger in using the BACs at this point is that you end up with a morass of interleaving super-scaffolds [26], with no way to untangle them. We actually did an assembly with only the BACs, and the result was that the super-scaffolds were 87% larger than they should have been. In the mouse project [82], the solution was to use fosmid end pairs, because these inserts are constrained to an almost ideal size of 40 kb. In the case of rice, we did not need to sequence fosmid end pairs, because by combining the *indica* and *japonica* WGS assemblies, it is possible to get linking information at the requisite length scales. We did of course use all available BAC end pairs [83] (http://rgp.dna.affrc.go.jp/blast/runblast.html, but they were only useful after the intermediate-range linking that came from combining WGS assemblies.

#### Misassemblies versus polymorphic differences

To verify our WGS assemblies on the smaller-length scales that are more characteristic of genes, we compare them with IRGSP *japonica,* taking the latter as the “gold standard” not because it is perfect but because it more likely to be correct. We focus on gene regions by aligning nr-KOME cDNAs to IRGSP *japonica* and excising the sequences from the 5′ to 3′ UTRs, including introns and an additional 500 bp at both ends. What we search for are potential misassemblies due to misplaced reads. Given that a typical read is 500 bp, these should appear as segments of 500 bp or more in which the excised gene sequence cannot be aligned with the WGS assembly. Such discrepancies are noted based on where they occur in the context of the gene. Although it is possible to detect more than one discrepancy per gene, we only count the most serious discrepancy in each gene based on the likelihood of it being functional. The prioritization is from coding exon, to UTR exon, to intron. Notice that discrepancies of this nature are not always from misassemblies. In the Beijing *indica* comparison, they can also be due to polymorphic differences. Although there is no way to tell what any particular discrepancy is, we know the misassembly rate from the Syngenta *japonica* comparison. Therefore, any increase in the discrepancy rate in the Beijing *indica* comparison can be attributed to polymorphic differences.

#### Ab initio predictions in WH versus NH genes

FGENESH [35] behaves very differently for WH and NH genes, as defined by nr-KOME. Following the methods of our recent review [84], we compute false positive (FP) and false negative (FN) rates. Error rates are given on a per amino acid basis. This means that in addition to correctly identifying the coding bases, we require the reading frame to be correctly determined. WH genes show very low error rates (FP = 0.10 and FN = 0.05). Although NH genes show higher error rates (FP = 0.35 and FN = 0.25), these are not that much worse than human genes (FP = 0.30 and FN = 0.12), and like it or not, error rates like these are the state of the art in ab initio prediction. On closer examination, it is clear that most of the problems in rice are caused by single-exon genes with small coding regions, which are more prevalent among NH genes and form a category that all ab initio algorithms handle poorly. This category of genes does not affect the gene count because FP and FN cancel each other out. We therefore focus on removing TEs that are mistakenly called genes.

#### Comparison of *indica*-*japonica* to identify SNPs

The sequence alignments for *indica* and *japonica* are straightforward, with almost no chance of paralog confusion, because of our 34,190 unique “anchor points” (see Figure 1). We partition the sequence into four nonoverlapping categories called unassembled, assembled-but-unmapped, mapped-but-unaligned, and aligned. The last category is where almost all of the genes are, and where we can get polymorphism data. Detailed sequence alignments are computed with CrossMatch, a Smith-Waterman algorithm that is included in Phrap (http://www.phrap.org). This is preferred to any of the BLAST alignment tools, which, although they are faster, occasionally miss subtle details. To discriminate between polymorphisms and sequencing errors, we use the error probability *p* attached to every base, and given as *Q* = −10 × log(*p*). Following the rules established in the early days of large-scale polymorphism discovery [85], we use thresholds of *Q* > 23 at the SNP site and *Q* > 15 for the two flanking 5-bp regions. Experience has taught us that higher thresholds (30 and 22, respectively) are required for the indels. For comparison, an independent analysis [86] reported mean rates of 7.1 SNP/kb and 2.0 indel/kb, with 98% of these SNPs experimentally confirmed. Our SNP rates are two times higher because we aligned more of the intergenic sequence. If we eliminate this factor, say, by restricting our rates to the introns of the genic regions defined by nr-KOME, our rates are 6.1 SNP/kb and 1.3 indel/kb, which are actually lower than the rates from that independent analysis.

#### On the reliability of the *p*–*p* interaction data

Bioverse annotations in this article are dated July 2003 (FGENESH) and November 2002 (nr-KOME). Two proteins are said to interact if they are similar to two other proteins that are known to interact. Our criterion is that the product of the similarity measures (percentage identity) must exceed 0.15. For example, two proteins with 45% and 30% identity to two other proteins that are experimentally determined to interact would be rejected, as their score is 0.45 × 0.30 = 0.135. The reliability of this approach, especially for transfer of interaction data between organisms, has been demonstrated in *Saccharomyces cerevisiae, Caenorhabditis elegans, Drosophila melanogaster,* and Helicobacter pylori analyses [87]. As an example of a predicted interaction for rice that has been independently confirmed, Bioverse identification numbers 21736 and 8526 (score 0.21) show an interaction between CDK-activating kinase and H-type cyclins [88]. A general way to verify the predicted interactions is to compare them against known protein complexes in the Protein Data Bank. Unfortunately, there are few Protein Data Bank structures from rice, and even fewer are of protein complexes. Given this dearth of experimentally determined interactions for rice, Bioverse is almost the only source of large-scale interaction data.

#### Details of the duplication and synteny analysis

We defined a homolog pair as a single nr-KOME cDNA and its TBlastN homolog, but occasionally that TBlastN homolog will overlap with another cDNA. To avoid double counting, we keep only the larger of these two cDNAs. Segmental duplications identified by visual inspection must have at least five homolog pairs, with no more than 5 Mb between adjacent homolog pairs. We approximate the trend line with a second- or third-order polynomial, and to capture what our eyes indicate should be captured, we accept homolog pairs within a 500-kb radius of this polynomial. Slightly different definitions are used for tandem duplications, depending on application. For Ks, we allow two TBlastN homologs to count as a homolog pair and accept homolog pairs within a 50-kb radius of the diagonal, although the mean (median) center-to-center distance is 6.8 kb (4.7 kb). To compare tandem duplications in rice and *Arabidopsis,* we use the methods described in the *Arabidopsis* genome paper and analyze predicted genes with BlastP at E-values of 10^−20^.

To determine the maize–rice synteny, we began with 1,063 maize genetic markers [71] and searched for BlastN alignments to rice of at least 100-bp size and 80% identity. Given the segmental allotetraploid origins of maize [89], many markers are associated with two loci in maize. Each marker aligns to a mean (median) of 1.9 (1) loci in rice. We used only the longest of these alignments and verified in retrospect that using all of them would not have mattered. In the end, there are 35 pairs of syntenic segments, which cover 71.4% and 52.9% of the maize and rice genomes, respectively, and the mean (median) number of markers per syntenic segment is 18 (12).

## Supporting Information

Figure S1 Genetic Versus Physical Map Distance for All 12 Rice Chromosomes, Based on Beijing *indica*
Similar results are seen with the other two assemblies, Syngenta *japonica* and IRGSP *japonica*.
(1 MB EPS).

Figure S2 Number of Discrepant Markers in Comparisons of Genetic and Physical Maps for 1,519 Markers Found in All Three Rice Assemblies We count discrepancies where the markers are found (A) on different chromosomes and (B) in different locations on the same chromosome.
(458 KB ZIP).

Figure S3 Gene Prediction by FGENESH, Tested against nr-KOME cDNAs Genomic size refers to the unspliced transcript, with introns, but constrained to the region from the start to stop codons. CDS size refers to the spliced transcript, without introns. Predictions are assessed with FP and FN rates, where per-aa (per amino acid) refers to the fact that we check whether the reading frame is correct.
(351 KB ZIP).

Figure S4 Distribution of Sizes for Gene Islands and Intergenic Repeat Clusters, Based on Complete Sequence of Chromosomes 1 and 10 from IRGSP *japonica*
Intergenic repeat clusters are regions of size larger than 1.5 kb (i.e., between a MITE and a *gypsy*/*copia* TE), where most of the 20-mer copy numbers exceed ten. Lower copy number regions are tolerated up to a “maximum gap size,” which defaults to 150 bp. Regions lying between two adjacent intergenic repeat clusters are taken to be gene islands.
(233 KB ZIP).

Figure S5 Gene and TE Densities for Beijing *indica* Chromosome 7, as a Percentage of Sequence Length Near the centromeres, there is an increase in TE density (especially for the large, class I TEs such as *gypsy* and *copia*) and a decrease in gene density. This is not an artifact of the fact that WGS assemblies underrepresent larger TEs, as much the same effect is observed when we use IRGSP *japonica* instead (data not shown).
(362 KB ZIP).

Figure S6 Coordinated Annotation of the Individual Chromosomes for Beijing *indica* and Syngenta *japonica*
We depict all the genetic markers, nr-KOME cDNAs, FGENESH gene predictions, and transposable elements identified by RepeatMasker. Genes are depicted as WH (colored blue) or NH (colored red) based on their similarity to *Arabidopsis*. TEs are decomposed into classes I, II, and III. Correspondence between *indica* and *japonica* is indicated by drawing a connecting line between the 5′ ends of the nr-KOME cDNAs that clearly align to both assemblies.
(9.6 MB ZIP).

Figure S7 Duplicated Segments in the Beijing *indica* Assembly for All 12 Chromosomes, Plotted in the Manner of Figure 6, and with a Total of 12 Panels (507 KB ZIP).

Figure S8 Complete Synteny between Maize and Rice I Each point indicates the genomic positions for a maize genetic marker and its highest confidence match in rice. The *x*-axis shows a specific chromosome for one genome, and the *y*-axis shows all chromosomes for a second genome, with the chromosome numbers color-coded as per the legend. We show here 12 panels for rice.
(311 KB ZIP).

Figure S9 Complete Synteny between Maize and Rice II Each point indicates the genomic positions for a maize genetic marker and its highest confidence match in rice. The *x*-axis shows a specific chromosome for one genome, and the *y*-axis shows all chromosomes for a second genome, with the chromosome numbers color-coded as per the legend. We show here ten panels for maize.
(288 KB ZIP).

Table S1 Raw Data for Beijing *indica* and Syngenta *japonica* Assemblies Read length is the number of Q20 bases with an error rate of 10^−2^ or better. Effective coverage is based on the depth of reads in contigs over 5 kb in size, ignoring regions with 20-mer repeats. Clone insert sizes are specified in terms of tenth and 90th percentiles.
(16 KB XLS).

Table S2 Transposable Elements Identified with RepeatMasker Are Put into Classes I, II, and III As a result of our efforts to identify *indica*–*japonica* polymorphisms, the sequence is divided into four nonoverlapping categories: unassembled, assembled-but-unmapped, mapped-but-unaligned, and aligned (with all the SNPs).
(28 KB XLS).

Table S3 Detailed Analysis of Gene Overlaps from Figure 3
For each region of the Venn diagram, we use BLAT to align the predicted gene to the other assembly (or assemblies) where the gene is supposedly missing. The objective is to determine whether it is the sequence that is missing, or whether the discrepancy is due to the errors in the ab initio predictions. What we find is a bit of both. However, fragmented sequence assemblies are not a problem. If the gene is found at all, it is usually found in one piece. What is striking is that predicted genes that are unique to the two WGS assemblies do tend to be genuinely missing from IRGSP *japonica* sequence. This supports the idea that the WGS method can sometimes identify genes that are not well represented in the BAC clone libraries.
(17 KB XLS).

Table S4 Table of InterPro Domain Rankings One table compares predicted genes from *Arabidopsis* and Beijing *indica*. The second table compares predicted genes from Beijing *indica* with nr-KOME cDNAs.
(169 KB XLS).

### Accession Numbers

The DNA Data Bank of Japan/European Molecular Biology Laboratory/GenBank (BGI-RIS http://rise.genomics.org.cn [16]) project accession numbers for the WGS sequences discussed in this article are Beijing *indica* ( AAAA00000000, version AAAA02000000) and Syngenta *japonica* (AACV00000000, version AACV01000000).

### Note Added in Proof

The idea that TEs are often mistakenly annotated as genes was also suggested in a recent paper by Bennetzen et al. [90].

## Abbreviations

BAC: bacterial artificial chromosome
FN: false negative
FP: false positive
GO: Gene Ontology
IRGSP: International Rice Genome Sequencing Project
MDR: mathematically defined repeat
Mya: million years ago
NH: no homolog in *Arabidopsis*
nr-KOME: dataset of 19,079 nonredundant cDNAs from Knowledge-Based Oryza Molecular-Biological Encyclopedia
SNP: single nucleotide polymorphism
TE: transposable element
WGD: whole-genome duplication
WGS: whole-genome shotgun
WH: with homolog in *Arabidopsis*
