# Supplementary figures and images for: The Genomes of Oryza sativa: A History of Duplications
Source: PLoS Biol. 2005 Feb 1;3(2):e38. doi: 10.1371/journal.pbio.0030038 (PMC546038; doi:10.1371/journal.pbio.0030038)

Chr04

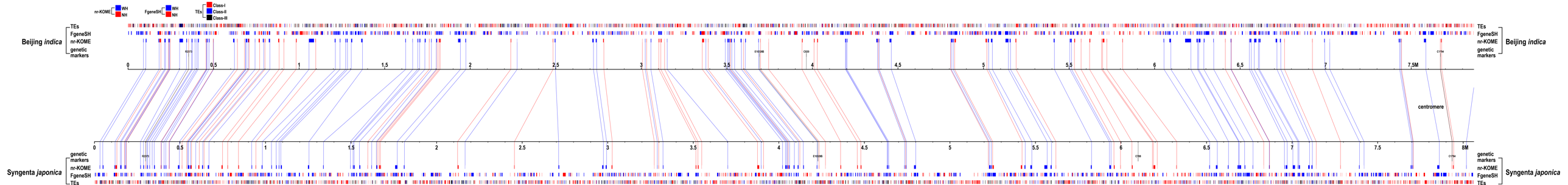

Supplement: Figure S6 — We depict all the genetic markers, nr-KOME cDNAs, FGENESH gene predictions, and transposable elements identified by RepeatMasker. Genes are depicted as WH (colored blue) or NH (colored red) based on their similarity to Arabidopsis. TEs are decomposed into classes I, II, and III. Correspondence between indica and japonica is indicated by drawing a connecting line between the 5′ ends of the nr-KOME cDNAs that clearly align to both assemblies. (9.6 MB ZIP). [file pbio.0030038.sg006.zip › two_piece/Chr04_L.pdf]

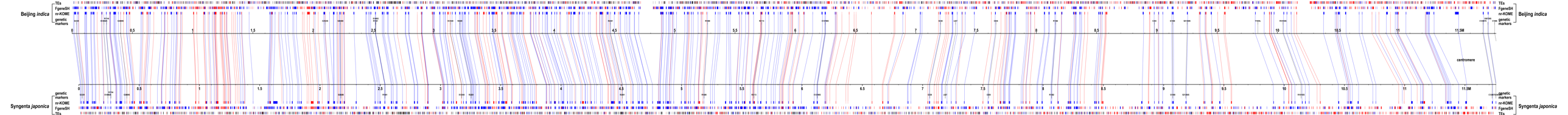

*Beijing indica*

*Beijing indica*

*Syngenta japonica*

*Syngenta japonica*

Supplement: Figure S6 — We depict all the genetic markers, nr-KOME cDNAs, FGENESH gene predictions, and transposable elements identified by RepeatMasker. Genes are depicted as WH (colored blue) or NH (colored red) based on their similarity to Arabidopsis. TEs are decomposed into classes I, II, and III. Correspondence between indica and japonica is indicated by drawing a connecting line between the 5′ ends of the nr-KOME cDNAs that clearly align to both assemblies. (9.6 MB ZIP). [file pbio.0030038.sg006.zip › two_piece/Chr07_L.pdf]

Chr09

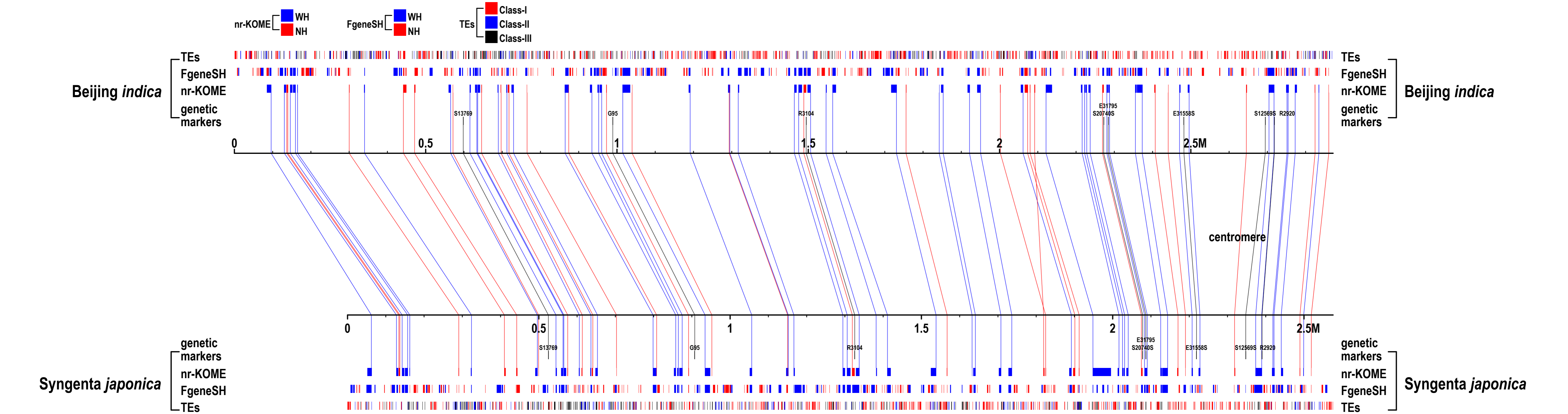

Supplement: Figure S6 — We depict all the genetic markers, nr-KOME cDNAs, FGENESH gene predictions, and transposable elements identified by RepeatMasker. Genes are depicted as WH (colored blue) or NH (colored red) based on their similarity to Arabidopsis. TEs are decomposed into classes I, II, and III. Correspondence between indica and japonica is indicated by drawing a connecting line between the 5′ ends of the nr-KOME cDNAs that clearly align to both assemblies. (9.6 MB ZIP). [file pbio.0030038.sg006.zip › two_piece/Chr09_L.pdf]

Chr10

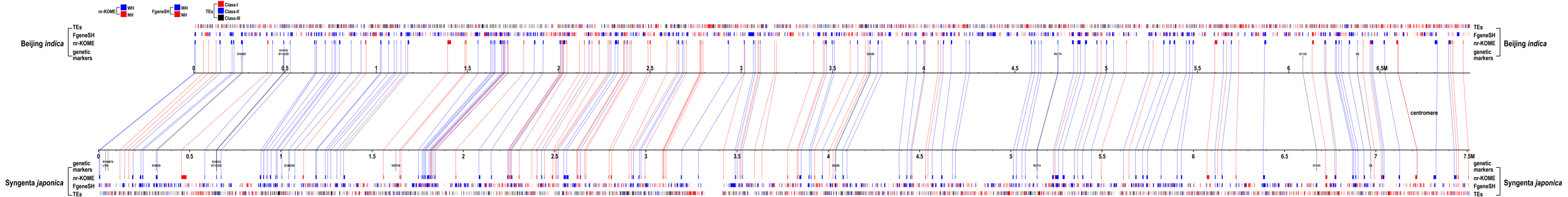

Supplement: Figure S6 — We depict all the genetic markers, nr-KOME cDNAs, FGENESH gene predictions, and transposable elements identified by RepeatMasker. Genes are depicted as WH (colored blue) or NH (colored red) based on their similarity to Arabidopsis. TEs are decomposed into classes I, II, and III. Correspondence between indica and japonica is indicated by drawing a connecting line between the 5′ ends of the nr-KOME cDNAs that clearly align to both assemblies. (9.6 MB ZIP). [file pbio.0030038.sg006.zip › two_piece/Chr10_L.pdf]

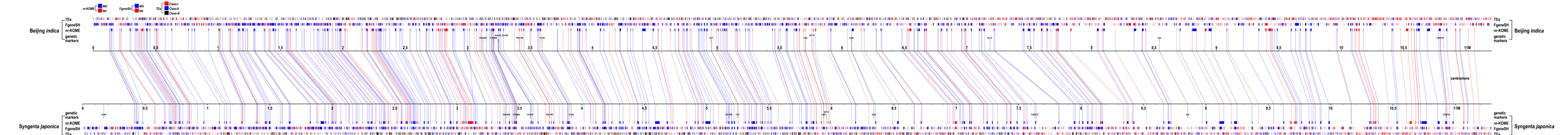

Supplement: Figure S6 — We depict all the genetic markers, nr-KOME cDNAs, FGENESH gene predictions, and transposable elements identified by RepeatMasker. Genes are depicted as WH (colored blue) or NH (colored red) based on their similarity to Arabidopsis. TEs are decomposed into classes I, II, and III. Correspondence between indica and japonica is indicated by drawing a connecting line between the 5′ ends of the nr-KOME cDNAs that clearly align to both assemblies. (9.6 MB ZIP). [file pbio.0030038.sg006.zip › two_piece/Chr11_L.pdf]

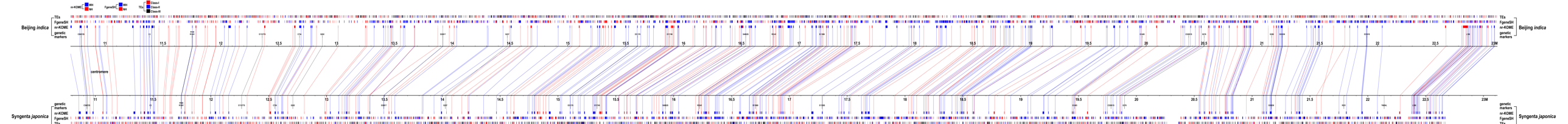

Supplement: Figure S6 — We depict all the genetic markers, nr-KOME cDNAs, FGENESH gene predictions, and transposable elements identified by RepeatMasker. Genes are depicted as WH (colored blue) or NH (colored red) based on their similarity to Arabidopsis. TEs are decomposed into classes I, II, and III. Correspondence between indica and japonica is indicated by drawing a connecting line between the 5′ ends of the nr-KOME cDNAs that clearly align to both assemblies. (9.6 MB ZIP). [file pbio.0030038.sg006.zip › two_piece/Chr11_R.pdf]

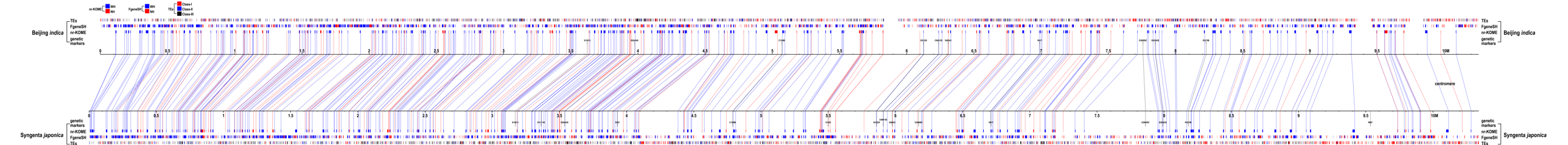

Supplement: Figure S6 — We depict all the genetic markers, nr-KOME cDNAs, FGENESH gene predictions, and transposable elements identified by RepeatMasker. Genes are depicted as WH (colored blue) or NH (colored red) based on their similarity to Arabidopsis. TEs are decomposed into classes I, II, and III. Correspondence between indica and japonica is indicated by drawing a connecting line between the 5′ ends of the nr-KOME cDNAs that clearly align to both assemblies. (9.6 MB ZIP). [file pbio.0030038.sg006.zip › two_piece/Chr12_L.pdf]

# Rice-Rice Comparison

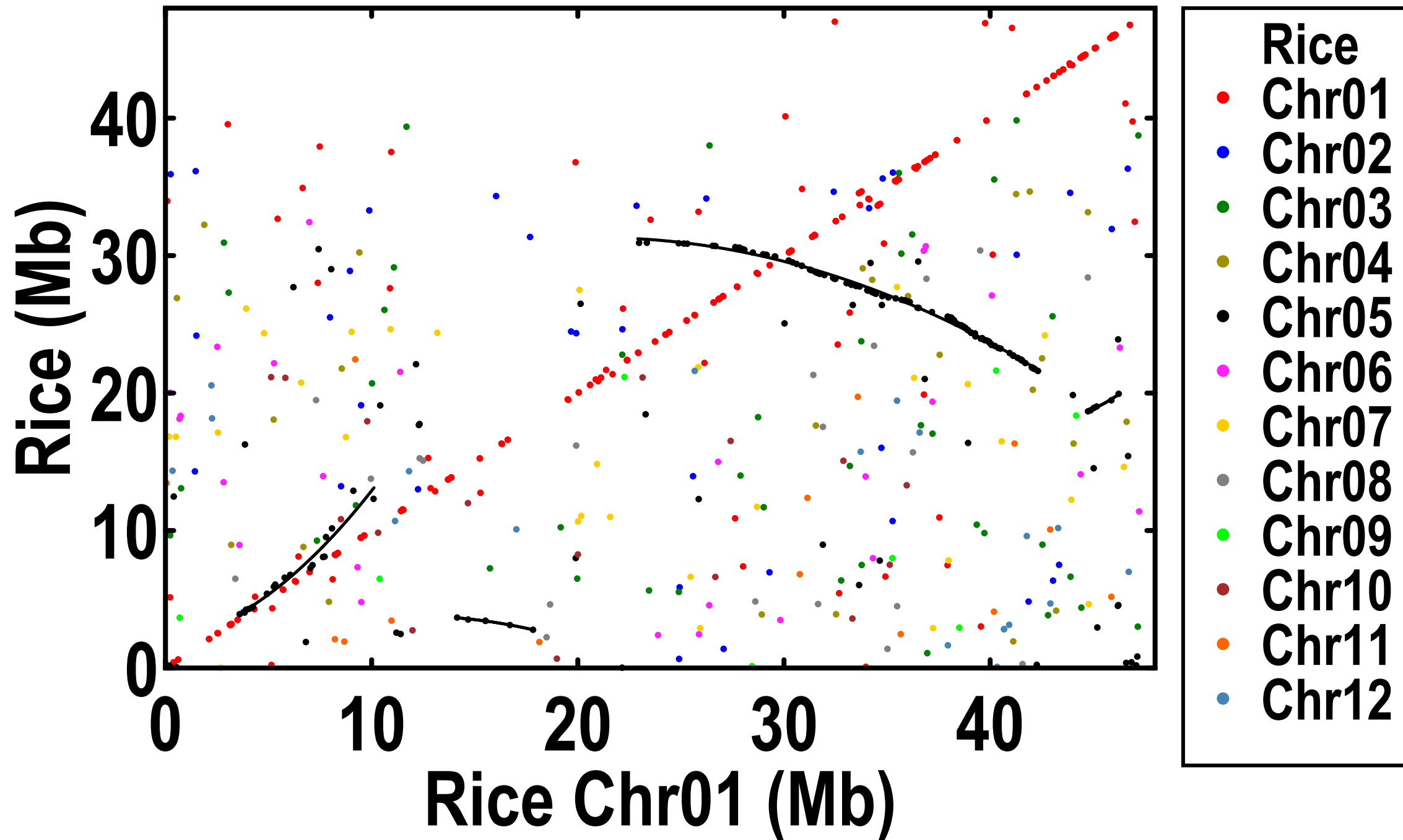

Supplement: Figure S7 — (507 KB ZIP). [file pbio.0030038.sg007.zip › webfig7_duplicated-chrs/Rice_Rice.Chr01.pdf]

# Rice-Rice Comparison

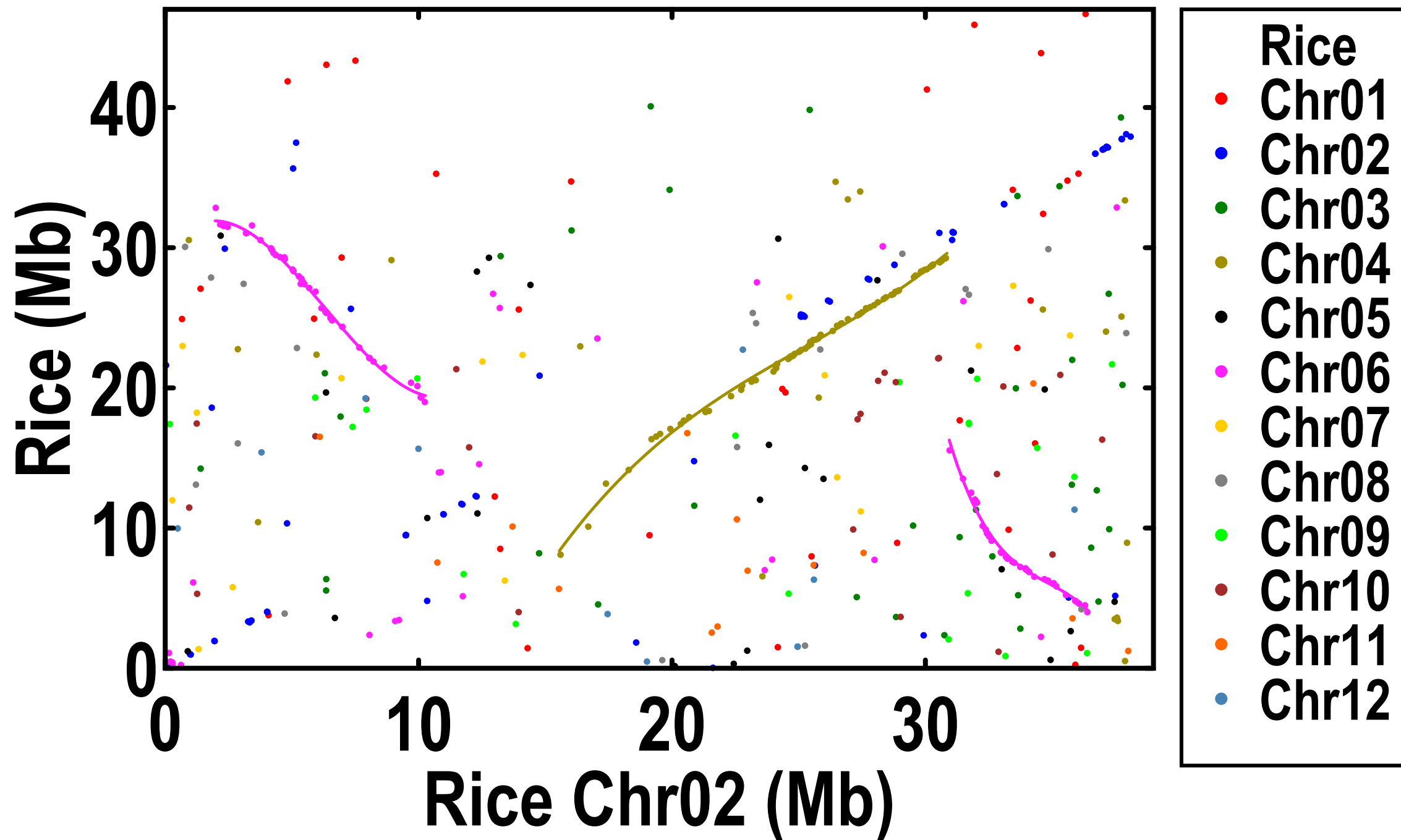

Supplement: Figure S7 — (507 KB ZIP). [file pbio.0030038.sg007.zip › webfig7_duplicated-chrs/Rice_Rice.Chr02.pdf]

# Rice-Rice Comparison

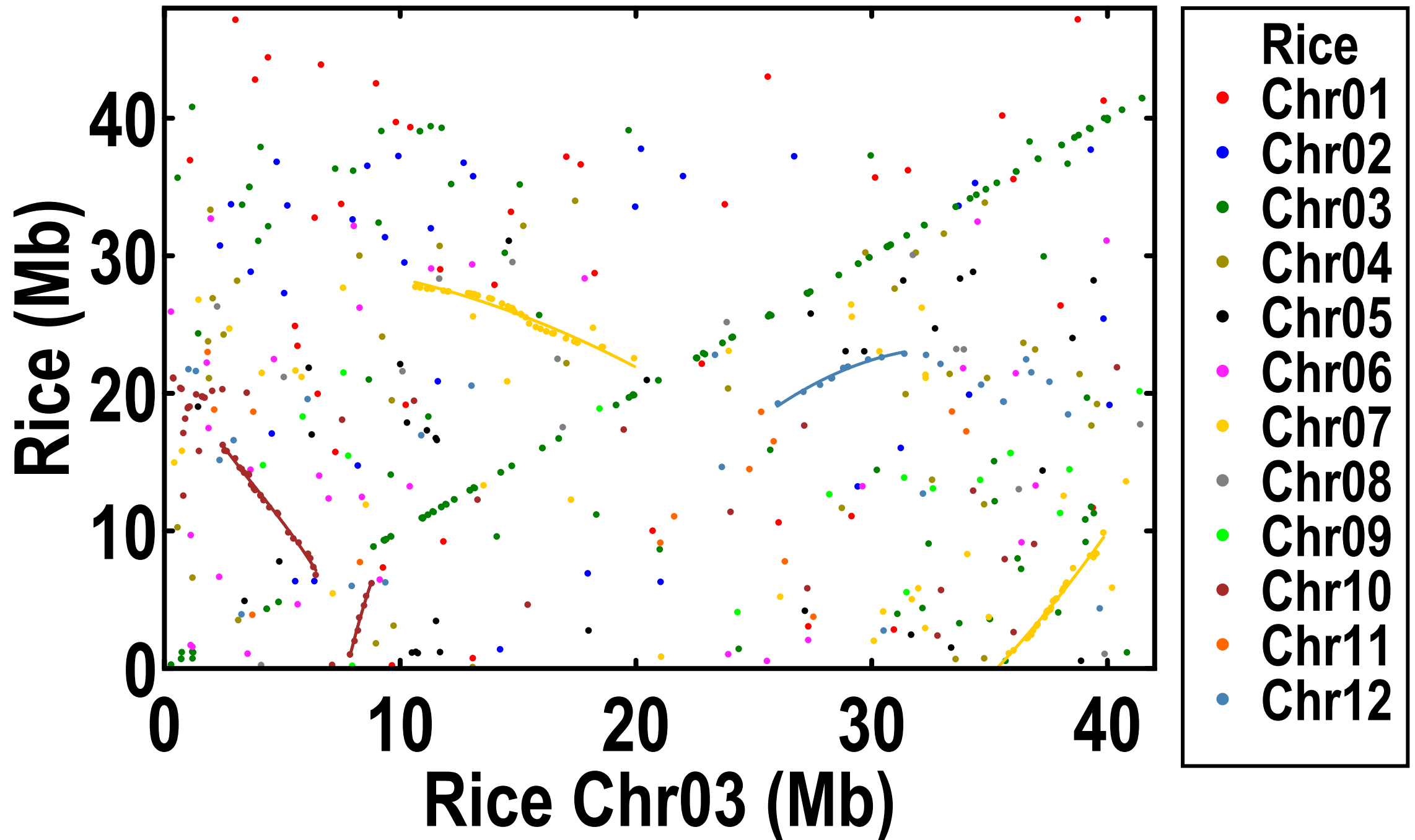

Supplement: Figure S7 — (507 KB ZIP). [file pbio.0030038.sg007.zip › webfig7_duplicated-chrs/Rice_Rice.Chr03.pdf]

# Rice-Rice Comparison

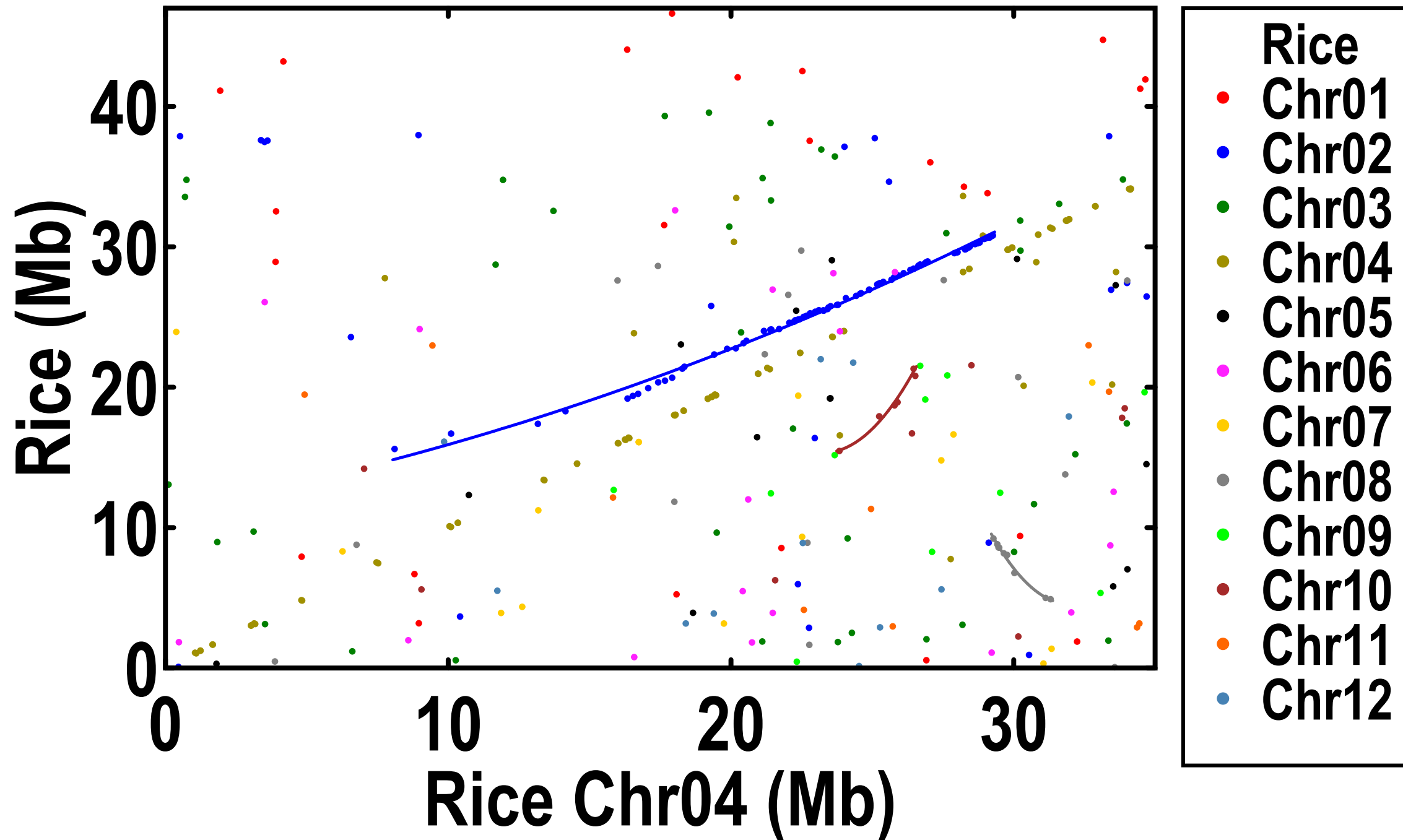

Supplement: Figure S7 — (507 KB ZIP). [file pbio.0030038.sg007.zip › webfig7_duplicated-chrs/Rice_Rice.Chr04.pdf]

# Rice-Rice Comparison

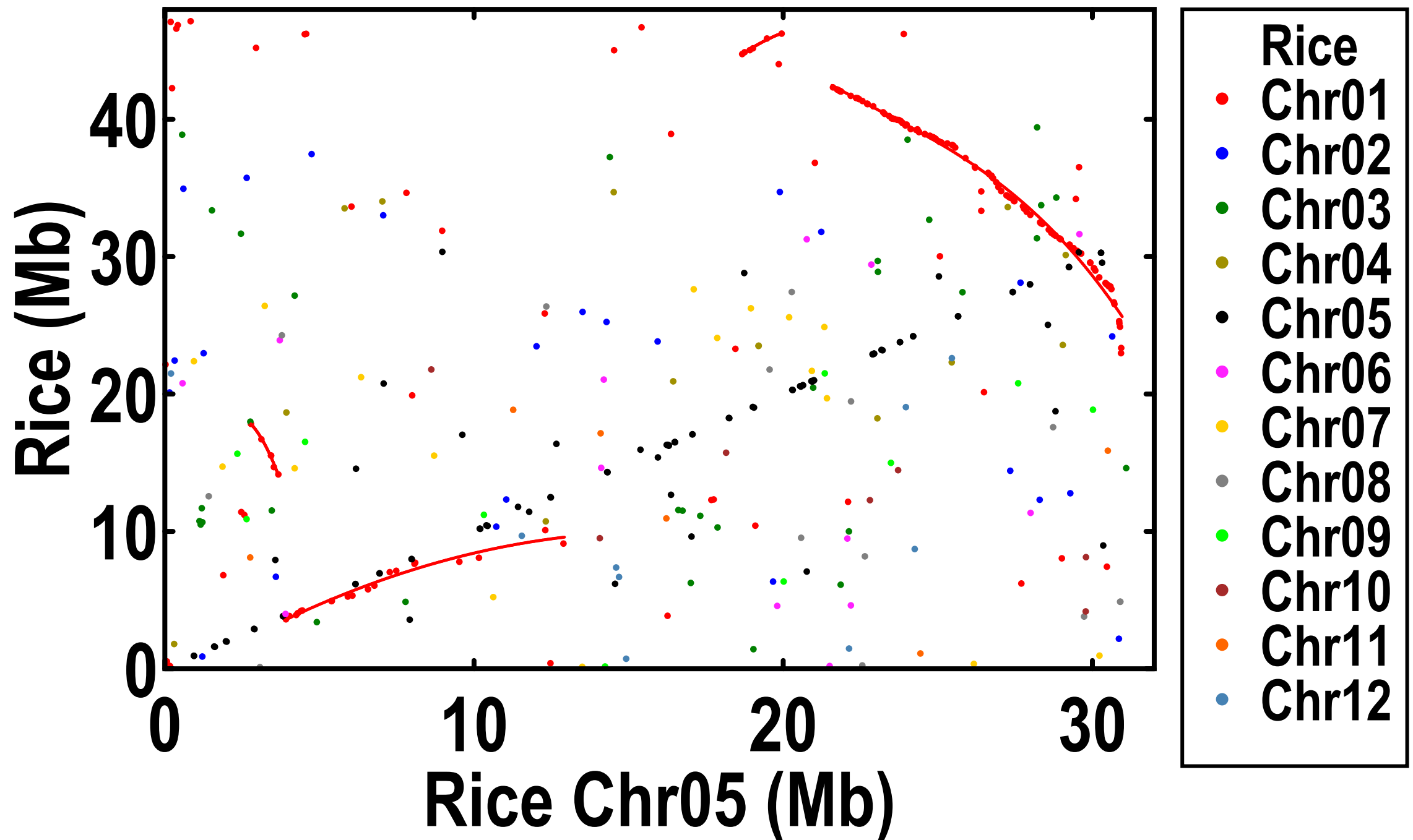

Supplement: Figure S7 — (507 KB ZIP). [file pbio.0030038.sg007.zip › webfig7_duplicated-chrs/Rice_Rice.Chr05.pdf]

# Rice-Rice Comparison

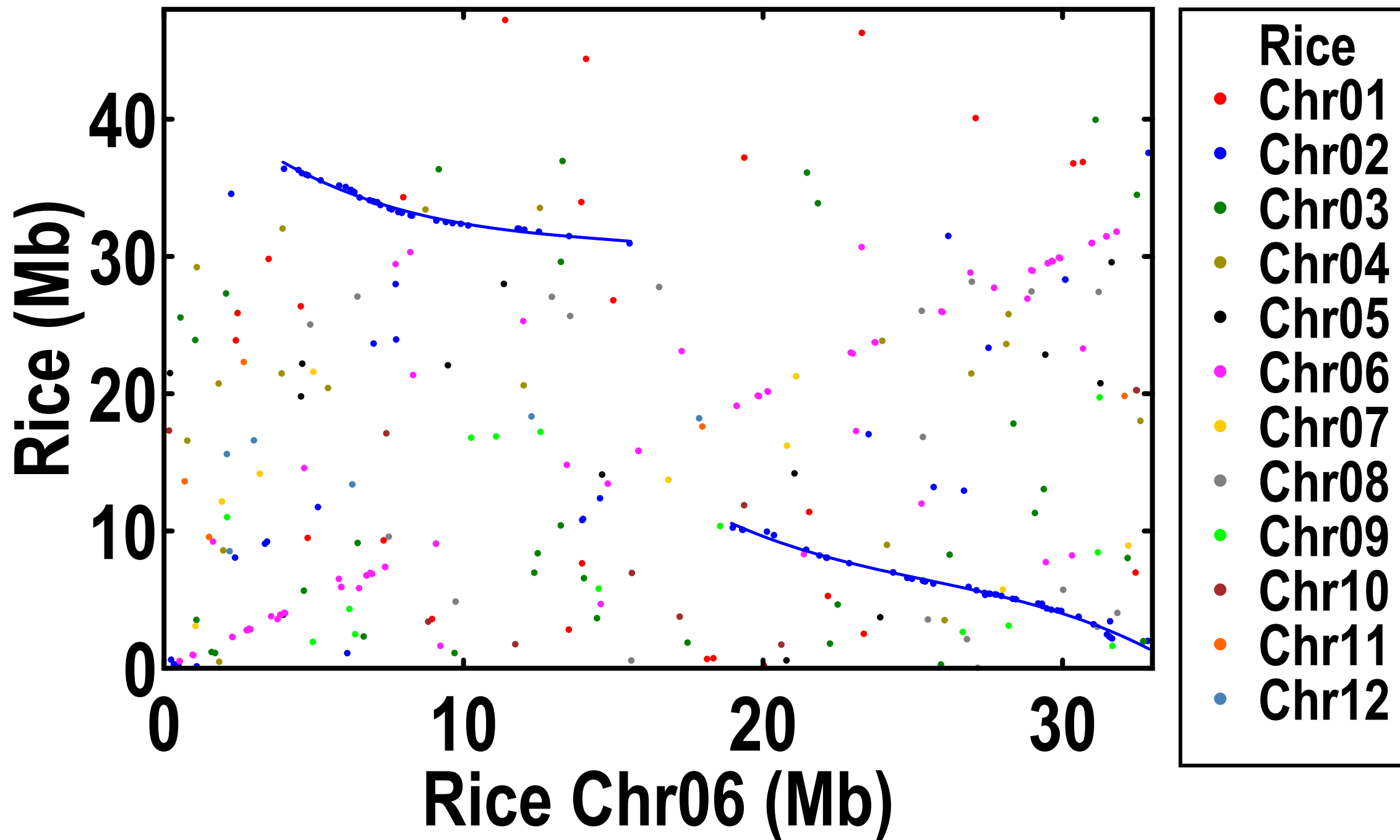

Supplement: Figure S7 — (507 KB ZIP). [file pbio.0030038.sg007.zip › webfig7_duplicated-chrs/Rice_Rice.Chr06.pdf]

# Rice-Rice Comparison

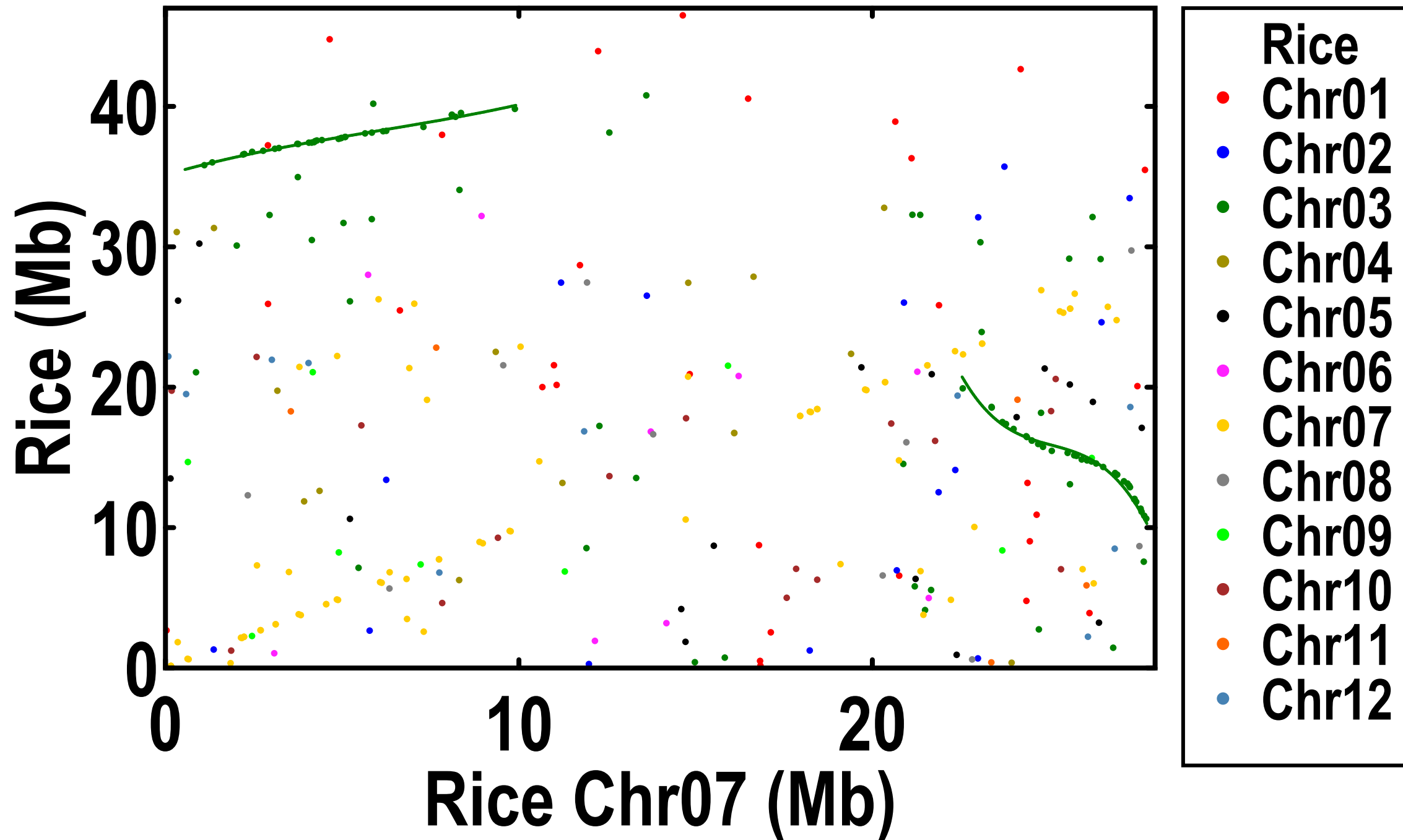

Supplement: Figure S7 — (507 KB ZIP). [file pbio.0030038.sg007.zip › webfig7_duplicated-chrs/Rice_Rice.Chr07.pdf]

# Rice-Rice Comparison

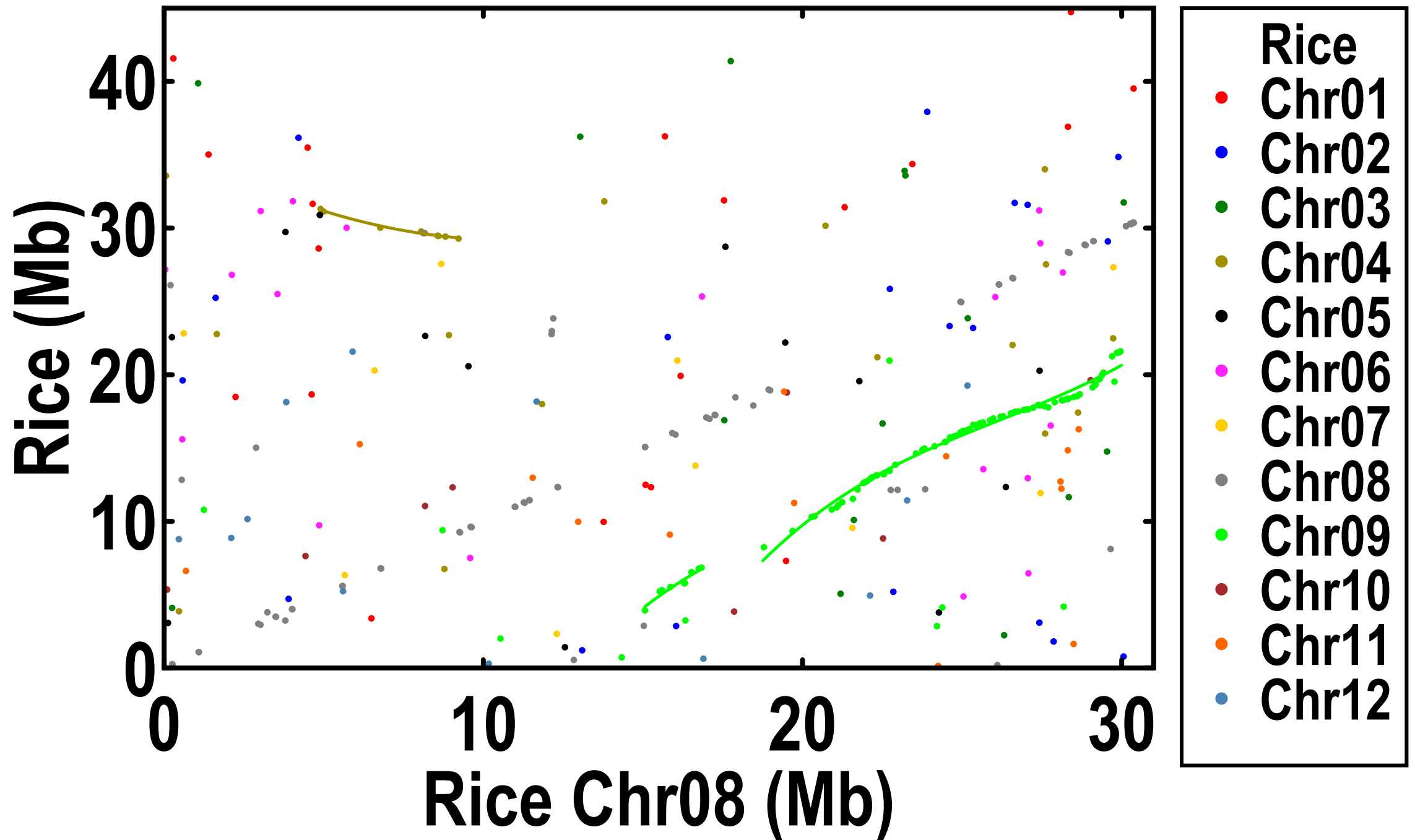

Supplement: Figure S7 — (507 KB ZIP). [file pbio.0030038.sg007.zip › webfig7_duplicated-chrs/Rice_Rice.Chr08.pdf]

# Rice-Rice Comparison

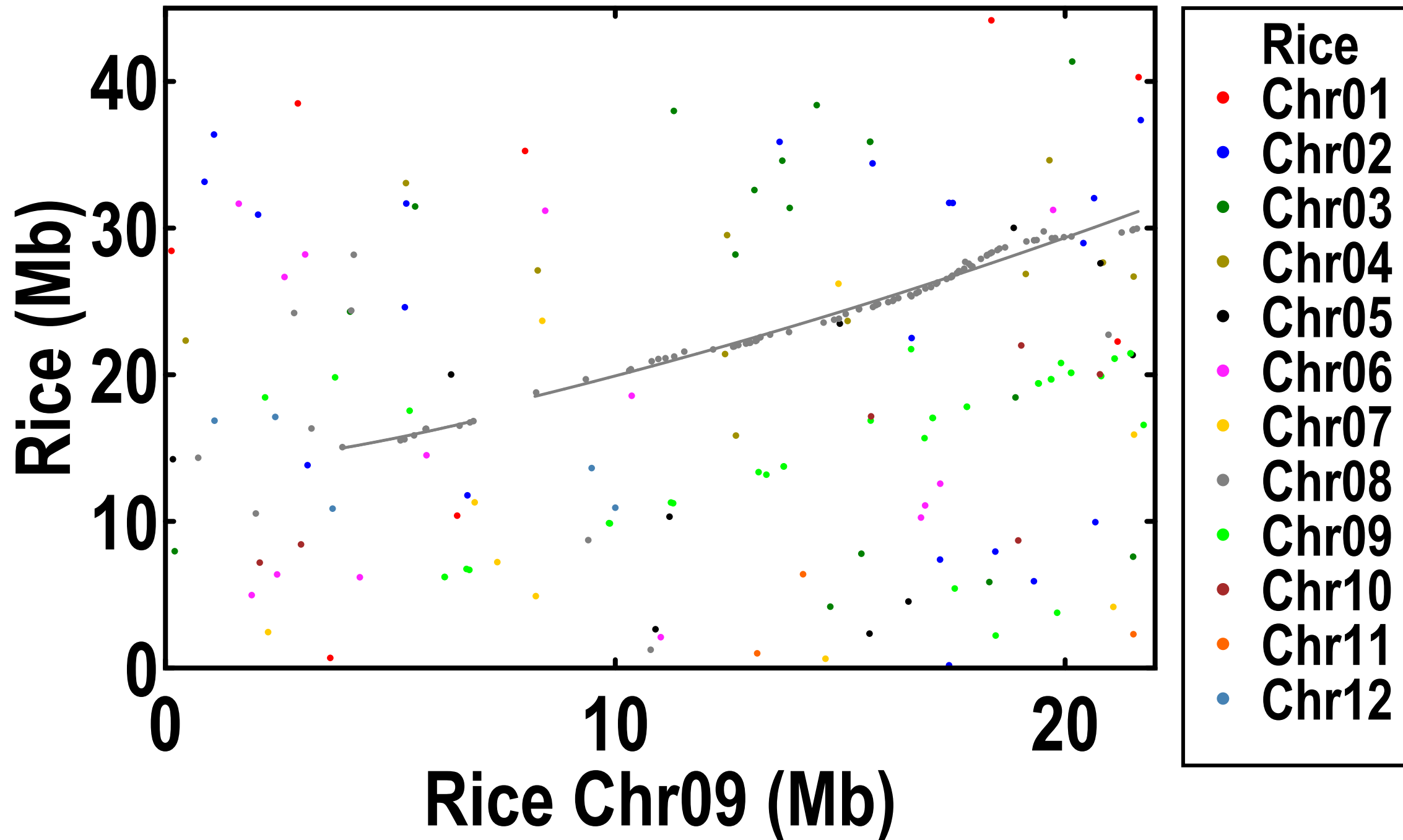

Supplement: Figure S7 — (507 KB ZIP). [file pbio.0030038.sg007.zip › webfig7_duplicated-chrs/Rice_Rice.Chr09.pdf]

# Rice-Rice Comparison

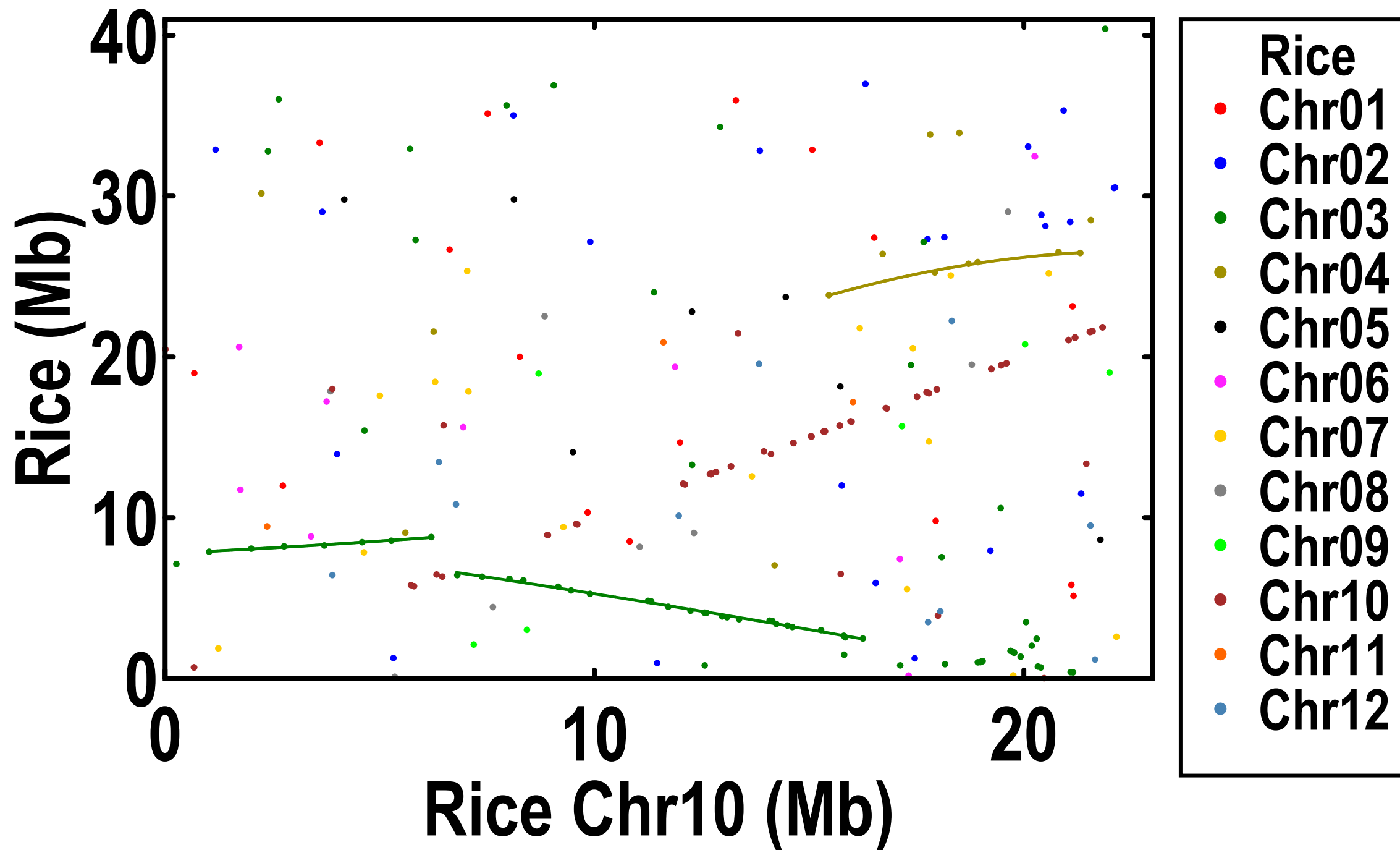

Supplement: Figure S7 — (507 KB ZIP). [file pbio.0030038.sg007.zip › webfig7_duplicated-chrs/Rice_Rice.Chr10.pdf]

# Rice-Rice Comparison

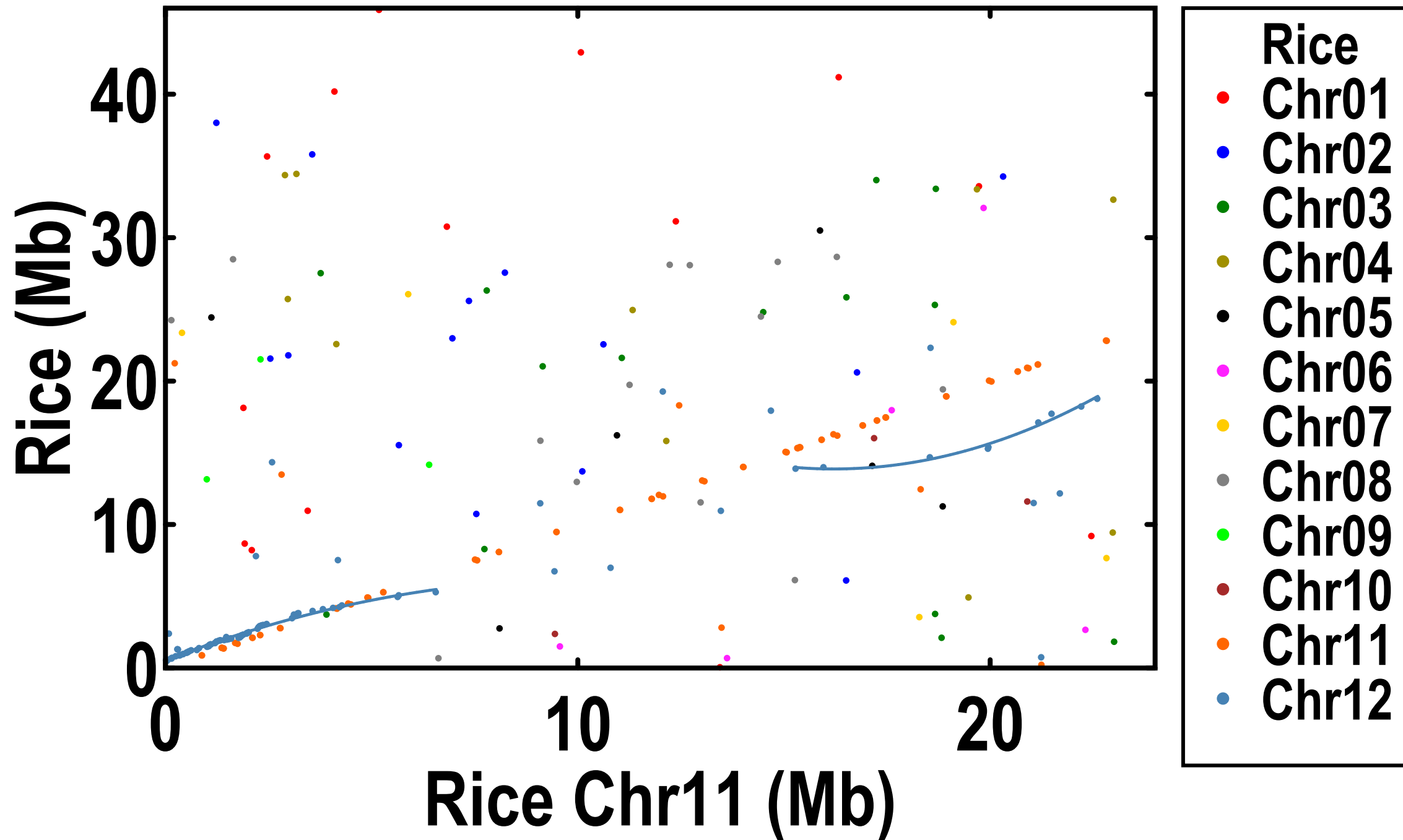

Supplement: Figure S7 — (507 KB ZIP). [file pbio.0030038.sg007.zip › webfig7_duplicated-chrs/Rice_Rice.Chr11.pdf]

# Rice-Rice Comparison

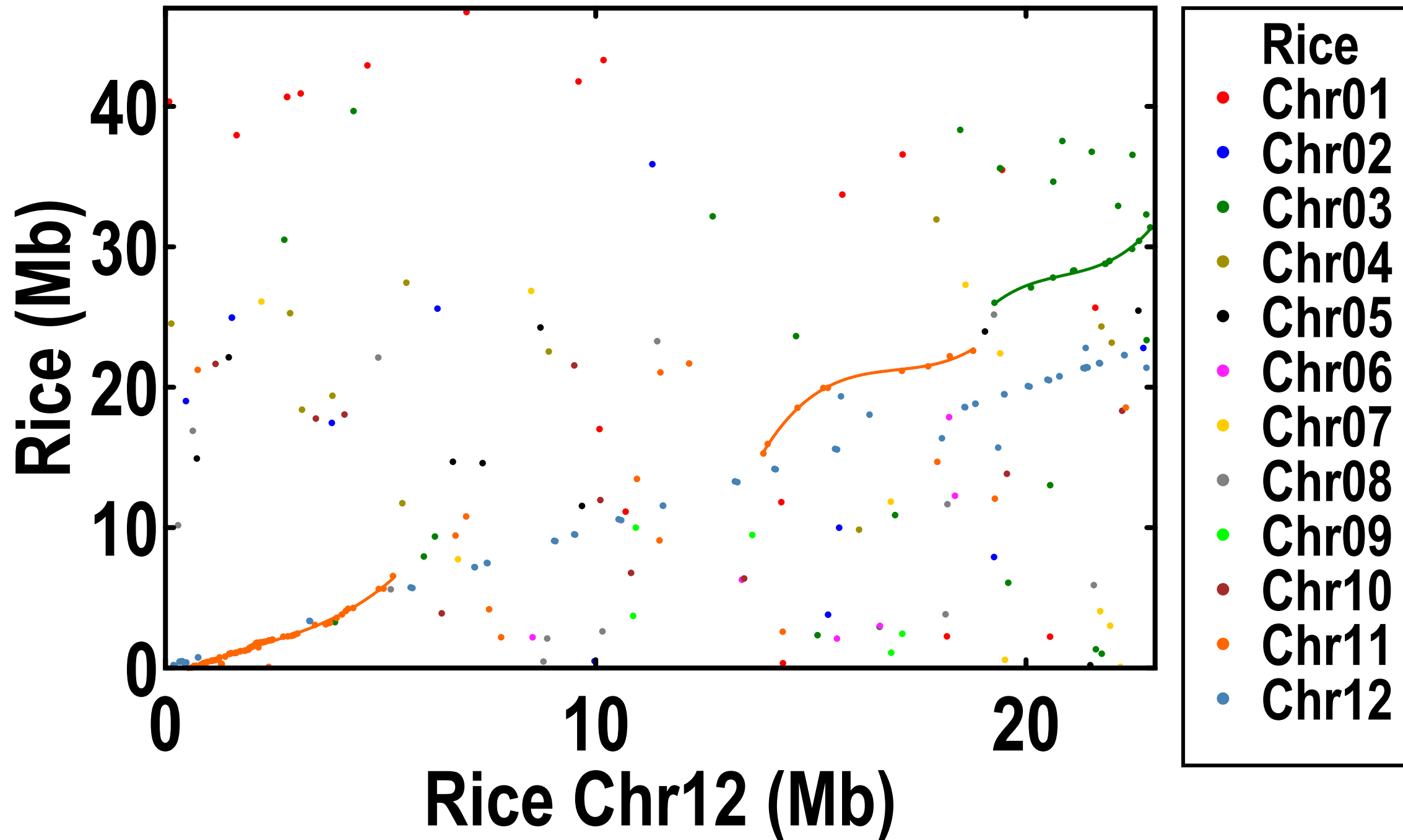

Supplement: Figure S7 — (507 KB ZIP). [file pbio.0030038.sg007.zip › webfig7_duplicated-chrs/Rice_Rice.Chr12.pdf]

# Rice-Maize Comparison

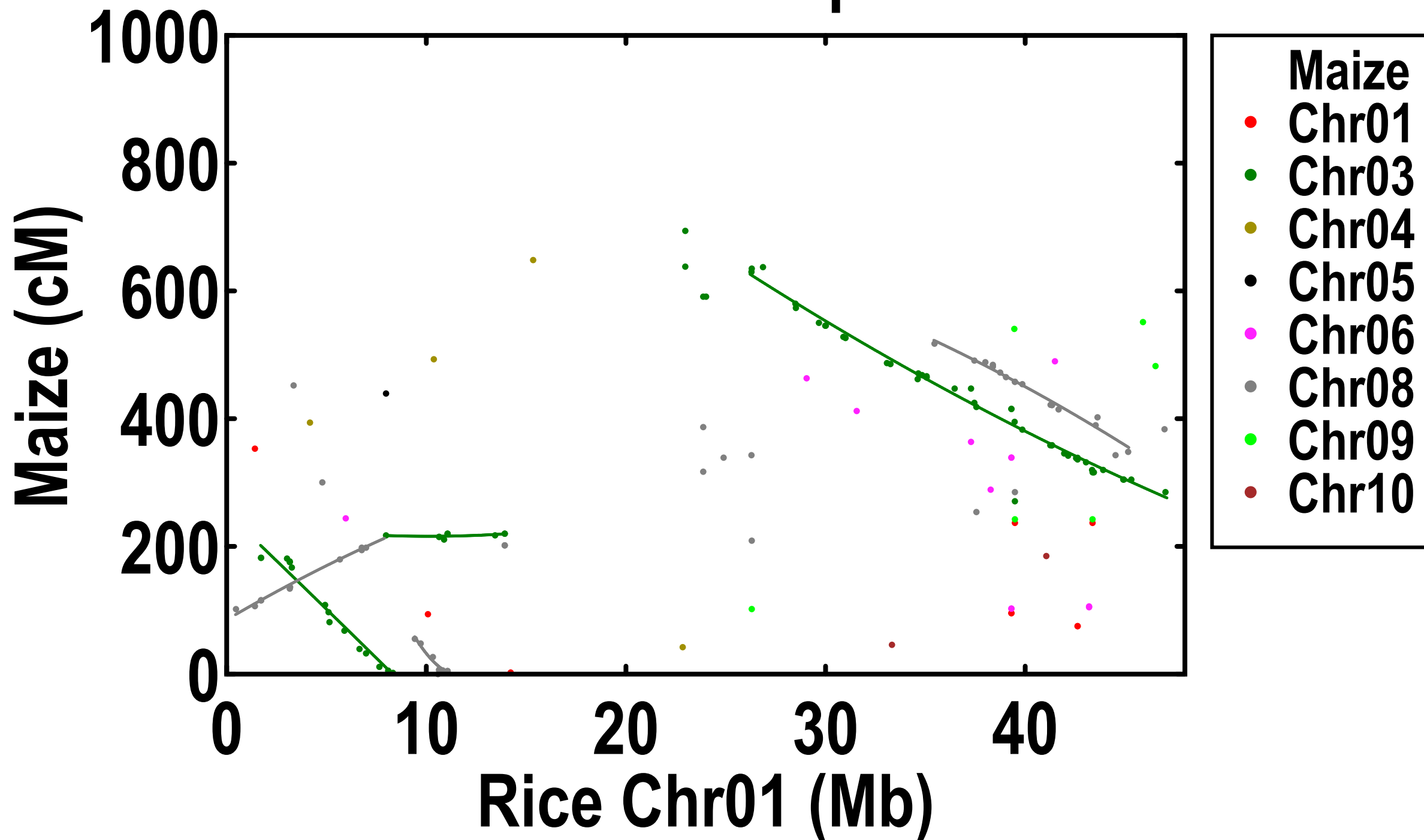

Supplement: Figure S8 — Each point indicates the genomic positions for a maize genetic marker and its highest confidence match in rice. The x-axis shows a specific chromosome for one genome, and the y-axis shows all chromosomes for a second genome, with the chromosome numbers color-coded as per the legend. We show here 12 panels for rice. (311 KB ZIP). [file pbio.0030038.sg008.zip › Rice/Maize_Rice.Chr01.pdf]

# Rice-Maize Comparison

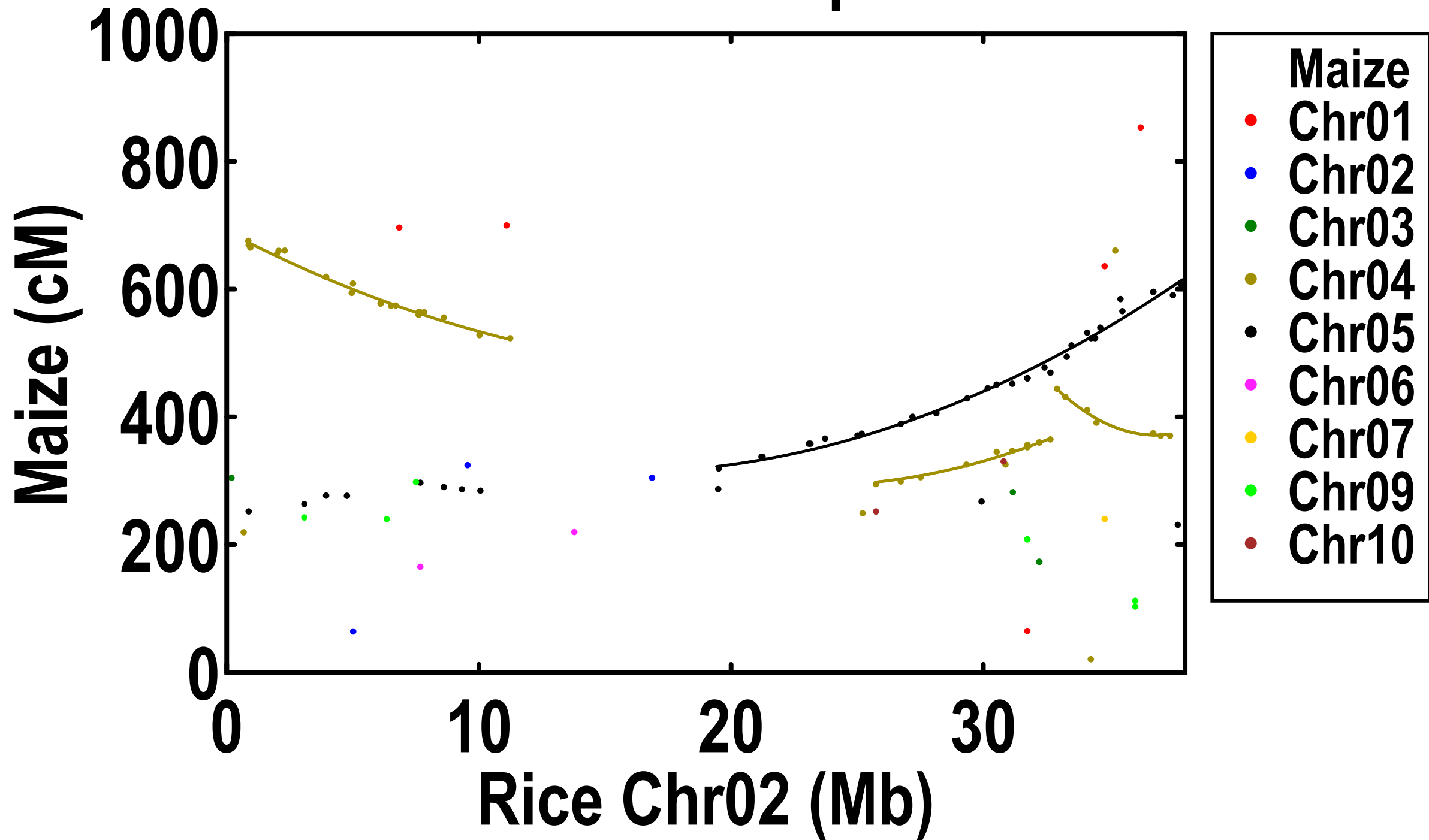

Supplement: Figure S8 — Each point indicates the genomic positions for a maize genetic marker and its highest confidence match in rice. The x-axis shows a specific chromosome for one genome, and the y-axis shows all chromosomes for a second genome, with the chromosome numbers color-coded as per the legend. We show here 12 panels for rice. (311 KB ZIP). [file pbio.0030038.sg008.zip › Rice/Maize_Rice.Chr02.pdf]

# Rice-Maize Comparison

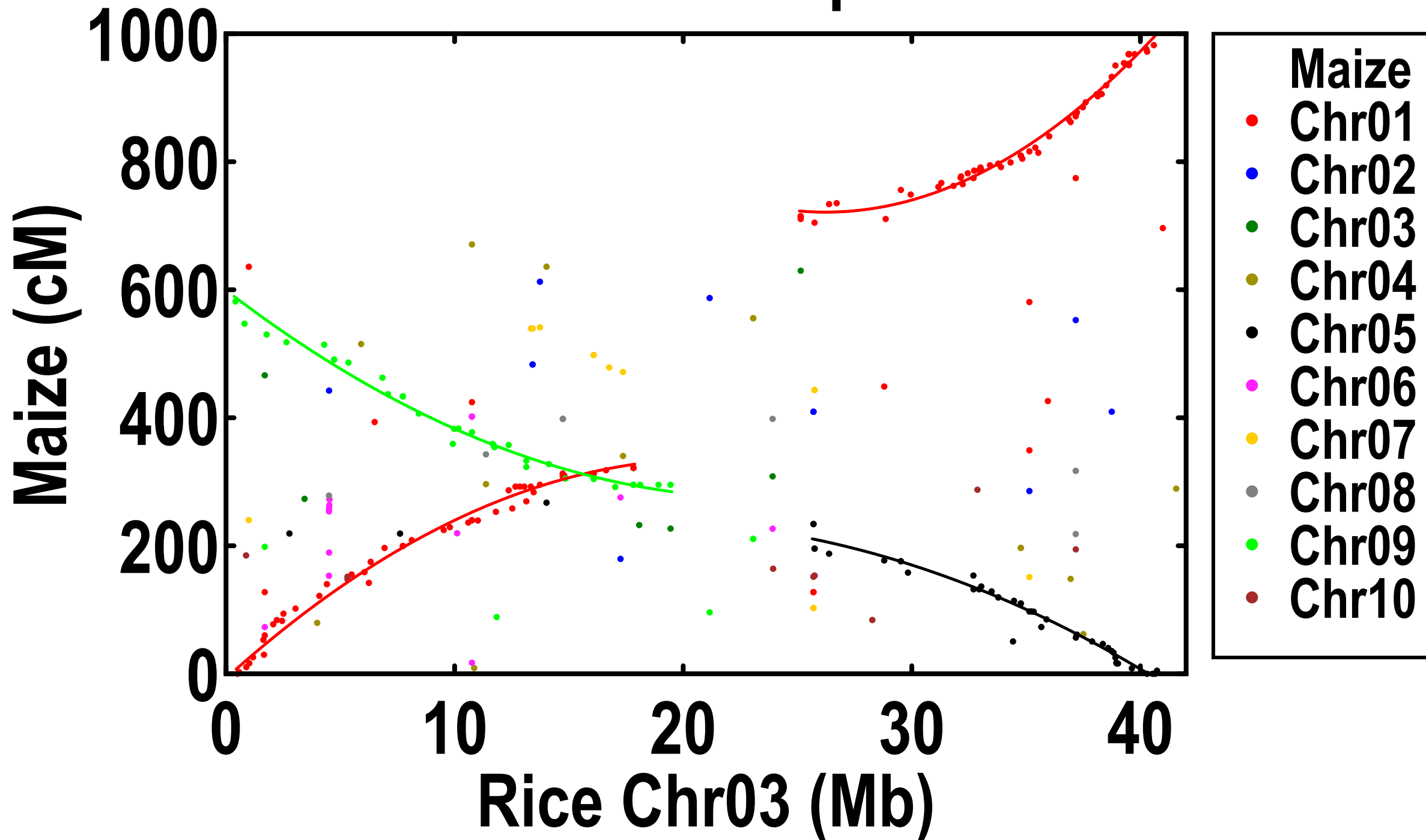

Supplement: Figure S8 — Each point indicates the genomic positions for a maize genetic marker and its highest confidence match in rice. The x-axis shows a specific chromosome for one genome, and the y-axis shows all chromosomes for a second genome, with the chromosome numbers color-coded as per the legend. We show here 12 panels for rice. (311 KB ZIP). [file pbio.0030038.sg008.zip › Rice/Maize_Rice.Chr03.pdf]

# Rice-Maize Comparison

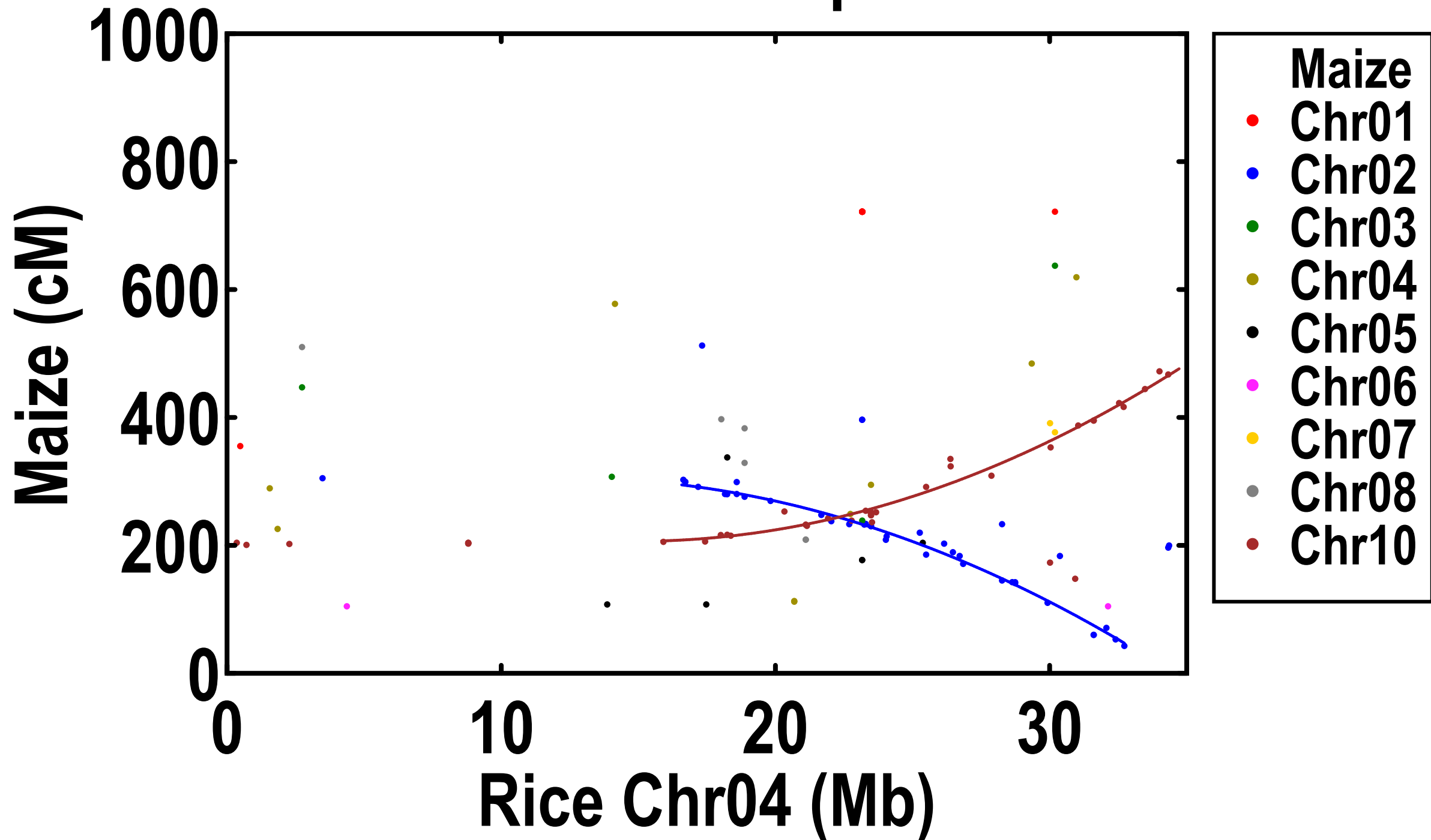

Supplement: Figure S8 — Each point indicates the genomic positions for a maize genetic marker and its highest confidence match in rice. The x-axis shows a specific chromosome for one genome, and the y-axis shows all chromosomes for a second genome, with the chromosome numbers color-coded as per the legend. We show here 12 panels for rice. (311 KB ZIP). [file pbio.0030038.sg008.zip › Rice/Maize_Rice.Chr04.pdf]

# Rice-Maize Comparison

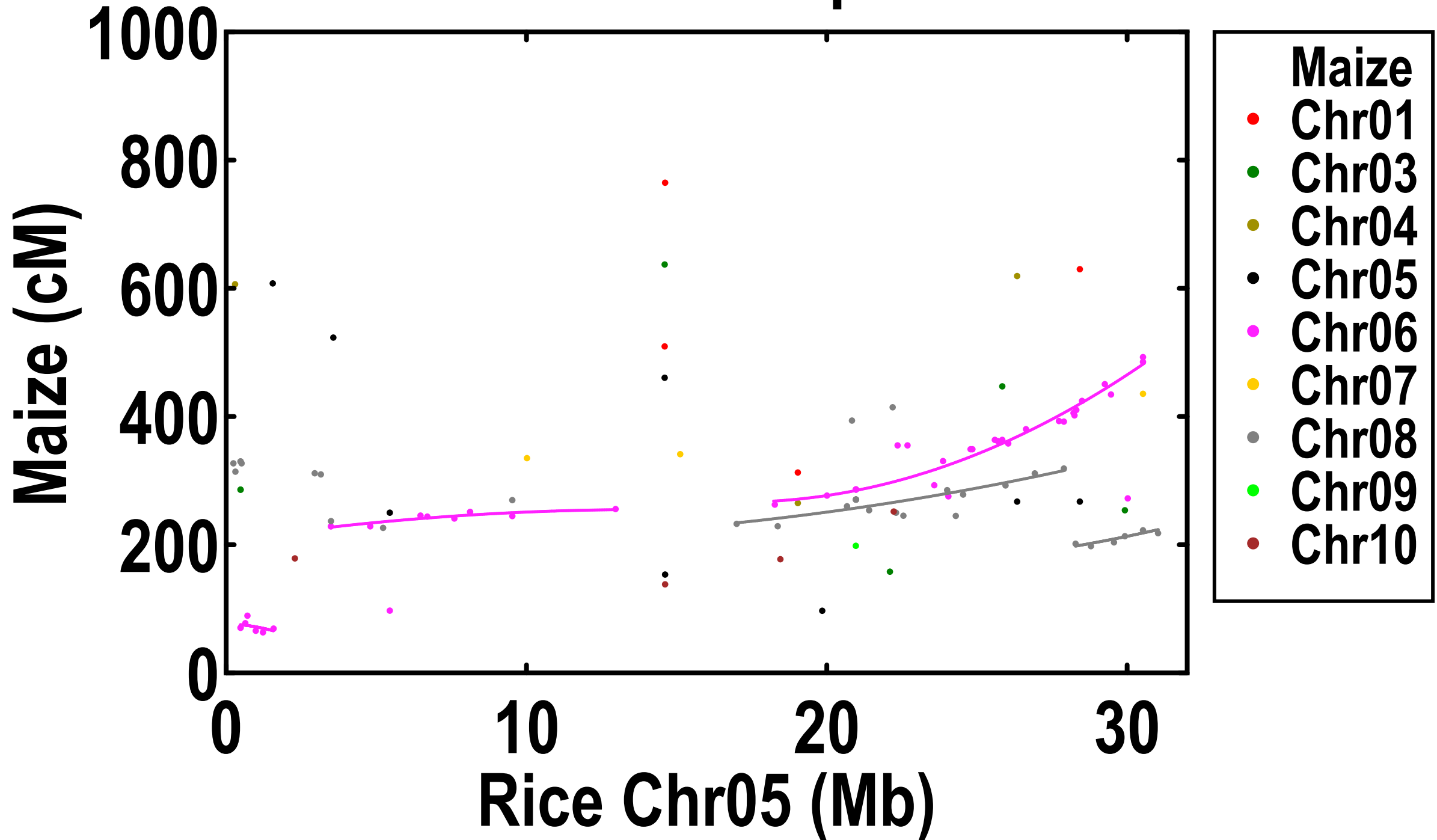

Supplement: Figure S8 — Each point indicates the genomic positions for a maize genetic marker and its highest confidence match in rice. The x-axis shows a specific chromosome for one genome, and the y-axis shows all chromosomes for a second genome, with the chromosome numbers color-coded as per the legend. We show here 12 panels for rice. (311 KB ZIP). [file pbio.0030038.sg008.zip › Rice/Maize_Rice.Chr05.pdf]

# Rice-Maize Comparison

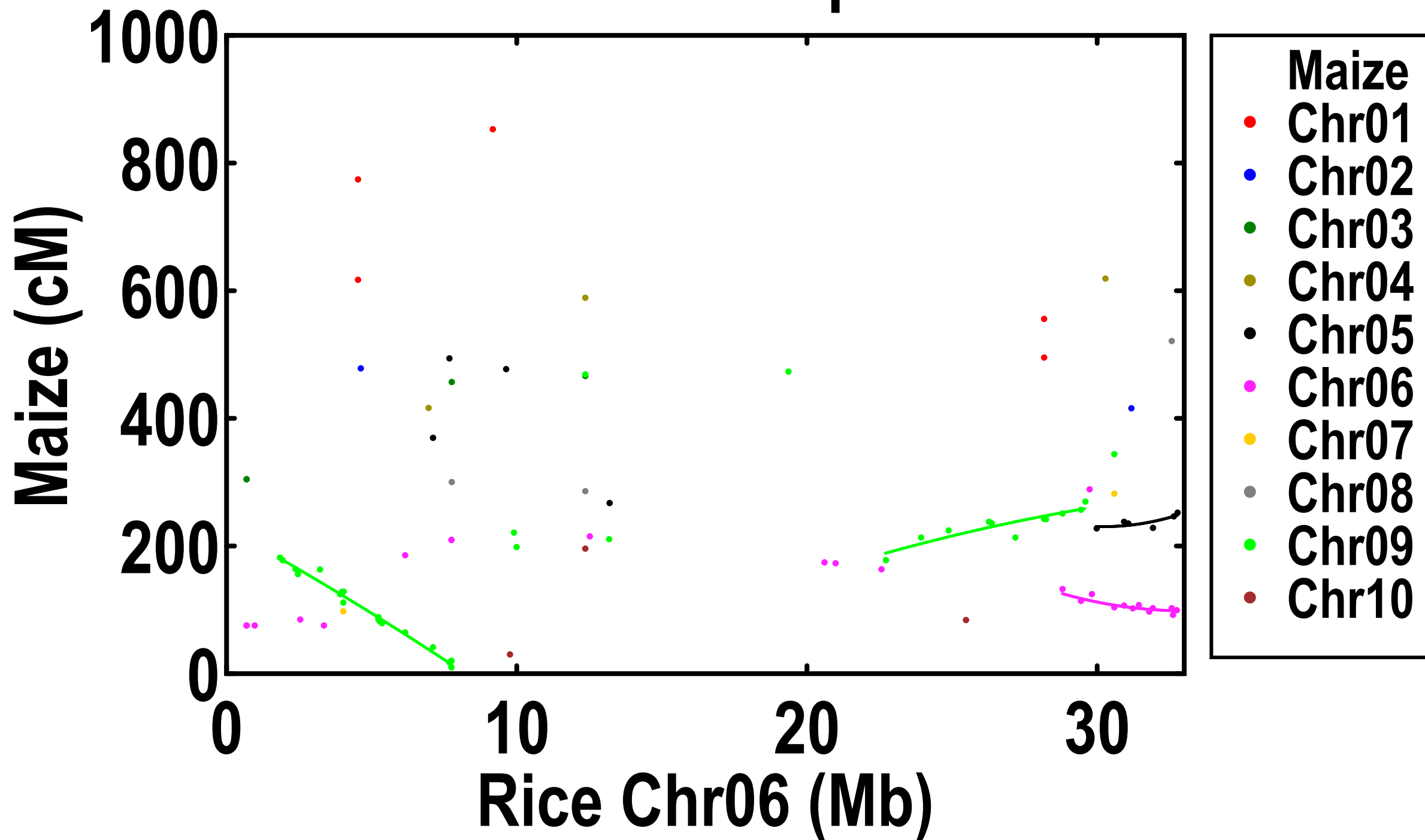

Supplement: Figure S8 — Each point indicates the genomic positions for a maize genetic marker and its highest confidence match in rice. The x-axis shows a specific chromosome for one genome, and the y-axis shows all chromosomes for a second genome, with the chromosome numbers color-coded as per the legend. We show here 12 panels for rice. (311 KB ZIP). [file pbio.0030038.sg008.zip › Rice/Maize_Rice.Chr06.pdf]

# Rice-Maize Comparison

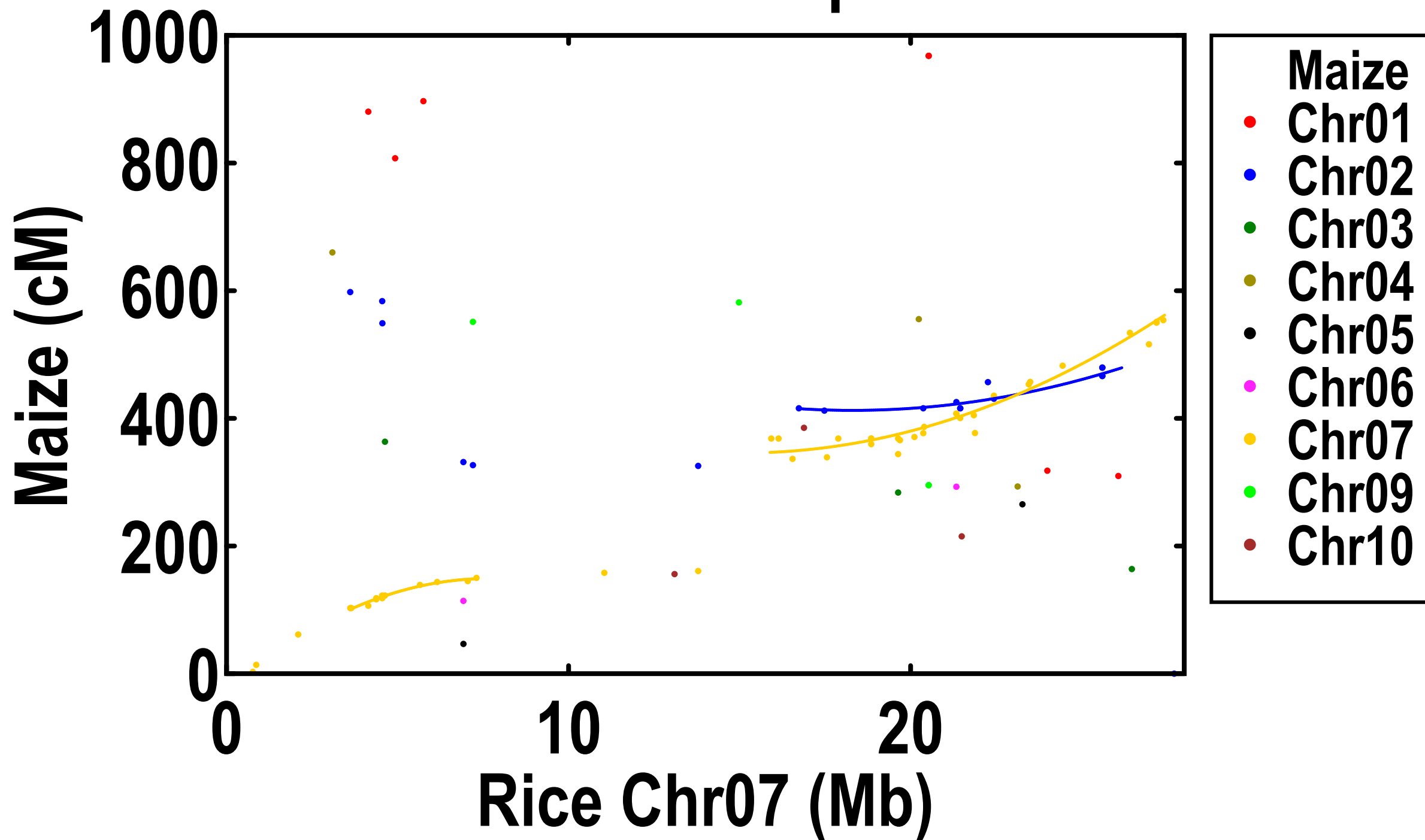

Supplement: Figure S8 — Each point indicates the genomic positions for a maize genetic marker and its highest confidence match in rice. The x-axis shows a specific chromosome for one genome, and the y-axis shows all chromosomes for a second genome, with the chromosome numbers color-coded as per the legend. We show here 12 panels for rice. (311 KB ZIP). [file pbio.0030038.sg008.zip › Rice/Maize_Rice.Chr07.pdf]

# Rice-Maize Comparison

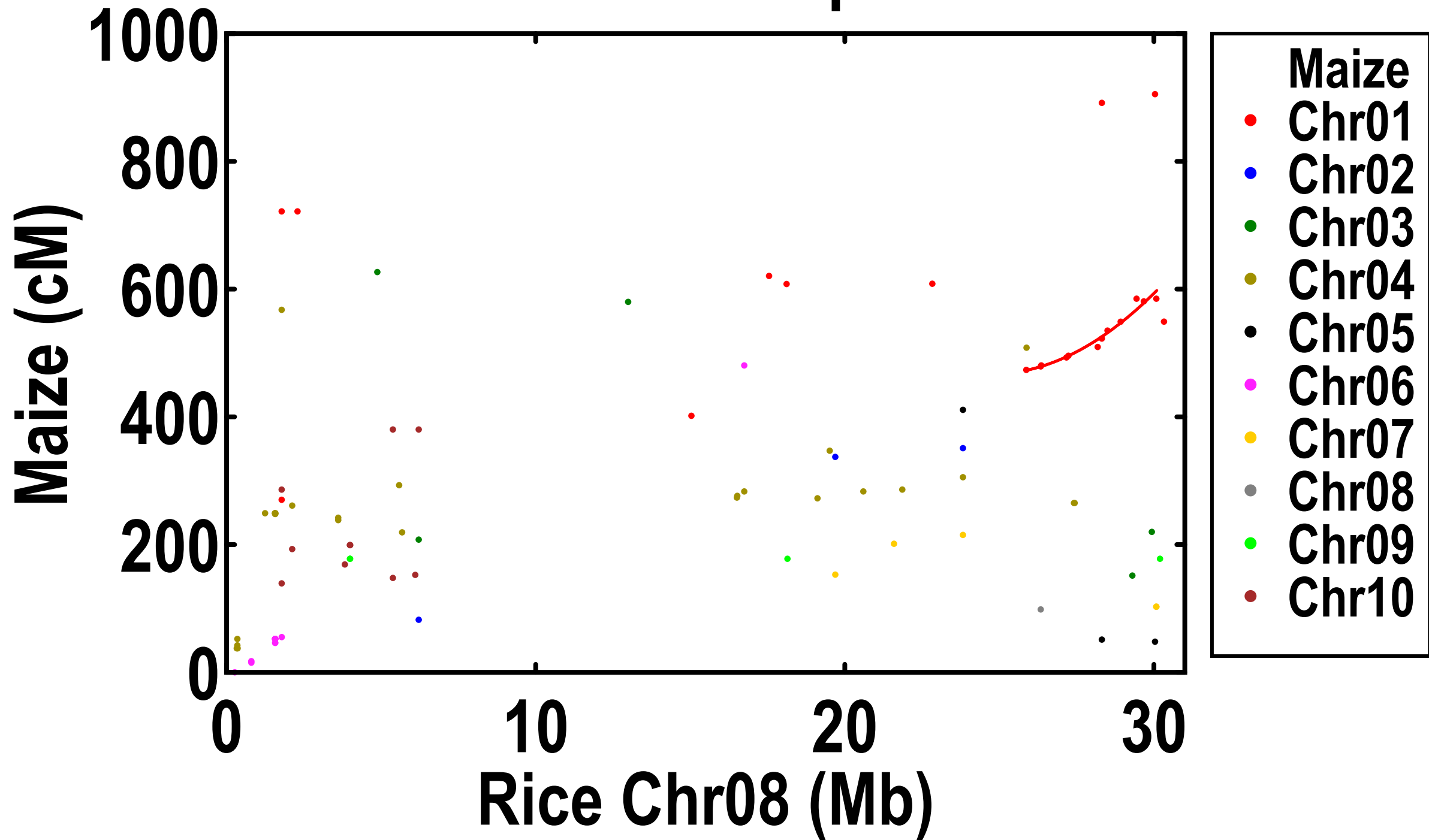

Supplement: Figure S8 — Each point indicates the genomic positions for a maize genetic marker and its highest confidence match in rice. The x-axis shows a specific chromosome for one genome, and the y-axis shows all chromosomes for a second genome, with the chromosome numbers color-coded as per the legend. We show here 12 panels for rice. (311 KB ZIP). [file pbio.0030038.sg008.zip › Rice/Maize_Rice.Chr08.pdf]

# Rice-Maize Comparison

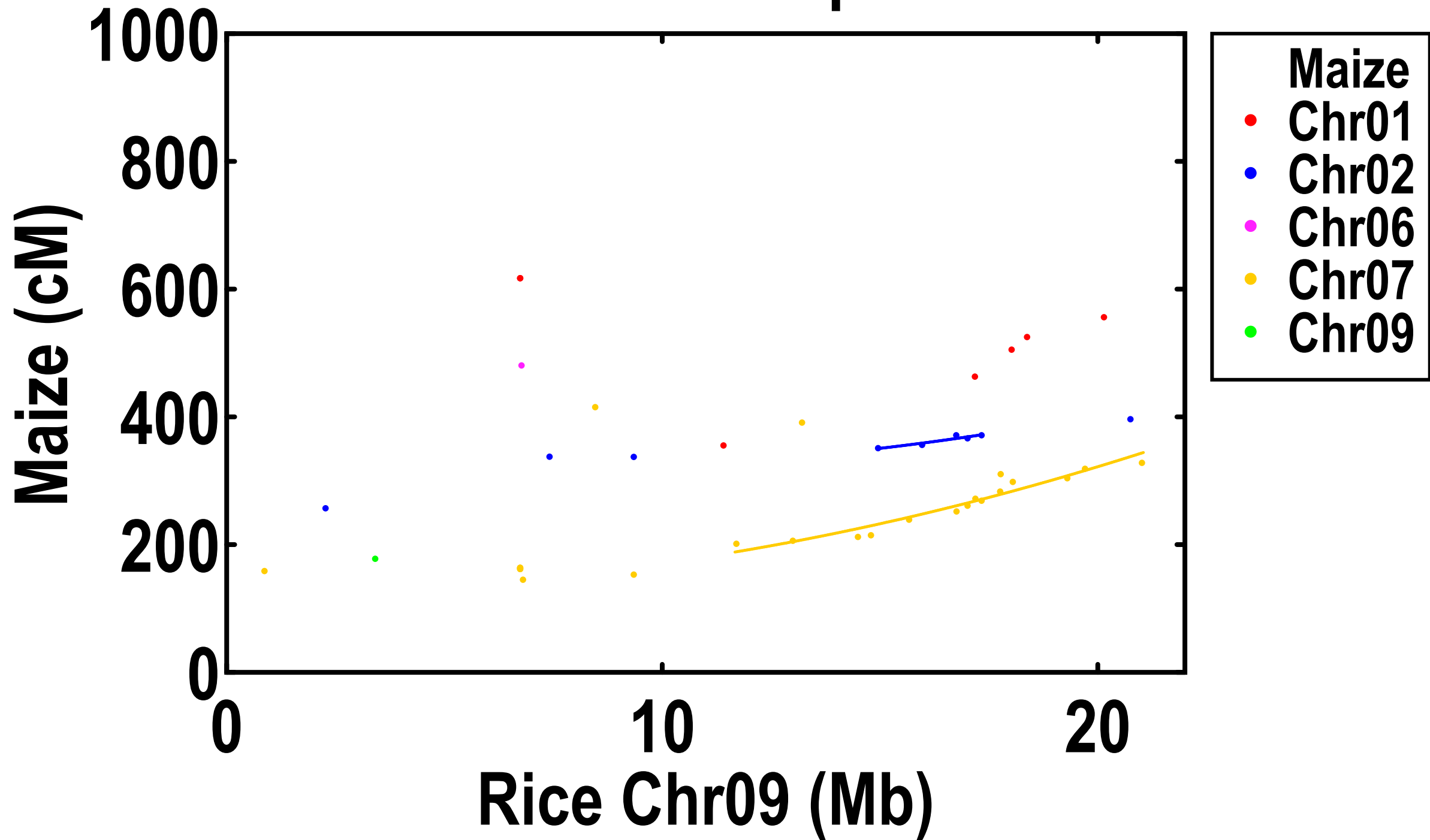

Supplement: Figure S8 — Each point indicates the genomic positions for a maize genetic marker and its highest confidence match in rice. The x-axis shows a specific chromosome for one genome, and the y-axis shows all chromosomes for a second genome, with the chromosome numbers color-coded as per the legend. We show here 12 panels for rice. (311 KB ZIP). [file pbio.0030038.sg008.zip › Rice/Maize_Rice.Chr09.pdf]

# Rice-Maize Comparison

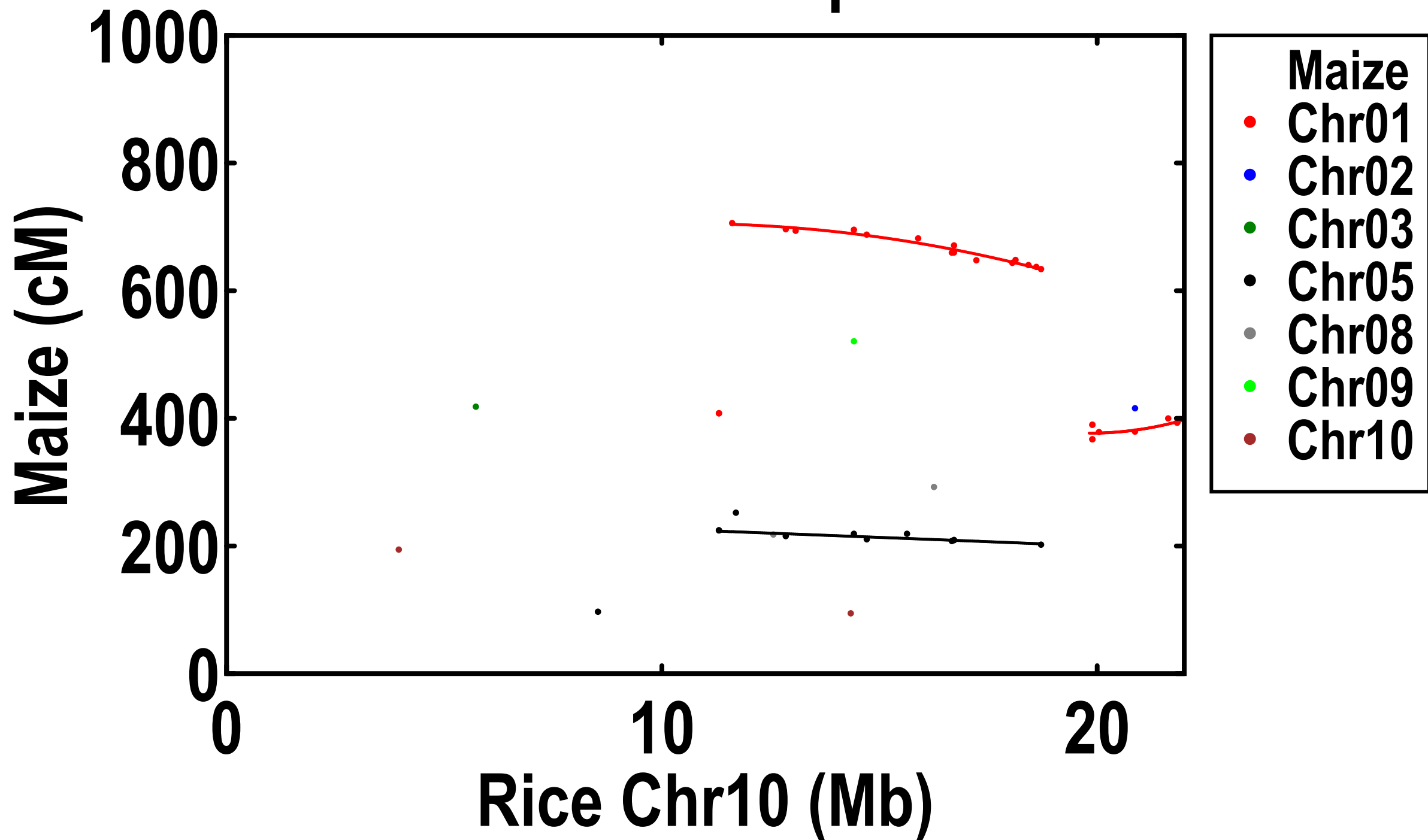

Supplement: Figure S8 — Each point indicates the genomic positions for a maize genetic marker and its highest confidence match in rice. The x-axis shows a specific chromosome for one genome, and the y-axis shows all chromosomes for a second genome, with the chromosome numbers color-coded as per the legend. We show here 12 panels for rice. (311 KB ZIP). [file pbio.0030038.sg008.zip › Rice/Maize_Rice.Chr10.pdf]

# Rice-Maize Comparison

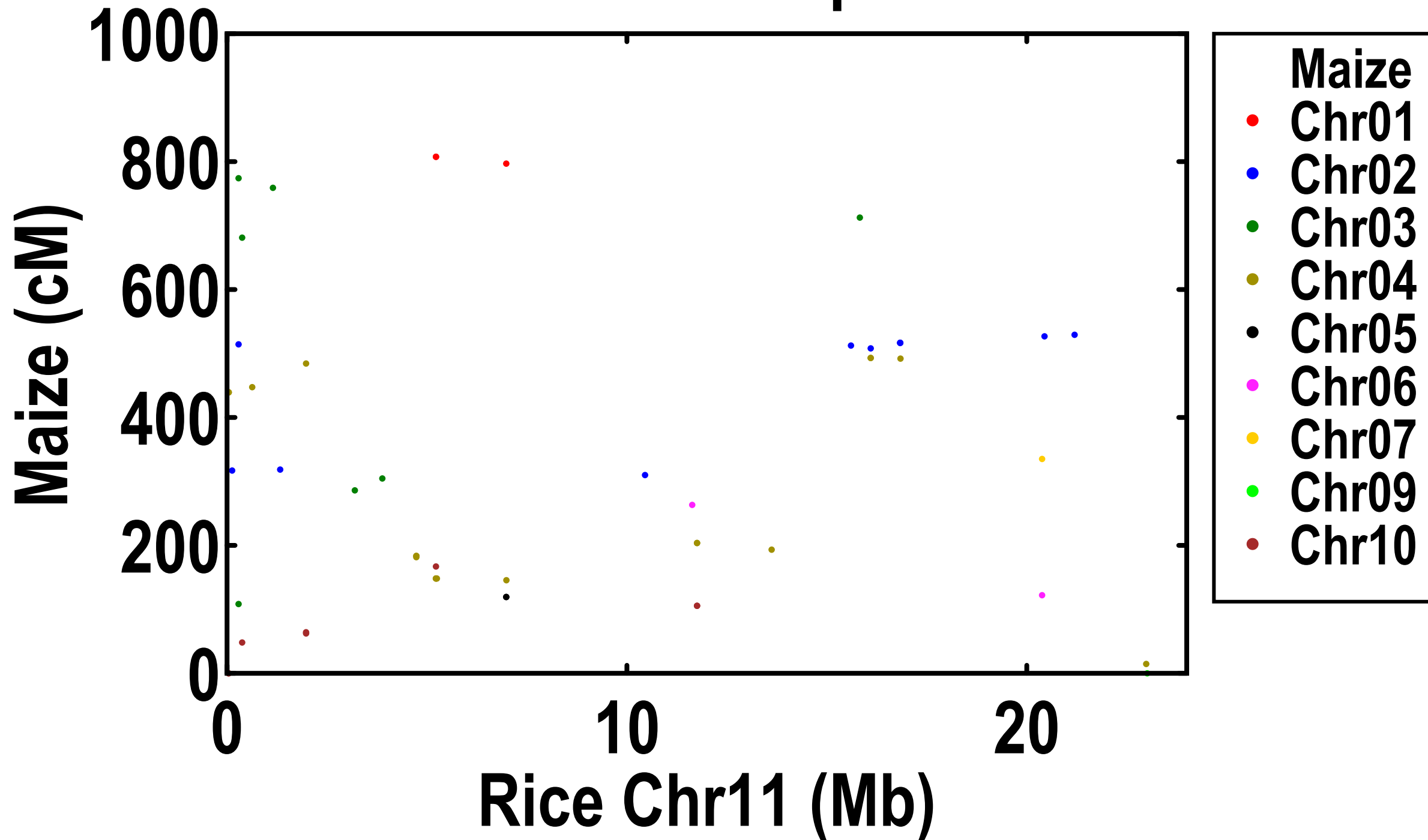

Supplement: Figure S8 — Each point indicates the genomic positions for a maize genetic marker and its highest confidence match in rice. The x-axis shows a specific chromosome for one genome, and the y-axis shows all chromosomes for a second genome, with the chromosome numbers color-coded as per the legend. We show here 12 panels for rice. (311 KB ZIP). [file pbio.0030038.sg008.zip › Rice/Maize_Rice.Chr11.pdf]

# Rice-Maize Comparison

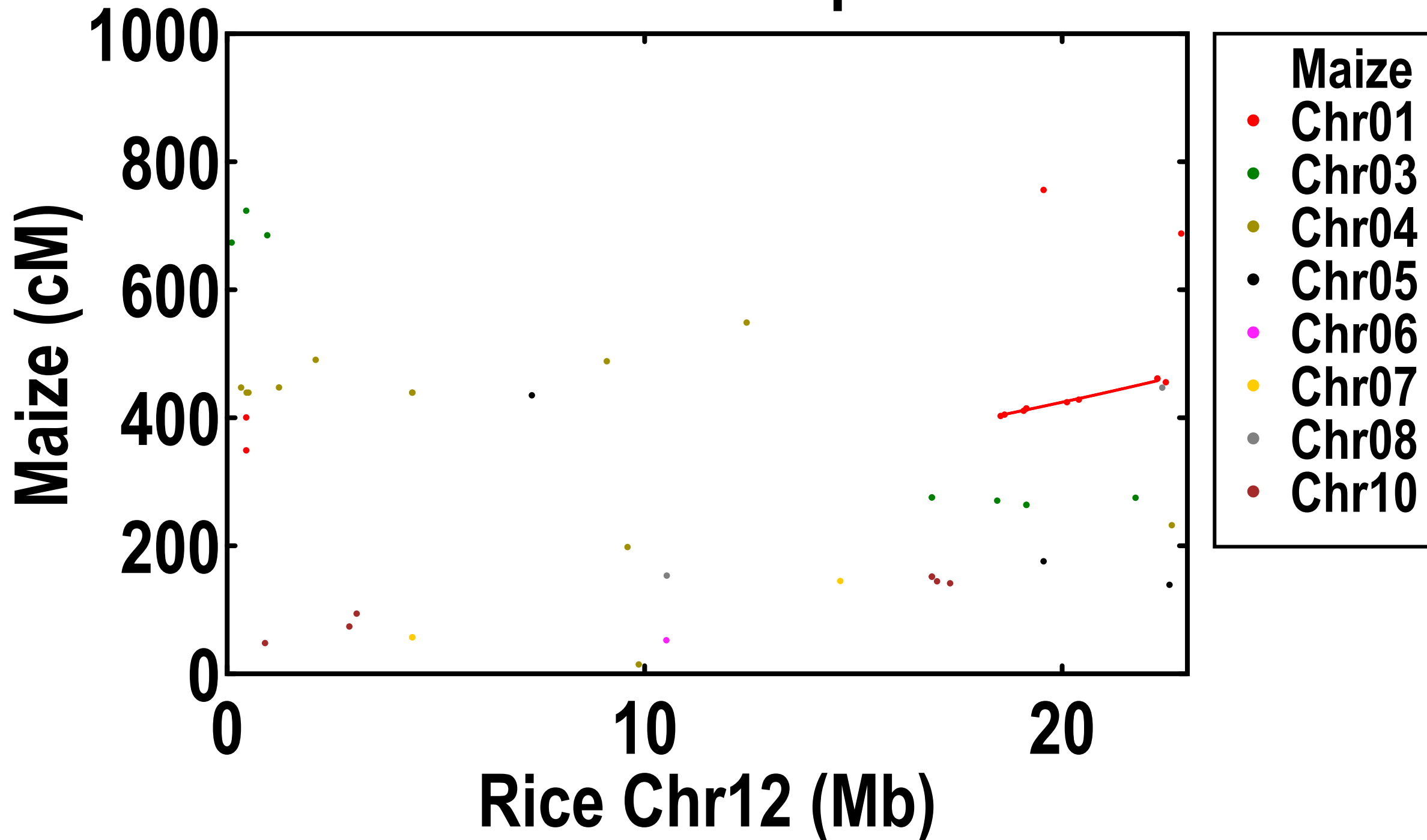

Supplement: Figure S8 — Each point indicates the genomic positions for a maize genetic marker and its highest confidence match in rice. The x-axis shows a specific chromosome for one genome, and the y-axis shows all chromosomes for a second genome, with the chromosome numbers color-coded as per the legend. We show here 12 panels for rice. (311 KB ZIP). [file pbio.0030038.sg008.zip › Rice/Maize_Rice.Chr12.pdf]

# Maize-Rice Comparison

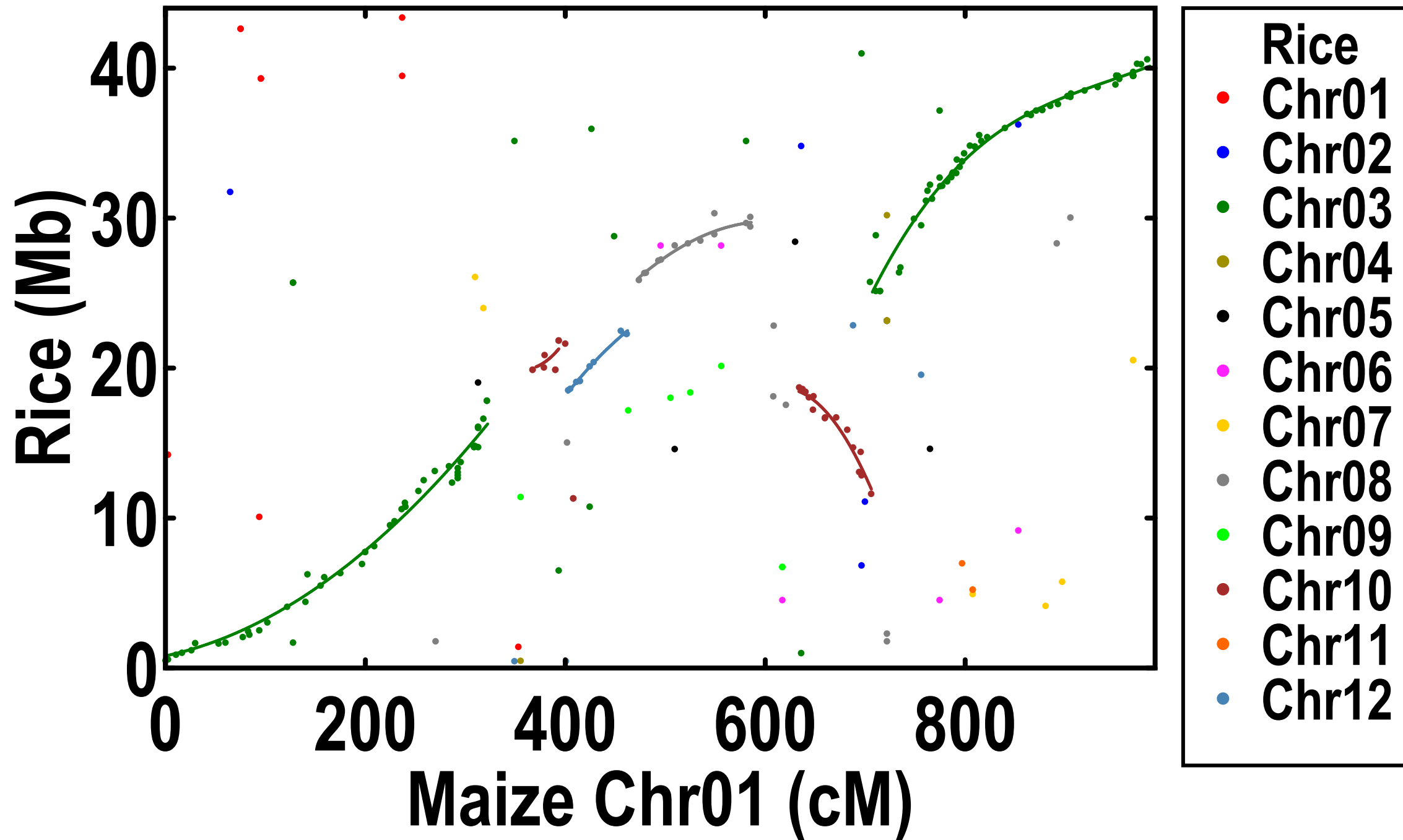

Supplement: Figure S9 — Each point indicates the genomic positions for a maize genetic marker and its highest confidence match in rice. The x-axis shows a specific chromosome for one genome, and the y-axis shows all chromosomes for a second genome, with the chromosome numbers color-coded as per the legend. We show here ten panels for maize. (288 KB ZIP). [file pbio.0030038.sg009.zip › Maize/Rice_Maize.Chr01.pdf]

# Maize-Rice Comparison

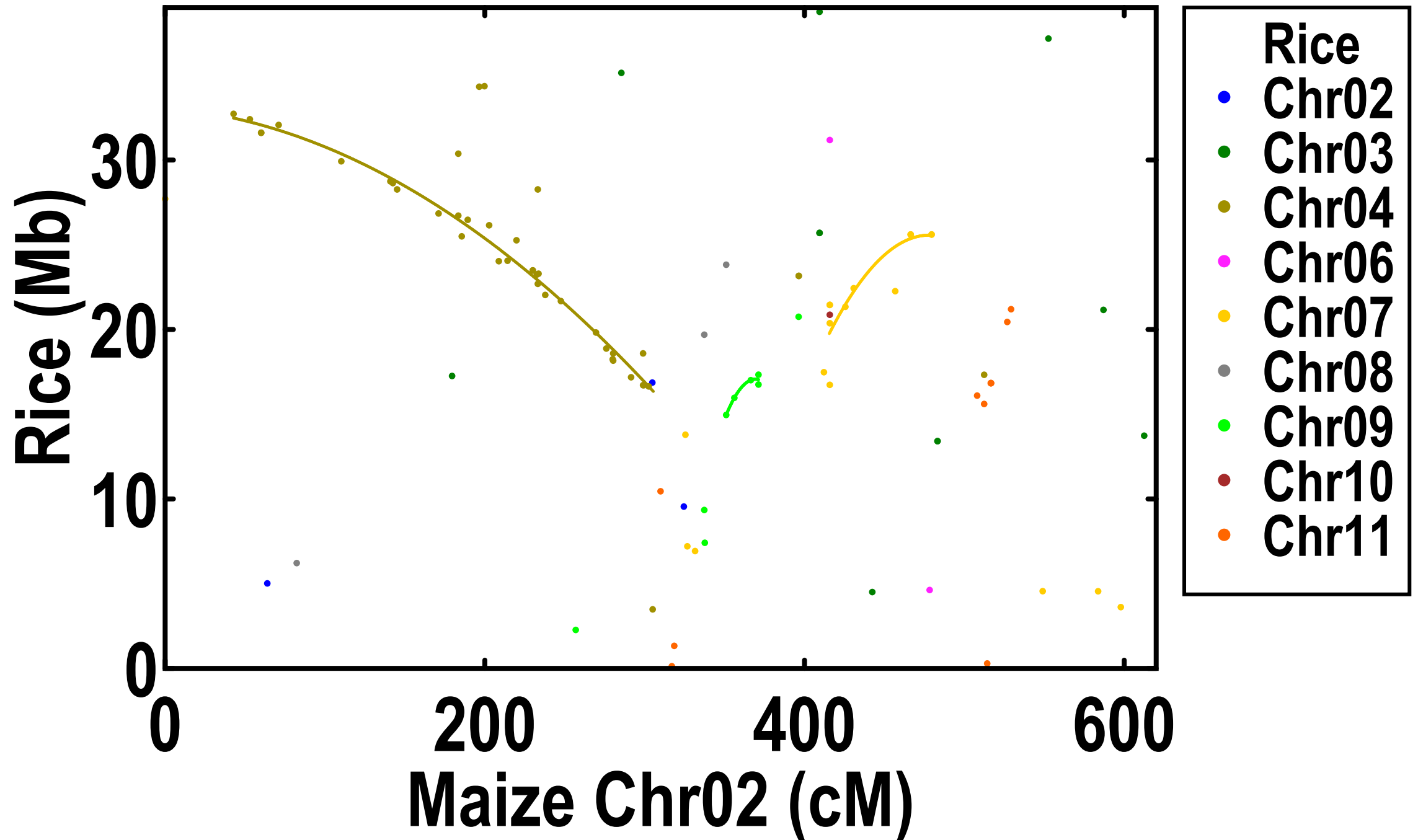

Supplement: Figure S9 — Each point indicates the genomic positions for a maize genetic marker and its highest confidence match in rice. The x-axis shows a specific chromosome for one genome, and the y-axis shows all chromosomes for a second genome, with the chromosome numbers color-coded as per the legend. We show here ten panels for maize. (288 KB ZIP). [file pbio.0030038.sg009.zip › Maize/Rice_Maize.Chr02.pdf]

# Maize-Rice Comparison

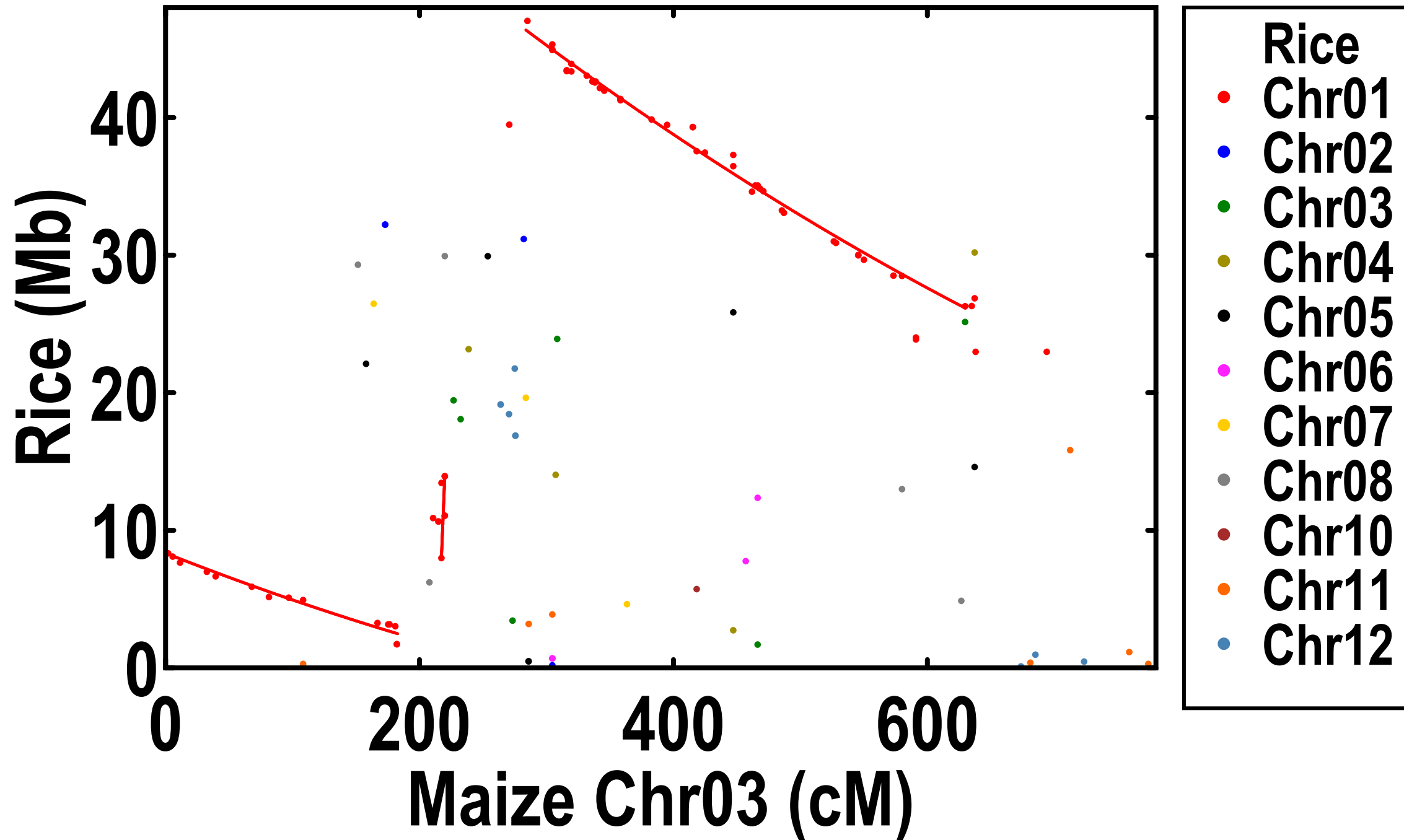

Supplement: Figure S9 — Each point indicates the genomic positions for a maize genetic marker and its highest confidence match in rice. The x-axis shows a specific chromosome for one genome, and the y-axis shows all chromosomes for a second genome, with the chromosome numbers color-coded as per the legend. We show here ten panels for maize. (288 KB ZIP). [file pbio.0030038.sg009.zip › Maize/Rice_Maize.Chr03.pdf]

# Maize-Rice Comparison

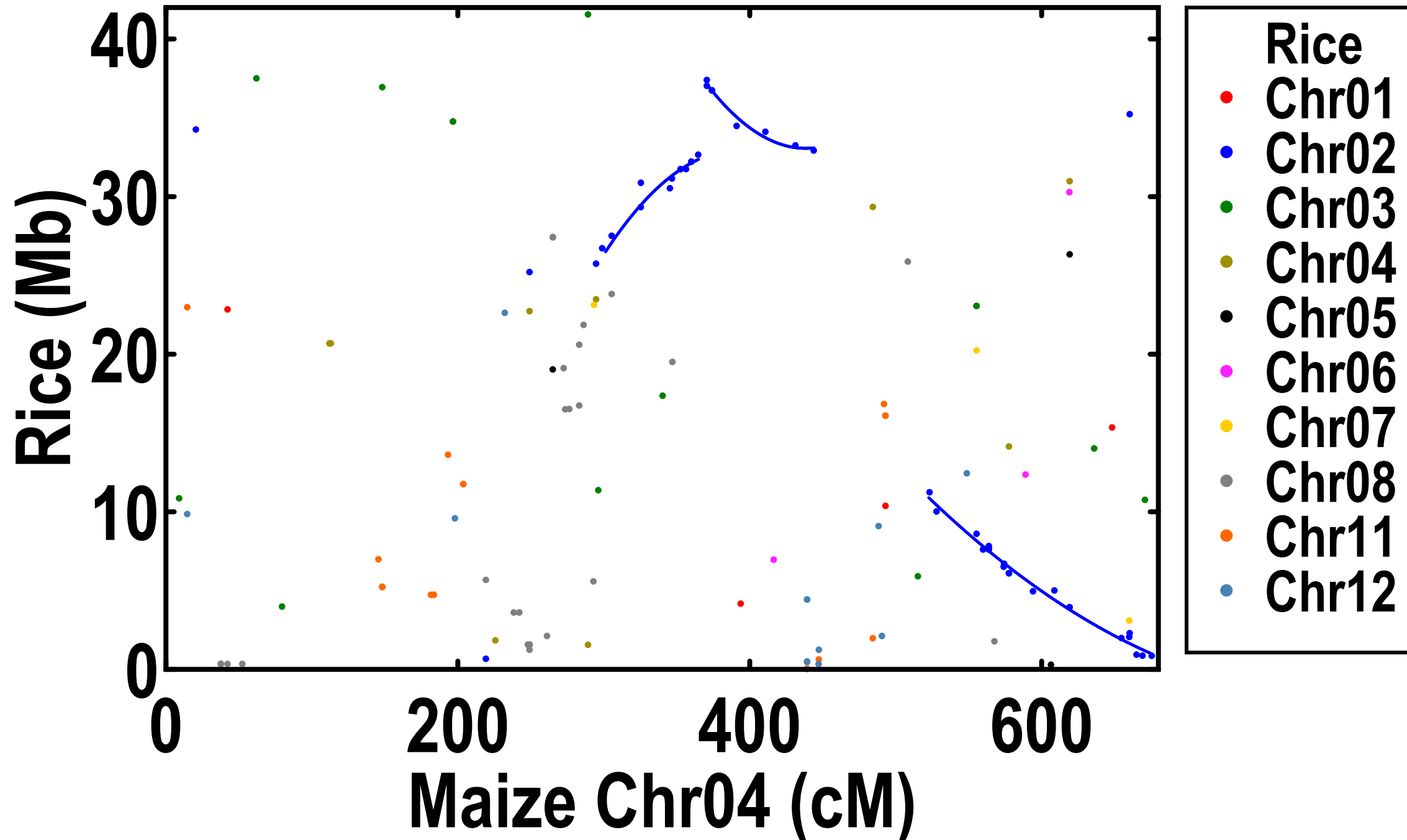

Supplement: Figure S9 — Each point indicates the genomic positions for a maize genetic marker and its highest confidence match in rice. The x-axis shows a specific chromosome for one genome, and the y-axis shows all chromosomes for a second genome, with the chromosome numbers color-coded as per the legend. We show here ten panels for maize. (288 KB ZIP). [file pbio.0030038.sg009.zip › Maize/Rice_Maize.Chr04.pdf]

# Maize-Rice Comparison

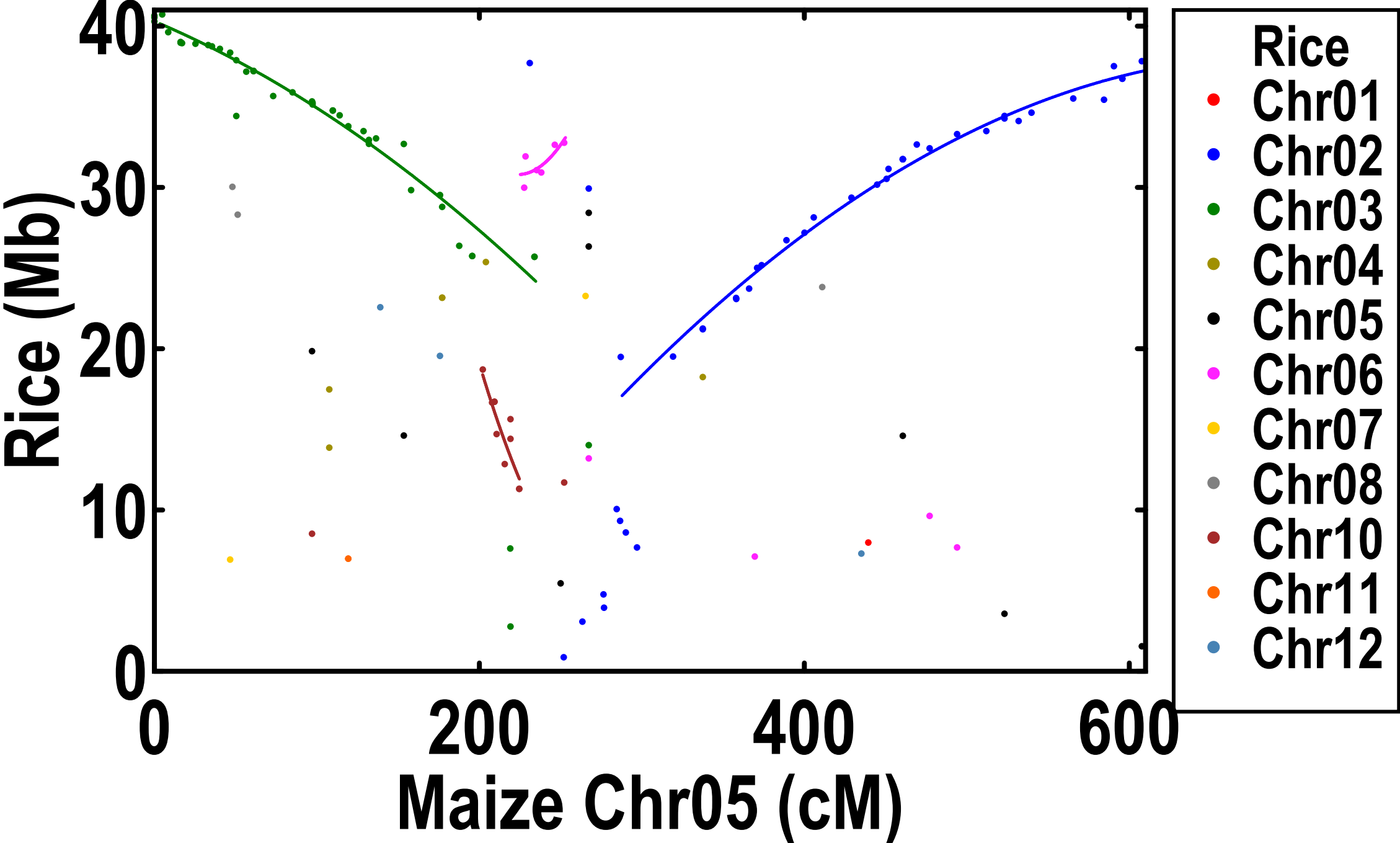

Supplement: Figure S9 — Each point indicates the genomic positions for a maize genetic marker and its highest confidence match in rice. The x-axis shows a specific chromosome for one genome, and the y-axis shows all chromosomes for a second genome, with the chromosome numbers color-coded as per the legend. We show here ten panels for maize. (288 KB ZIP). [file pbio.0030038.sg009.zip › Maize/Rice_Maize.Chr05.pdf]

# Maize-Rice Comparison

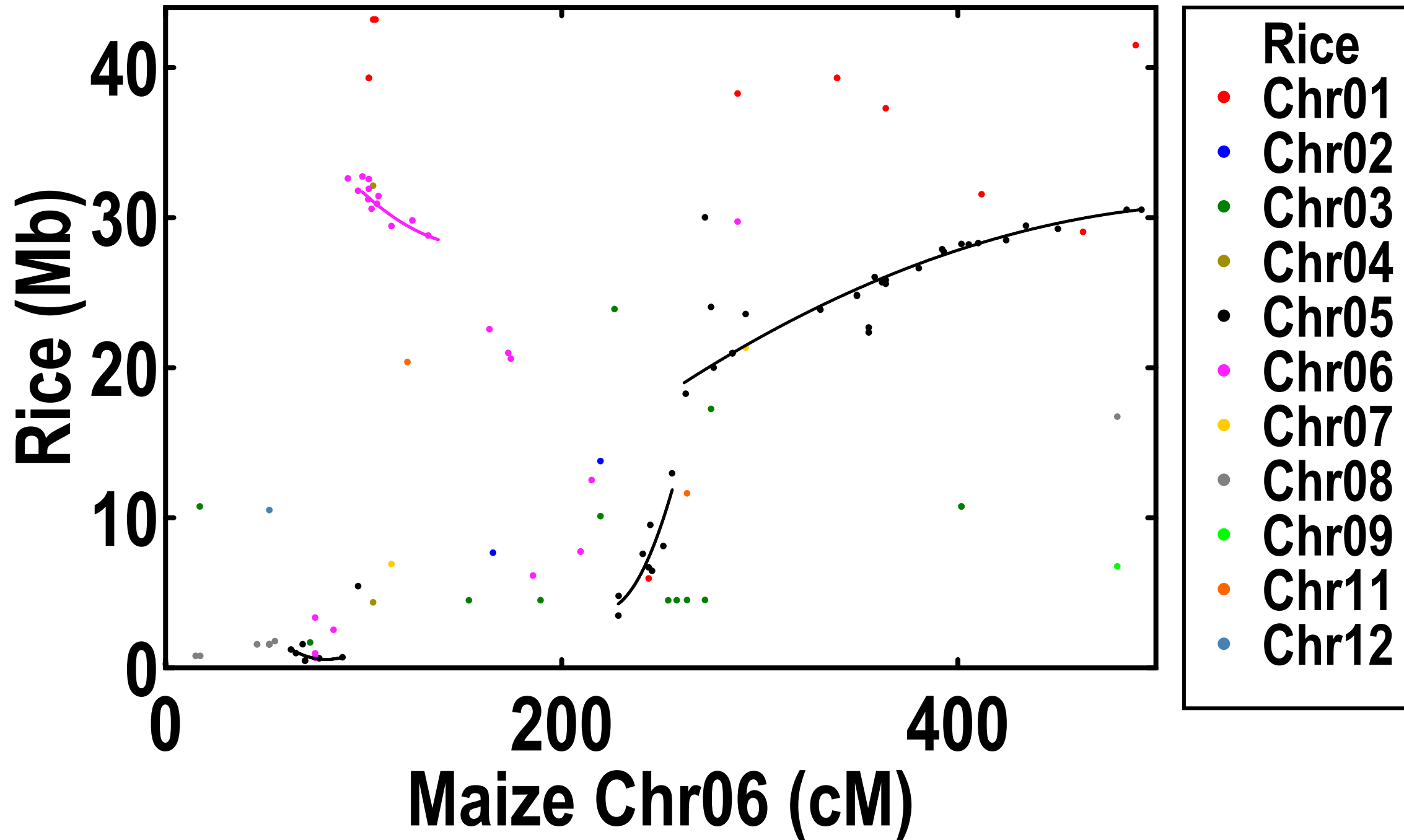

Supplement: Figure S9 — Each point indicates the genomic positions for a maize genetic marker and its highest confidence match in rice. The x-axis shows a specific chromosome for one genome, and the y-axis shows all chromosomes for a second genome, with the chromosome numbers color-coded as per the legend. We show here ten panels for maize. (288 KB ZIP). [file pbio.0030038.sg009.zip › Maize/Rice_Maize.Chr06.pdf]

# Maize-Rice Comparison

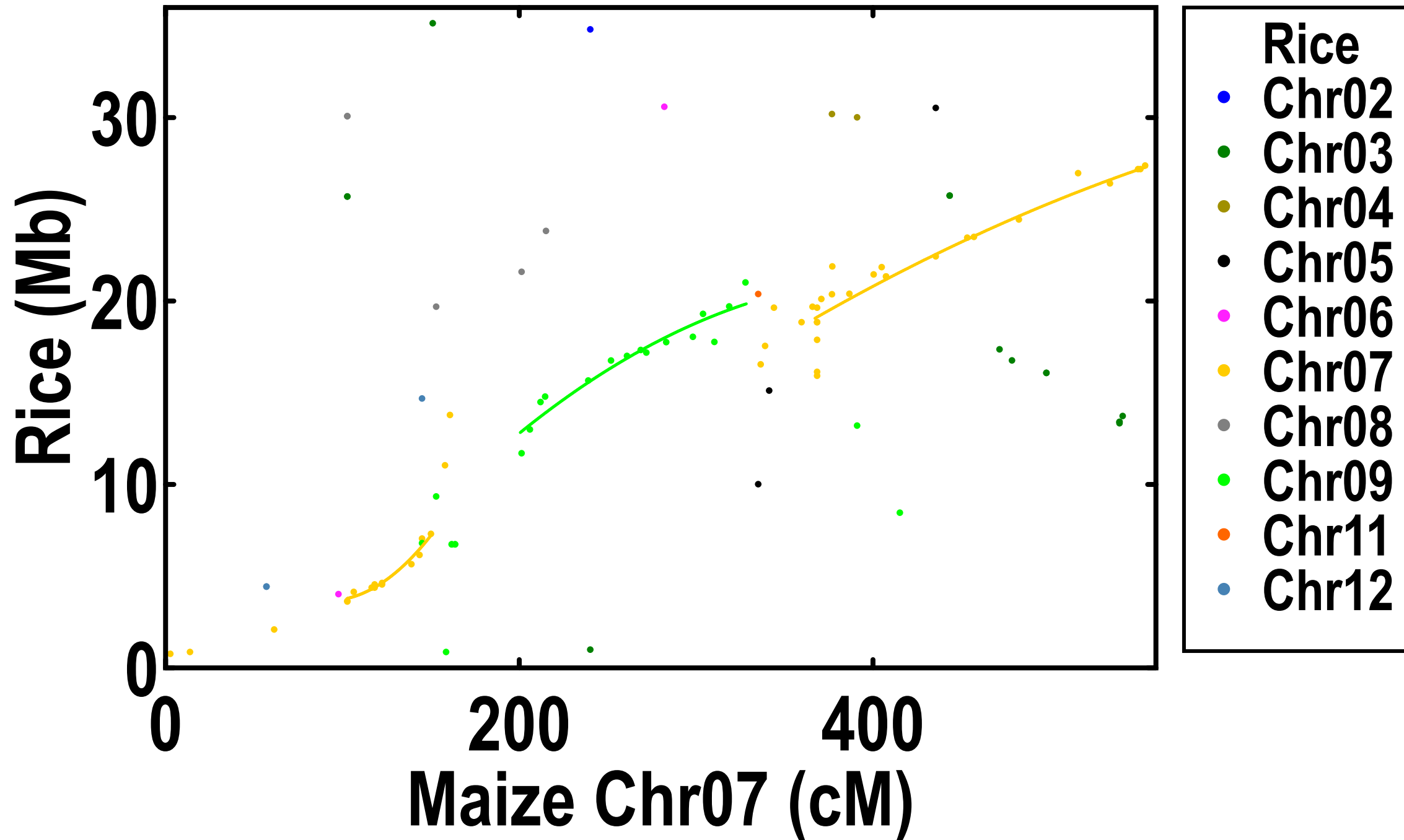

Supplement: Figure S9 — Each point indicates the genomic positions for a maize genetic marker and its highest confidence match in rice. The x-axis shows a specific chromosome for one genome, and the y-axis shows all chromosomes for a second genome, with the chromosome numbers color-coded as per the legend. We show here ten panels for maize. (288 KB ZIP). [file pbio.0030038.sg009.zip › Maize/Rice_Maize.Chr07.pdf]

# Maize-Rice Comparison

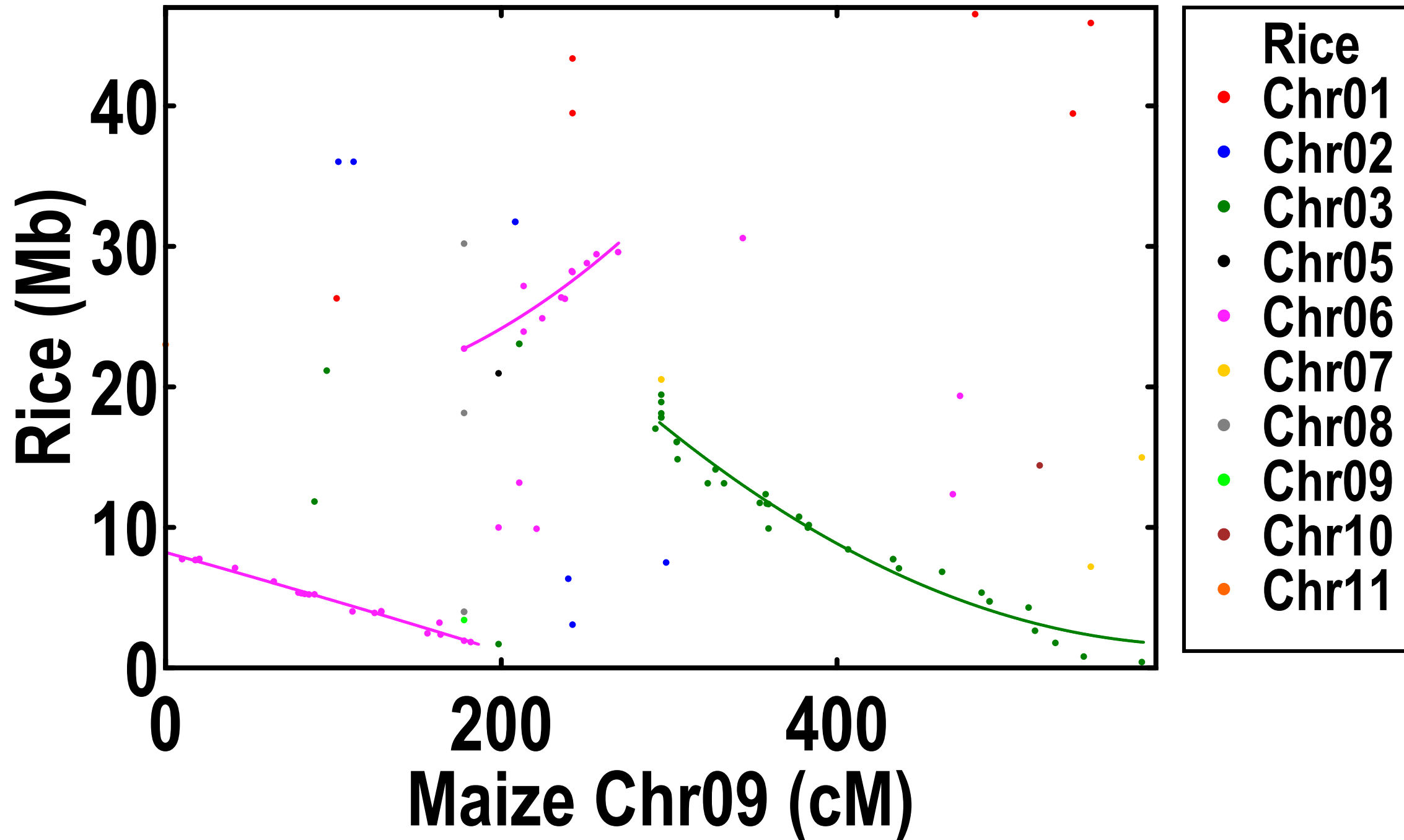

Supplement: Figure S9 — Each point indicates the genomic positions for a maize genetic marker and its highest confidence match in rice. The x-axis shows a specific chromosome for one genome, and the y-axis shows all chromosomes for a second genome, with the chromosome numbers color-coded as per the legend. We show here ten panels for maize. (288 KB ZIP). [file pbio.0030038.sg009.zip › Maize/Rice_Maize.Chr09.pdf]

# Maize-Rice Comparison

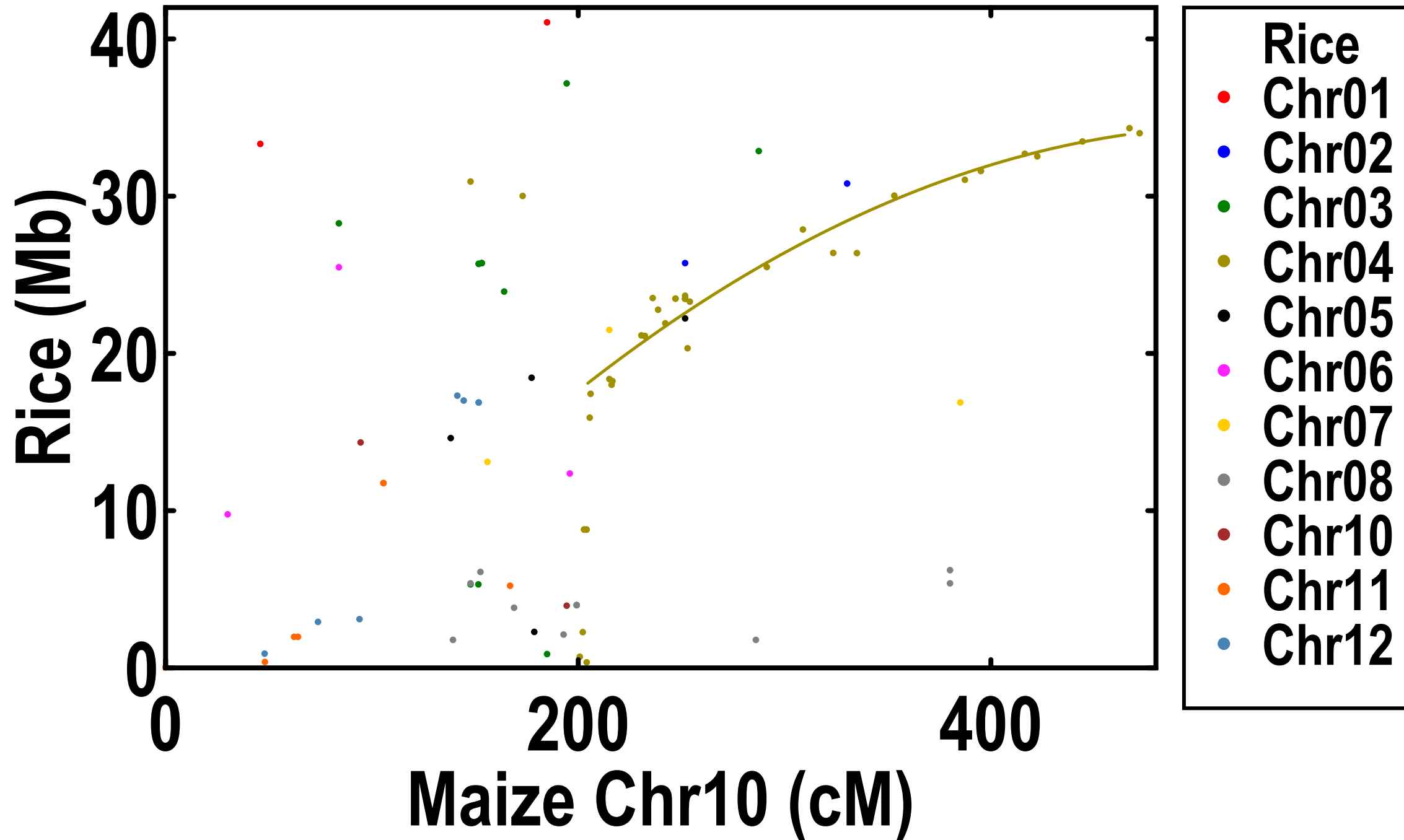

Supplement: Figure S9 — Each point indicates the genomic positions for a maize genetic marker and its highest confidence match in rice. The x-axis shows a specific chromosome for one genome, and the y-axis shows all chromosomes for a second genome, with the chromosome numbers color-coded as per the legend. We show here ten panels for maize. (288 KB ZIP). [file pbio.0030038.sg009.zip › Maize/Rice_Maize.Chr10.pdf]
