# Supplementary material for: The Genomes of Oryza sativa: A History of Duplications
Source: PLoS Biol. 2005 Feb 1;3(2):e38. doi: 10.1371/journal.pbio.0030038 (PMC546038; doi:10.1371/journal.pbio.0030038)
Supplement: Figure S6 — We depict all the genetic markers, nr-KOME cDNAs, FGENESH gene predictions, and transposable elements identified by RepeatMasker. Genes are depicted as WH (colored blue) or NH (colored red) based on their similarity to Arabidopsis. TEs are decomposed into classes I, II, and III. Correspondence between indica and japonica is indicated by drawing a connecting line between the 5′ ends of the nr-KOME cDNAs that clearly align to both assemblies. (9.6 MB ZIP). [file Chr08_L.pdf › two_piece/Chr08_L.pdf]

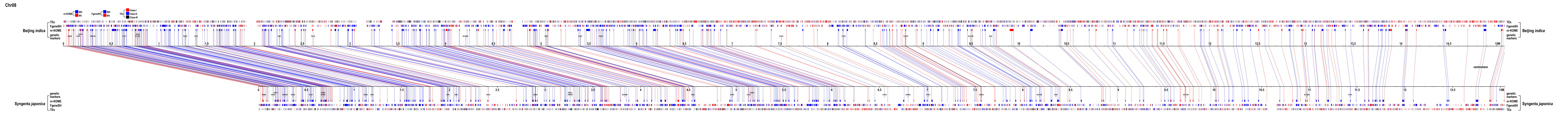
Chr08
nr-KOME
WH
NH
FgeneSH
WH
NH
TEs
Class-I
Class-II
Class-III
Beijing indica
TEs
FgeneSH
nr-KOME
genetic markers
centromere
Syngenta japonica
genetic markers
nr-KOME
FgeneSH
TEs
