# Supplementary material for: The Genomes of Oryza sativa: A History of Duplications
Source: PLoS Biol. 2005 Feb 1;3(2):e38. doi: 10.1371/journal.pbio.0030038 (PMC546038; doi:10.1371/journal.pbio.0030038)
Supplement: Figure S9 — Each point indicates the genomic positions for a maize genetic marker and its highest confidence match in rice. The x-axis shows a specific chromosome for one genome, and the y-axis shows all chromosomes for a second genome, with the chromosome numbers color-coded as per the legend. We show here ten panels for maize. (288 KB ZIP). [file Rice_Maize.Chr08.pdf › Maize/Rice_Maize.Chr08.pdf]

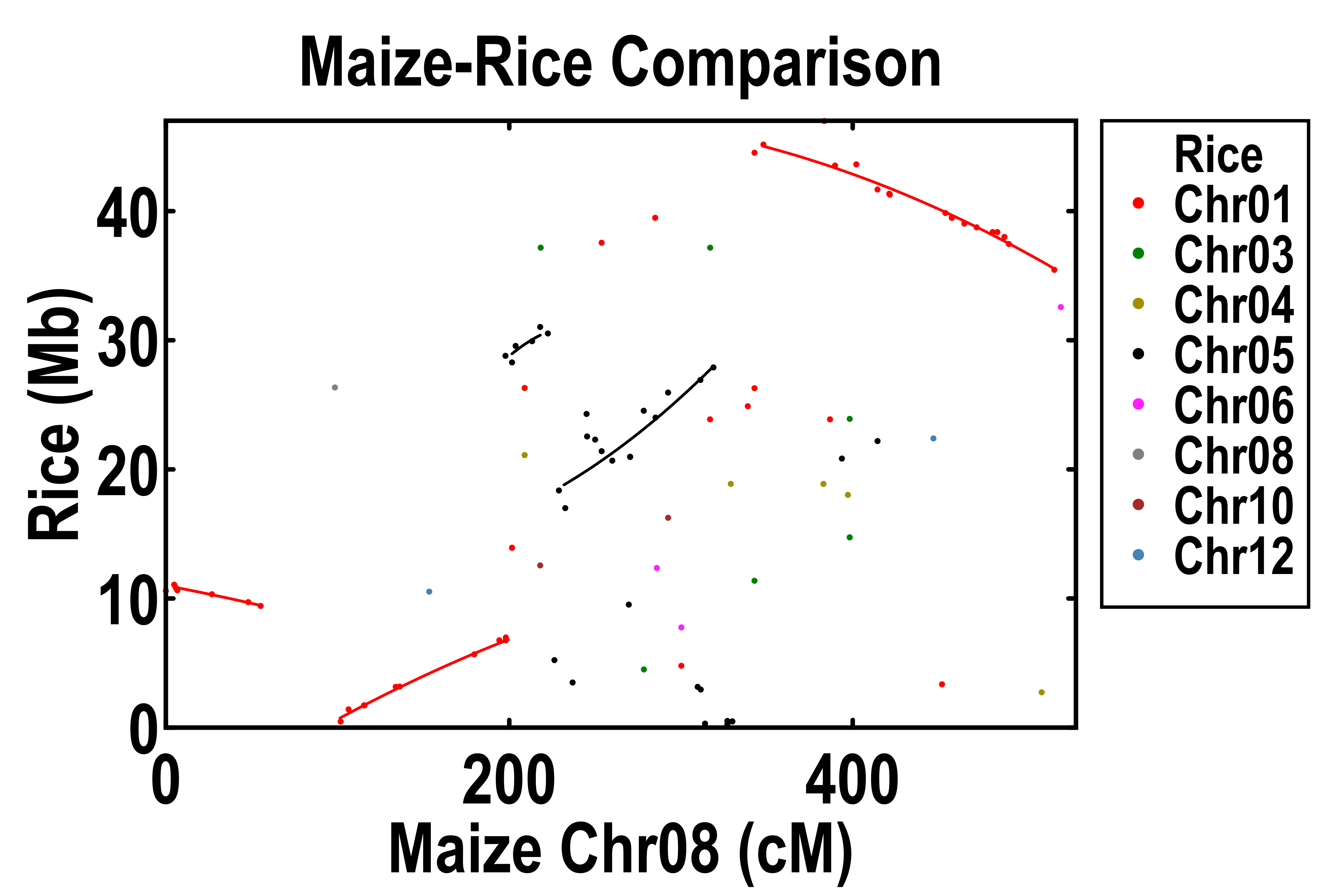

Maize-Rice Comparison
Rice (Mb)
Maize Chr08 (cM)
0
10
20
30
40
0
200
400
Rice
Chr01
Chr03
Chr04
Chr05
Chr06
Chr08
Chr10
Chr12
